# Global Phylogeographic and Admixture Patterns in Grey Wolves and Genetic Legacy of An Ancient Siberian Lineage

**DOI:** 10.1038/s41598-019-53492-9

**Published:** 2019-11-22

**Authors:** Małgorzata Pilot, Andre E. Moura, Innokentiy M. Okhlopkov, Nikolay V. Mamaev, Abdulaziz N. Alagaili, Osama B. Mohammed, Eduard G. Yavruyan, Ninna H. Manaseryan, Vahram Hayrapetyan, Natia Kopaliani, Elena Tsingarska, Miha Krofel, Pontus Skoglund, Wiesław Bogdanowicz

**Affiliations:** 1 School of Life Sciences, University of Lincoln, Lincoln, United Kingdom; 2 Museum and Institute of Zoology, Polish Academy of Sciences, Warsaw, Poland; 3 Institute of Biological Problems of Cryolithozone, Siberian Branch of Russian Academy of Sciences, Yakutsk, Russia; 4 KSU Mammals Research Chair, Department of Zoology, King Saud University, Riyadh, Saudi Arabia; 5 Scientific Center of Zoology and Hydroecology, National Academy of Sciences, Yerevan, Armenia; 6 Green Artsakh Biosphere Complex SNCO, Syunik, Armenia; 7 Institute of Ecology, Ilia State University, Tbilisi, Georgia; 8 BALKANI Wildlife Society, Sofia, Bulgaria; 9 Department of Forestry, Biotechnical Faculty, University of Ljubljana, Ljubljana, Slovenia; 10 Francis Crick Institute, London, United Kingdom

**Keywords:** Phylogenetics, Evolutionary biology

## Abstract

The evolutionary relationships between extinct and extant lineages provide important insight into species’ response to environmental change. The grey wolf is among the few Holarctic large carnivores that survived the Late Pleistocene megafaunal extinctions, responding to that period’s profound environmental changes with loss of distinct lineages and phylogeographic shifts, and undergoing domestication. We reconstructed global genome-wide phylogeographic patterns in modern wolves, including previously underrepresented Siberian wolves, and assessed their evolutionary relationships with a previously genotyped wolf from Taimyr, Siberia, dated at 35 Kya. The inferred phylogeographic structure was affected by admixture with dogs, coyotes and golden jackals, stressing the importance of accounting for this process in phylogeographic studies. The Taimyr lineage was distinct from modern Siberian wolves and constituted a sister lineage of modern Eurasian wolves and domestic dogs, with an ambiguous position relative to North American wolves. We detected gene flow from the Taimyr lineage to Arctic dog breeds, but population clustering methods indicated closer similarity of the Taimyr wolf to modern wolves than dogs, implying complex post-divergence relationships among these lineages. Our study shows that introgression from ecologically diverse con-specific and con-generic populations was common in wolves’ evolutionary history, and could have facilitated their adaptation to environmental change.

## Introduction

The Late Pleistocene was a period of numerous extinctions of large mammals, resulting from the combined effect of climate change and human impact^1^. Those species that survived the megafaunal extinctions experienced demographic bottlenecks, local extinctions and phylogeographic shifts^2–4^ – the events that were also reported to precede extinctions in multiple species^5–8^. Palaeogenomic studies revealed the presence of genomic fragments originating from extinct species in gene pools of their extant relatives^9–11^. This may point out to genetic swamping as a contributing factor of these extinctions, although some populations carry hybridisation-derived gene variants showing signatures of positive selection^9,12,13^. This suggests that genetic contribution from extinct species could have facilitated adaptation of their extant relatives to rapidly changing environmental conditions. Reconstruction of the evolutionary relationships between extinct and extant lineages can thus provide a direct insight into complex evolutionary pathways towards extinction or survival of a species.

The grey wolf (*Canis lupus*) is among the few large carnivores that survived the Late Pleistocene megafaunal extinctions. Similar to many other megafaunal species, the wolf experienced a global population decline at the end of the Pleistocene^14,15^, which was associated with extinctions of distinct ecomorphs and phylogeographic shifts^16–19^. During that period, one of the wolf lineages evolved into the domestic dog (*Canis lupus familiaris*; reviewed in^20^). The domestication ensured the evolutionary success of this lineage via expansion into a new ecological niche, while substantially affecting non-domesticated populations via resource competition, disease transfer and admixture^21,22^. Wolf populations also carry signatures of both past and ongoing admixture with golden jackals (*Canis aureus*) in Eurasia^23–25^ and coyotes (*Canis latrans*) in North America^26–28^.

All extant wolves have a recent common ancestry estimated at about 32 Kya – a date coinciding with the onset of global demographic decline of the species^15^. Studies based on whole nuclear genome sequence data revealed that modern Eurasian wolves form a monophyletic clade distinct from North American wolves^15,28^, while mitochondrial genomes show a more complex phylogeographic pattern^18^. A recent analysis of mitochondrial genomes of ancient and modern wolves inferred that modern wolf populations can be traced back to a single expansion event originating about 25 Kya in Beringia (the region including east Siberia, Alaska and part of the Yukon, all previously connected by land)^19^. Ancient wolves from other parts of the grey wolf distribution carried distinct mitochondrial lineages not represented in contemporary wolf populations, implying extinction and replacement of these lineages^18,19,29^.

Accordingly, the nuclear genome of a wolf from the Taimyr Peninsula in Siberia, dated at 34.9 Kya, was phylogenetically distinct from genomes of extant wolves and dogs, implying that this individual represents an extinct divergent lineage^10^. In contrast, the mitochondrial genome of this individual clustered with modern wolves^10^, which could result from post-divergence gene flow. Furthermore, Arctic dog breeds were shown to carry signatures of past admixture with the Taimyr wolf lineage^10^. However, Arctic dog breeds also show signatures of admixture with modern wolves^30^, implying either independent admixture episodes occurring at different times, or continuity of the Taimyr lineage in Siberian wolves. A better understanding of the genetic legacy of the Taimyr wolf lineage requires comparison with comprehensive data on modern wolves, but representation of modern Asian wolves in published genetic studies is very limited, with Siberia being particularly underrepresented. The proposed Beringian origin of modern wolves^19^ should have resulted in genetic similarity between modern populations from East Siberia and Alaska (former parts of Beringia), but these populations were not included in previous studies on global wolf phylogeography. Therefore, knowledge of phylogenetic patterns in modern wolves from Asia, and from Siberia in particular, is needed to better understand the complex history of this species.

**Figure 1:**
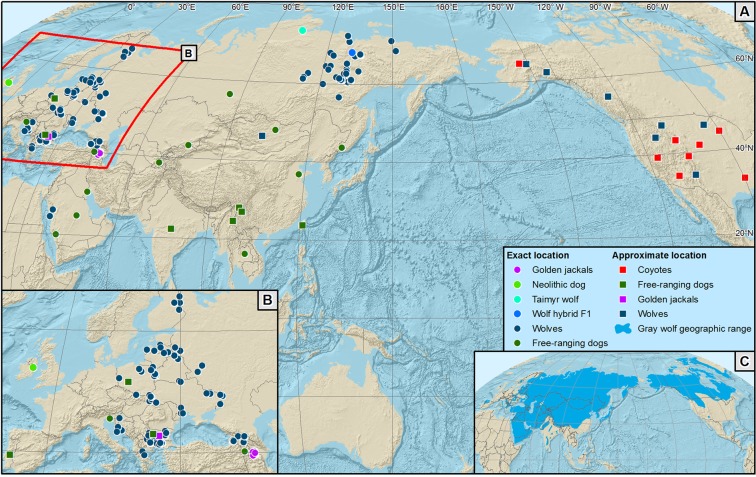
Distribution of wild canids and free-ranging dogs analysed in this study. (**A**) Sample distribution across the Holarctic, (**B**) sample distribution in Europe, (**C**) distribution range of the grey wolf. Each symbol represents multiple individuals sampled in the same locations. The map does not include samples of black backed jackals (from Namibia), free-ranging dogs from North America (with unknown sampling locations) and pure-bred dogs. For the list of all samples analysed in the study, see Supplementary Table 1. Software used to generate the map: ESRI 2014. ArcGIS Desktop: Release 10.2.2. Redlands, CA: Environmental Systems Research Institute. Shaded relief generated from SRTM DEM 90 m^63^. Countries marked using Natural Earth data (Free vector and raster map data @ naturalearthdata.com). All versions of Natural Earth raster and vector map data are in the public domain. They can be used in any manner without a licence, including modifying the content and design, electronic dissemination, and offset printing. Gray wolf geographic range drawn according to Boitani *et al*.^64^.

In this study we reconstruct genome-wide phylogeographic patterns in modern grey wolf populations across their Holarctic distribution, based on an extensive sample set that includes previously underrepresented Siberian wolves (Fig. 1). We also provide the first global reconstruction of wolf admixture patterns with domestic dogs, coyotes and golden jackals. We use these data to test the following hypotheses:

1. There is no continuity between the Late Pleistocene Taimyr lineage and modern wolf lineages in Eurasia^10,19^. If this is the case, modern Siberian wolves should cluster with other modern wolf populations rather than with the Taimyr wolf.
2. All modern wolves originated from a single Beringian expansion^19^. This should result in a gradual increase of linkage disequilibrium with increasing distance from East Siberia and Alaska.
3. Wolf admixture in Arctic dog breeds originates from the Taimyr lineage^10^. This should result in inference of gene flow from the Taimyr wolf rather than from modern Siberian wolves into Arctic breeds.
4. Eurasian grey wolves and golden jackals experienced ancient admixture^23,25^. In this scenario, ancestry blocks originating from the golden jackal should be detected in Eurasian grey wolves.
5. Phylogeographic inference in modern grey wolves is affected by admixture episodes with other canids. In this case, we expect the topology of inferred ancestry relationships between wolf and other canid lineages to change once admixture episodes are taken into account.

## Results

### Heterozygosity and linkage disequilibrium patterns in worldwide wolf populations

**Figure 2:**
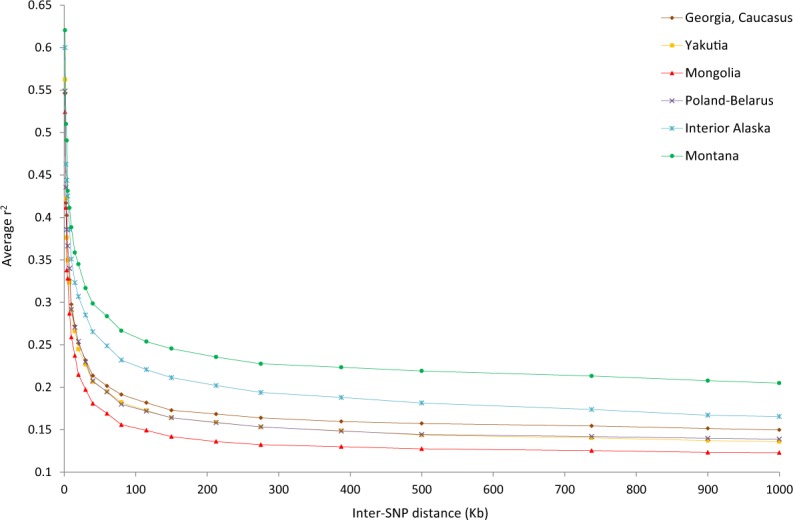
Linkage disequilibrium patterns in wolf populations. Linkage disequilibrium is represented by average genotypic association coefficient r^2^ and is plotted as a function of inter-SNP distance. The estimate was carried out for 10 unrelated individuals per population. The data for Mexican wolves were excluded from the plot for clarity, and are presented instead in Supplementary Fig. 1.

We found that all Asian wolf populations had similar levels of genome-wide heterozygosity (H_o_ = 0.27), which were comparable to those in European populations and higher than in North American populations (Supplementary Table 2). Mongolian wolves had lower linkage disequlibrium (LD) than all other populations representing the main geographic regions of Eurasia and North America, throughout all distance classes assessed (Fig. 2). The populations from regions corresponding to east and west Beringia (Interior Alaska and Yakutia, respectively) had considerably higher LD across all distance classes compared to Mongolian wolves. LD levels in North American populations were higher than in Eurasian populations, and increased along a north-south gradient across the North American continent. In contrast, Eurasian populations did not show a single geographical gradient of LD patterns (Fig. 2, Supplementary Fig. 1).

### Admixture patterns in the global wolf population detected from population clustering analyses

We used Admixture^31^ to assess the genetic structure in worldwide wolf populations, taking into account their admixture with other wild canids and dogs. This analysis, as well as other analyses described below (unless stated otherwise) were based on the set of 42,320 SNP loci, obtained after removing loci not genotyped in the Taimyr wolf in order to maximise the accuracy of the comparison of this ancient wolf with modern populations.

The Admixture analysis distinguished dogs from wild canids at K = 2 and wolves from other wild canids (coyotes, golden jackals and black backed jackals) at K = 3. Increasing K values resulted in sub-clustering within wolf and dog populations rather than separation of the other wild canid species. These species clustered together due to small sample sizes (see below). No optimum K could be identified between K = 1 and K = 15, with the cross-validation error continuously declining with the increase of K. This does not imply a lack of structure, but reflects sub-clustering within the multiple canid species studied.

At K = 3, the Taimyr wolf clustered with modern wolves (with assignment value of 0.74), but showed positive assignment values to the dog (0.09) and coyote/jackal clusters (0.17). For K values between 4 and 15, the assignment values of the Taimyr wolf showed limited variation: 0.731–0.775 for grey wolf clusters, 0.075–0.107 for dog clusters and 0.131–0.168 for coyote/jackal clusters (Supplementary Table 3). Our dataset included structured populations of multiple species, and thus deviated from the assumptions of the Admixture model that all individuals originate from homogeneous populations and each population is well represented in the analysed dataset^32^. The mixed ancestry inferred for the Taimyr wolf indicates complex relationships between the Taimyr lineage and contemporary representatives of the genus *Canis*, but the estimated admixture proportions should not be interpreted as the actual ancestry of this individual.

**Figure 3:**
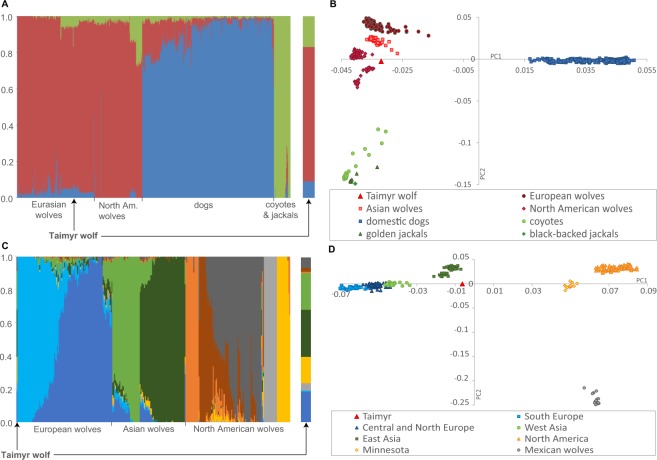
Population structure inferred for all canids studied (**A**,**B**) and wolves only (**C**,**D**) using Admixture and Eigensoft.

At K = 3, modern Asian wolves had positive assignment to the dog cluster (0.02–0.13) and the jackal/coyote cluster (0.01–0.13) (Fig. 3A, Supplementary Fig. 2A, Supplementary Table 4). European wolves displayed large variation in assignment values to the dog cluster, and their assignment to the jackal/coyote cluster was no higher than 0.02. Mexican wolves (the population currently inhabiting New Mexico and Arizona) had small but positive assignment values to the dog cluster, and higher to the jackal/coyote cluster (0.13–0.15). Wolves from Minnesota had assignment values to the jackal/coyote cluster higher than 0.2, while the remaining North American populations had no more than 0.05 of the inferred coyote ancestry and no signature of dog admixture with the exception of two individuals. The term “North American wolves” will be applied thereafter to the North American populations excluding Mexican and Minnesota wolves, which display distinct admixture and population structure patterns.

Some individual wild canids had admixture levels suggesting that they are hybrids or recent-generation backcrosses. This included a black wolf-like individual from Yakutia (the first case of black coloration in this region known to us), two individuals from Idaho and British Columbia sampled as wolves, and a golden jackal from Nagorny Karabakh in the Caucasus, which had a small but detectable wolf admixture (Fig. 3, Supplementary Table 4).

Dog breeds and free-ranging dogs varied in their assignment values to the wolf cluster, with highest values occurring in dogs of East Asian and Arctic origin (Supplementary Table 4). Breeds originating from known recent admixture with wolves had assignment values to the wolf cluster of 0.15–0.17 (Czechoslovakian wolfdog) and 0.23–0.34 (Saarloos wolfdog).

Following the assessment of global admixture patterns, we carried out an Admixture analysis including only wolves. This analysis distinguished Eurasian and North American wolves at K = 2, and West Eurasian, East Asian and North American wolves at K = 3. The optimum K was 9, distinguishing two regional populations in Europe, two in Asia, and five in North America (Fig. 3C, Supplementary Fig. 2B). At K = 9, the Taimyr wolf was assigned with highest probabilities to four wolf populations: East Asia (0.29), West Asia (0.22), North-east Europe (0.18) and Minnesota (0.16), with the remaining five clusters having low assignment values (0.15 altogether). In contrast, the assignment values of contemporary Asian wolves to North American populations were no higher than 0.016.

### Genetic structure of the worldwide wolf population inferred from principal component analysis

The PCA (carried out using 42,320 loci) revealed similar clustering patterns as Admixture, with distinct clusters of grey wolves and dogs, and the other *Canis* species clustered together (Fig. 3B, Supplementary Fig. 3A). The positions of Eurasian wolves on the PCA plot corresponded to their geographic distances, but the geographic distances between Eurasian and North American populations were not reflected in their genetic differentiation. All North American wolves clustered together except populations from Minnesota and Mexico, which showed closer proximity to the coyote/jackal cluster. When only wolves were included in the analysis, Eurasian and North American wolves were distinguished on PC1, and Mexican wolves were distinguished from other North American wolves on PC2 (Fig. 3D, Supplementary Fig. 4).

The PCA analysis carried out after inclusion of additional golden jackals and black backed jackal samples (to obtain more balanced sample sizes) resulted in three clearly distinct clusters of coyotes, golden jackals and blacked backed jackals (Supplementary Fig. 5). This implies that these species clustered together in the original dataset due to unbalanced sample sizes.

The position of the Taimyr wolf on the PCA plot was strongly influenced by the SNP set analysed and the composition of the reference populations. In the PCA based on the dataset including SNP loci with missing data for the Taimyr wolf (90,554 loci), this individual was placed centrally in the plot, with similar distances to clusters of wolves, dogs and coyotes (Supplementary Fig. 3B). Pruning the SNP loci missing from the Taimyr wolf resulted in its clustering with modern wolves, although not within any regional population (Fig. 3B, Supplementary Fig. 3A). When only wolves were included in the analysis, the Taimyr wolf was placed near, but not within, the cluster of modern East Asian wolves (Fig. 3D, Supplementary Fig. 4C). To maintain accuracy of inference regarding the Taimyr wolf, all the results reported below are based on the dataset where loci missing from this individual were removed from the analysed dataset.

### Reconstruction of ancestry relationships among wolf populations accounting for inter-specific gene flow

**Figure 4:**
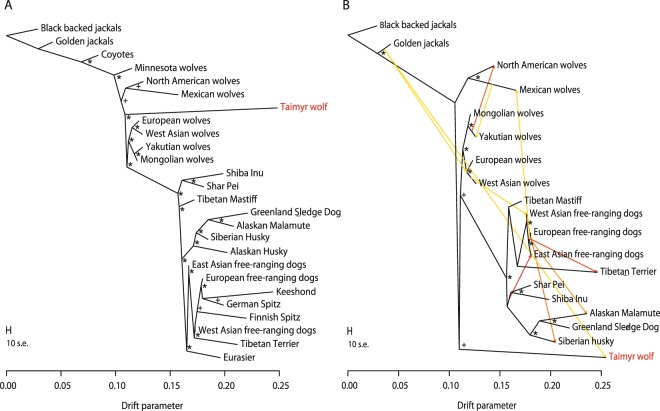
Ancestry relationships between the Taimyr wolf and modern wolf and dog populations inferred in TreeMix for (**A**) the whole set of canid populations, (**B**) a reduced population set, excluding those with known admixture history, and assuming 10 events of gene flow. Gene flow is represented by arrows, with colours reflecting the intensity of gene flow, from lowest (yellow) to highest (red). Nodes with 95–100% bootstrap support are marked with an asterisk, and nodes with bootstrap support 50–95% are marked with a plus symbol.

The Maximum Likelihood tree reconstructed using TreeMix^33^ showed that North American wolves diverged before the split between modern Eurasian wolves and domestic dogs. The Taimyr wolf was placed (with 100% bootstrap support) as the sister lineage of all Eurasian wolves and domestic dogs, with North American wolves branching before the split of the Taimyr lineage and modern Eurasian wolves (Fig. 4A, Supplementary Fig. 6).

The addition of 10 migration edges did not change the ancestry relationships between wolf populations, except for Minnesota wolves. The inclusion of a migration edge from coyotes resulted in the clustering of the Minnesota wolves with other North American wolves instead of forming a separate branch (Supplementary Fig. 6B,C). TreeMix also detected post-divergence gene flow from Yakutian to North American wolves, and from coyotes to the common ancestor of Eurasian wolves; however, this last result is unlikely given the long-term geographic isolation of these populations. The remaining migration events reflect admixture between different dog breeds or populations, including cases of admixture between dog breeds that are consistent with the history of breed formation (e.g. Czechoslovakian wolfdogs). The jackknife analysis provided strong support for all migration events, with *P* < 3 × 10^−10^ in all cases.

We then repeated the analysis for a reduced dataset obtained by excluding populations involved in admixture events outside Eurasia or between dog breeds (coyotes, Minnesota wolves, Alaskan Huskies, Eurasiers and European spitz-type dog breeds). In this new analysis, the bootstrap support for the Taimyr wolf branching after the North American wolves declined from 100% to 56% (Fig. 4B), indicating that the relative positions of these two lineages may be influenced by the admixture of North American wolves with coyotes. This new analysis inferred gene flow from golden jackals to the Taimyr wolf and from golden jackals to the common ancestor of West/Central Asian and European wolves. We also identified gene flow from Yakutian to North American wolves and from West Asian wolves to West Asian dogs (Fig. 4B, Supplementary Fig. 7). All the inferred gene flow events had statistical support of *P* < 0.03 except for the inferred gene flow from European dogs to Tibetan Terrier. An additional analysis, where we included coyotes but excluded other populations and breeds mentioned above gave mostly consistent results with those described above, but the gene flow from golden jackals was no longer detected (Supplementary Fig. 8).

The analysis including only canids originating in Asia, and assuming 10 migration edges, revealed gene flow from the Taimyr lineage to the ancestral lineage of Arctic dog breeds: Siberian Husky, Alaskan Malamute and Greenland Sledge Dog. Arctic breeds have also received gene flow from modern East Asian wolves and from the ancestral lineage of Asian free-ranging dogs (Supplementary Fig. 9).

### Admixture inferred from ancestry blocks analysis in Lamp

The proportion of chromosomal blocks of dog ancestry in individual wolves estimated in Lamp^34^ (assuming 10 generations since admixture) varied between 0 and 27%, and the proportion of chromosomal blocks of wolf ancestry in individual dogs varied between 0 and 7% (Table 1, Supplementary Table 5, Supplementary Fig. 10). The Neolithic Irish dog had 0.17% of estimated wolf ancestry. Wolfdog breeds known to originate from recent human-mediated admixture between wolves and dogs had considerable proportion of wolf ancestry: 11–12% in Czechoslovakian wolfdog and 18–33% in Saarloos wolfdog. Five wild canids, sampled as wolves, had proportions of wolf ancestry between 35% and 73%.

**Table 1:** Proportion of dog and golden jackal ancestry in modern and ancient canid populations inferred using Admixture and Lamp for autosomal chromosomes. Admixture software infers long-term patterns of admixture, while the Lamp analysis was run assuming admixture in last 10 generations of ancestors. The Admixture results are reported for the analysis assuming 3 genetic clusters. The results of the Admixture analysis for the Taimyr wolf assuming larger numbers of clusters are presented in the Supplementary Fig. 3. The Lamp results are reported as mean values from all autosomal chromosomes.

Population	Dog ancestry Admixture	Golden jackal^†^ ancestry Admixture	Dog ancestry Lamp	Golden jackal ancestry Lamp
Taimyr wolf	0.09	0.17	0.03	0.02
modern Eurasian wolves	0.00–0.26	0.00–0.13	0.00–0.27	0.00–0.01
modern dogs	0.70–0.99	0.00–0.05	0.93–1.00	—
golden jackal	0.00–0.05	0.95–1.00	—	0.98–1.00

^†^Assignment to the cluster consisting of golden jackals as well as black backed jackals and coyotes, the last two absent from Eurasia.

The ancient Taimyr wolf showed 100% of wolf ancestry in 36 chromosomes, while putative dog ancestry was detected in chromosomes 15 and 23 (22% and 100% of dog ancestry, respectively; see Supplementary Results, p. 2). The mean estimated dog ancestry across all autosomal chromosomes was 3%. Chromosome 23 had only 261 SNP loci retained for the Lamp analysis (based on the LD threshold r^2^ < 0.1), while the range of loci retained in the remaining autosomal chromosomes was from 1701 (in chromosome 1) to 501 (in chromosome 38). The ancestry inference at chromosome 23 could be thus affected by the reduced number of informative SNP loci.

The Lamp analysis of wolf-golden jackal admixture showed that modern wolves carry no more than 0.2% of chromosomal blocks originating from recent admixture with golden jackals, and jackals – with one exception – carry 0.1–0.3% of chromosomal blocks originating from recent admixture with wolves (Table 1, Supplementary Table 5, Supplementary Fig. 11). One golden jackal from the Caucasus had 1.7% of wolf ancestry across autosomal chromosomes, and displayed 3–21% of wolf ancestry in 7 chromosomes. The Taimyr wolf had 2% of estimated golden jackal ancestry across autosomal chromosomes, but this resulted from a large proportion (48%) of putative golden jackal ancestry inferred in one chromosome only - chromosome 37. This chromosome had a relatively small number of SNP loci retained for the Lamp analysis (510), but this number was typical of other chromosomes with similar length, as chromosomes 35–38 all had less than 600 loci retained for the analysis. Therefore, the golden jackal ancestry inferred for chromosome 37 cannot be solely attributed to the small number of SNP loci used in the analysis, but we cannot exlude the possibility that low resolution affected the Lamp inference.

### Admixture inferred from ancestry blocks analysis in Elai

The results of ancestry block analysis in Elai^35^ (assuming 100 generations of admixture) were consistent with Lamp results for the identification of wolf-dog hybrids and recent backcrosses (Supplementary Table 6, Supplementary Fig. 12). Elai results also supported Lamp results showing dog admixture in Eurasian wolf populations, but no dog admixture (with few exceptions) in North American wolves except Mexican wolves. Elai identified higher admixture levels than Lamp in several breeds that experienced historical hybridisation, including Saarloos and Czechoslovakian wolfdogs, East Asian and Arctic breeds, Eurasier, and Alaskan husky (Supplementary Table 6).

## Discussion

The main phylogeographic split among the grey wolf populations studied occurred between Eurasian and North American populations, consistent with their long-term separation since the post-glacial flooding of the Bering Land Bridge. In Eurasia, the main phylogeographic split occurs between East Asian and West Eurasian wolves, although their distinctiveness could partially result from discontinuities in our geographic sampling coverage. Among North American wolves, the most distinct populations were Mexican wolves and Great Lakes wolves (represented here by Minnesota wolves). The distinctiveness of Mexican wolves likely results from geographic isolation and a demographic bottleneck^26,36^. The distinctiveness of the Great Lakes wolves has been explained either by considerable coyote admixture^26–28,37^ or by a distinct evolutionary origin^38,39^ (see the Supplementary Material for a more comprehensive reference list). Our study does not have comprehensive sample coverage from the Great Lakes wolves and therefore we cannot make conclusions regarding their taxonomic status.

Our TreeMix analysis showed that when the possibility of gene flow between coyotes and wolves is considered, all North American wolves, including Mexican and Minnesota wolves, form a single cluster. The monophyly of all North American wolves is consistent with an inference based on whole nuclear genomes^28^. A study based on the mitochondrial genomes also concluded that North American wolves originated from a single colonisation event from Asia that took place 23–30 Kya, after the formation of the Bering Land Bridge^18^. None of the Eurasian wolf populations appears to be genetically closer to North American wolves than any other populations. This is consistent with the inference from whole nuclear genome data, showing that all modern Eurasian wolf populations had a common ancestor about 30 Ky ago, and diversified only after divergence from North American wolves^15^.

Following a range expansion event, linkage disequilibrium in populations is expected to increase with distance from the geographic centre of expansion^40^. All North American wolf populations had higher LD levels than Eurasian wolves, and showed an increase in LD from north (Alaska) to south (New Mexico), consistent with a single expansion event from Asia to North America through Beringia. Alternatively, the increase in LD can be explained by recent wolf population declines in the contiguous USA, which were especially severe in the Mexican wolves. However, our results are consistent with other lines of evidence that suggest a single southward expansion in North America^18^. In contrast, the LD patterns in Eurasian populations did not clearly point to a serial founder model of expansion out of Beringia to Eurasia. Although a single geographic gradient of LD would be expected in the case of a single expansion event out of Beringia proposed in^19^, the LD patterns resulting from a population expansion could have been modified by subsequent events (such as hybridisation events and recent diversity loss due to extensive hunting), concealing the geographic expansion origin.

The ancient Taimyr wolf did not clearly assign to modern Siberian wolves or any other regional wolf population. Our TreeMix analysis placed the Taimyr wolf as the sister lineage to modern Eurasian wolves and domestic dogs, consistent with the original inference^10^. Accordingly, our inference of population genetic structure did not reveal clustering of the Taimyr wolf with any modern wolf population, demonstrating its distinctiveness. It should be noted however that estimates obtained for a single ancient individual are unlikely to be representative of the whole population from which it originated. Therefore, our results regarding the Taimyr wolf should be interpreted cautiously. Nevertheless, they highlight the importance of carrying out similar analyses for a larger number of Pleistocene wolves once their genotypes become available.

Ambiguity in the inferred position of the Taimyr wolf lineage relative to modern North American wolves may result from admixture between North American wolves and coyotes. Assuming purely dichotomous lineage splits in the presence of post-split gene flow can lead to biased reconstruction of the evolutionary history of the lineages studied. However, the ambiguity in Taimyr wolf position may also suggest that both these lineages diverged from modern Eurasian wolves at a similar time. The demographic estimate of coalescence time for all modern wolves based on whole genome sequence data is about 32.5 Kya^15^, making the Taimyr wolf nearly contemporary with the divergence time between modern Eurasian and North American wolves. The beginnings of dog domestication were estimated at 33–29 Kya^15,41^, shortly after the divergence between North American and Eurasian wolves^15^. This suggests that diversification among the main contemporary wolf lineages, the Taimyr lineage, and the domestic dog lineage occurred within a short time frame close to the onset of the Last Glacial Maximum (33–26.5 Kya^42^).

During that period, wolves experienced considerable demographic declines and phylogeographic shifts^14,15,19^, with disappearance of distinct ecomorphs, likely due to declines and extinctions of their prey species^16,43^. Changes in density and species composition of ungulate communities could have led to local extinctions, but also diversification of lineages as they were shifting their dietary niches in new directions, including that of human commensal. The ecological and phylogeographic changes occurring in that period have played a crucial role in the evolutionary history of the grey wolf and domestic dog.

Our results show that the evolution of the genus *Canis* was substantially affected by hybridisation. The exact admixture proportions inferred for regional populations are prone to lack of accuracy, given that the inference models are inherently simpler than the actual evolutionary history of these populations. Despite this limitation, the admixture models are a useful tool to infer the effect of gene flow events on the evolutionary trajectories of populations. Admixture analysis indicated that contemporary Asian wolves have positive, but small, assignment values to the cluster composed of golden jackals, black backed jackals and coyotes (which were clustered together due to small sample sizes; see Supplementary Fig. 5). Only the golden jackal could be involved in admixture with Asian wolves given that the two other species have never occurred in Eurasia. Admixture proportions between modern wolves and golden jackals inferred in Lamp were smaller compared to the results obtained from Admixture and TreeMix, probably because the Lamp inference was restricted to the last 10 generations. The Admixture and TreeMix results are thus likely reflecting hybridisation events in a more distant past. The inference of ancient admixture between wolves and golden jackals is consistent with earlier results based on whole genome sequence data^15,23,25^. This does not exclude the possibility of more recent gene flow events between these two species, which have also been reported before^24,44^. Distinguishing between the ancient and recent admixture requires a larger number of wolf and golden jackal samples from the regions where their ranges overlap, as well as higher density of SNP loci.

The TreeMix analysis inferred multiple cases of gene flow between wolves and dogs, consistent with earlier studies, e.g.^30,45–48^. Accordingly, the ancestry blocks analysis implemented in Lamp and Elai identified signatures of dog admixture in all Eurasian wolf populations and in Mexican wolves. The Mexican wolf population was the only North American population displaying dog admixture (not counting single admixed individuals detected in some other populations). Because this population is small and highly inbred^36^, dog-derived alleles could have drifted to a relatively high frequency in this population following a single hybridisation event. Alternatively, this result could be due to high frequency of alleles representing shared ancestral polymorphisms between wolves and dogs.

Both Lamp and Elai inferred limited wolf admixture in dog breeds of European origin and European free-ranging dogs, but higher levels of wolf admixture in free ranging and pure-bred dogs from East Asia and the Arctic, consistent with inference from our earlier study based on an independent dataset^48^. Admixture in the Neolithic Irish dog^49^ was low and within the range for modern European dogs. We also inferred gene flow from the ancient Taimyr wolf lineage to the ancestral population of Arctic breeds, consistent with Skoglund *et al*.^10^. Given the distinctiveness of the Taimyr lineage from modern wolves, this implies independent wolf introgression events in the Artic dogs occurring at multiple time periods.

The Taimyr wolf is expected to be free from dog admixture, as it was dated at 35 Kya - the time that either precedes or is contemporary with the beginnings of the dog domestication process^10^. The pattern inferred from the ancestry block analysis, with 36 chromosomes having entirely wolf ancestry, and a high proportion of dog ancestry in two remaining chromosomes, is inconsistent with the neutral introgression of hybridisation-derived alleles. Our results also indicate that the Taimyr wolf lineage received gene flow from coyotes and/or golden jackals. However, Taimyr Peninsula’s northern location precludes the presence of golden jackals during the Late Pleistocene, and the coyote has never occurred in Eurasia, making a local hybridisation event unlikely. The ancestry block analysis showed no evidence of golden jackal ancestry in the Taimyr wolf except in one chromosome, having almost 50% of inferred jackal ancestry. Again, such pattern could not have resulted from neutral introgression. It can be attributed to low number of informative SNP loci at the chromosomes where the exogenous ancestry was inferred, combined with the presence of shared ancestral variation between the lineages. This suggests that errors in the admixture inference resulting from limited genetic information used in the analysis are unlikely to produce consistent admixture patterns across multiple chromosomes. Therefore, analysis of ancestry blocks within chromosomes may provide an efficient way of identifying errors in the genome-average admixture inference.

The analysis of canid admixture should be ideally based on whole-genome sequence data in order to maximise the number of variable sites used. However, the cost of high-coverage whole-genome sequencing is still relatively high, limiting the number of individuals that can be analysed (e.g. Fan *et al*.^15^ included 34 individuals, and Sinding *et al*.^28^ included 40 individuals). Our study showed large variation in admixture proportions within populations, implying that admixture inference for single individuals is not representative of population-wide patterns. Genome-wide SNP genotyping allows the generation of data for a large number of individuals and provides sufficient density of variable sites to carry out the ancestry block analysis. Applying SNP chips to species other than the one used to design the chip carries the unavoidable potential for ascertainment bias. Although we cannot exclude that this is affecting our results, our analyses suggest the bias is likely low (see Supplementary Information for more detailed discussion of ascertainment bias). Therefore, genome-wide SNP genotyping is currently the optimum method to generate the population-level data required for admixture estimates, and it can complement the studies based on whole-genome sequence data.

## Conclusions

Global phylogeographic patterns in modern wolf populations have been shaped by the major phylogeographic shift occurring close to the end of the Pleistocene and subsequent local admixture events. Eurasian wolf populations carry signatures of geographically widespread admixture with dogs and rare admixture with golden jackals, while North American wolf populations display varying levels of admixture with coyotes and rare admixture with dogs. Introgressive hybridisation from ecologically diverse con-specific and con-generic populations can be maladaptive in many circumstances. However, in periods of rapid environmental change such introgression could have facilitated wolf adaptation to rapidly changing environmental conditions. This was also suggested in the case of brown bear (*Ursus arctos*) and polar bear (*Ursus maritimus*) admixture^50^. Admixture affected the inferred relationships between regional wolf populations, stressing the importance of accounting for this process in phylogeographic studies, if closely related species are or were present within the study region.

Both linkage disequilibrium patterns and reconstruction of ancestry relationships suggest that North American grey wolves (including the Mexican wolves) originated from a single colonisation event from Asia, consistent with results of a recent study based on whole genome data^28^. However, there is no clear evidence for a serial founder model of wolf expansion out of Beringia into Eurasia. Wolves from central-east Asia had lowest linkage disequilibrium of all populations studied, and other Eurasian populations did not show a linear geographical gradient of linkage disequilibrium, implying a complex evolutionary history.

Consistently, we found that the ancient Taimyr wolf lineage does not clearly assign to modern Siberian wolves or any other regional wolf population. The genetic legacy of this lineage has persisted in Artic dog breeds as a result of ancient admixture, but was diluted by subsequent gene flow from modern wolves. The reconstruction of ancestry relationships among wolf populations placed the ancient Taimyr wolf as a sister lineage of modern Eurasian wolves and domestic dogs, with an ambiguous position relative to North American wolves. This ambiguity suggests that the divergence of lineages leading to modern Eurasian and North American wolves, the Taimyr wolf, and domestic dogs, occurred within a short period, and was probably triggered by environmental changes associated with the onset of the Last Glacial Maximum. Evolutionary processes occurring in that period shaped the genetic diversity patterns in extant wolves and early domestic dogs.

The evolutionary history of another large Holarctic carnivore, the brown bear, follows a similar pattern of global lineage replacement^4^ and widespread introgressive hybridisation from closely related species^11,50^. The observed patterns may therefore represent a common evolutionary response of megafaunal species to environmental changes during the Pleistocene/Holocene transition, which warrants a comparative study including multiple extant and extinct species.

## Materials and Methods

### Dataset

Our dataset consisted of grey wolves *Canis lupus* representing the lineages geographically widespread across Eurasia and North America, and excluded genetically distinct taxa such as the African golden wolf and the Himalayan wolf, which are considered as either subspecies of *Canis lupus* or distinct species *Canis anthus*^44^ and *Canis himalayensis*^51^, respectively.

Using the CanineHD BeadChip (Illumina), we genotyped 84 grey wolves from different parts of Asia: Georgia, the Caucasus (11 individuals), Nagorno-Karabakh, the Caucasus (18 individuals), Mongolia (14 individuals), Russian Yakutia-Sakha Republic (38 individuals) and Saudi Arabia (two individuals). Two additional individuals (Mongolia and Saudi Arabia) sampled as grey wolves were genetically identified as domestic dogs, and one individual from Nagorno-Karabakh was identified as a golden jackal. These misidentified individuals were included in the dog and jackal datasets. Modern wolves were compared with the grey wolf from Taimyr in Siberia dated at 34.9 Kya^10^. We used the SNP genotypes that were extracted from the whole-genome sequence data in Skoglund *et al*.^10^ to allow comparison with the dataset of pure-bred dogs from Vaysse *et al*.^52^. In addition, we genotyped samples of 44 modern grey wolves from Bulgaria, 7 Eurasian golden jackals (*Canis aureus;* 3 from Bulgaria and 4 from the Caucasus), and 3 black backed jackals (*Canis mesomelas*) from Namibia. All these samples were genotyped at 167,989 autosomal SNP loci and 5,660 X-chromosome SNP loci.

We also included samples from other published studies, all of which were genotyped using the CanineHD BeadChip:

- European and North American wolves from Vaysse *et al*.^52^;
- European wolves from Stronen *et al*.^53^;
- North American wolves, mix-breed dogs and coyotes (*Canis latrans*) from Cronin *et al*.^54^;
- Mexican wolves from Fitak *et al*.^55^;
- pure-breed domestic dogs from Vaysse *et al*.^52^;
- free-ranging Eurasian dogs and pure-breed dogs from Pilot *et al*.^30^;
- free-ranging dogs, Asian dog breeds and one Neolithic domestic dog from Ireland dated at 4.8 Kya from Frantz *et al*.^49^;
- Alaskan huskies from Vernau *et al*.^56^, included in the Fitak *et al*.^55^ dataset;

Because small sample sizes of the two jackal species biased the results of population structure analysis, we later produced additional data for 94 golden jackals from nine European countries and the Caucasus, and 6 black backed jackals from Namibia and included them in selected analyses. Geographic distribution of wild and free-ranging canids studied is presented in Fig. 1, and Supplementary Table 1 contains the list of all samples.

All datasets included in the analyses were generated using CanineHD BeadChip, which facilitated their merging. Merging independent datasets may potentially be compromised by incompatibilities between genotypes from different datasets (batch effect). However, most of the databases listed above were generated using the “TOP/BOT” SNP calling method, which was designed to ensure uniform reporting of strand designation and orientation in different datasets (Illumina, Inc. 2006). The datasets that were reported using the “forward” SNP calling method were modified to fit the remaining datasets by flipping the mismatching loci based on the list provided by Illumina. We observed that individuals originating from different datasets but representing the same dog breeds or wolf populations were clustered together in Admixture and PCA analyses, which demonstrates correct merging of the datasets (Supplementary Table 8). Some of the published datasets reported a reduced set of SNP loci compared with the total number included in the SNP chip used, and did not report X chromosome loci. Therefore, the merged dataset included the data for 106,549 autosomal SNP loci. We removed from this dataset all individuals with more than 20% of missing data except the Taimyr wolf.

### Ethical approval

The study obtained an approval from the Ethics Committee of the College of Science, University of Lincoln (CoSREC365). Tissue samples used in this study were obtained from individuals that were killed as a result of legal hunting or in vehicle collisions. No animal was killed for the purpose of this research. All methods were performed in accordance with the guidelines of the American Society of Mammalogists on the use of wild animals in research^57^ and with all guidelines and regulations on sample collection from wild-living animals in the countries where the samples were collected and analysed.

### Estimation of population heterozygosity and linkage disequilibrium

We used the dataset described above to calculate observed and expected heterozygosity in regional wolf populations, using Plink^58^. We used the same software to assess the linkage disequilibrium in regional wolf populations by calculating genome-wide pairwise genotypic association coefficient r^2^ between alleles of autosomal SNP loci with MAF > 0.15 and containing no more than 10% of missing data. Because linkage disequilibrium estimates are dependent on sample sizes^59^, we carried out the calculations for 10 unrelated individuals per population, randomly selected if the overall sample size was larger. Relatedness among individuals was assessed based on IBS (identity by state) distances calculated in Plink. We excluded the Saudi Arabia population from these calculations because of too small sample size.

### Analysis of population structure

In the merged dataset, there were large differences in sample sizes between populations, which could bias the inference of population genetic structure^60^. As recommended by Meirmans^61^, we reduced the sample sizes of larger populations by removing individuals with more than 20% of missing data and then randomly removing excess individuals in order to obtain comparable sample sizes of different wolf and dog populations, as well as similar total number of wolves and dogs. The final dataset included 697 individuals: 83 Asian, 108 European and 119 North American wolves, 160 free-ranging dogs, 180 pure-bred dogs, the Neolithic Irish dog, three Czechoslovakian wolfdogs, two Saarloos wolfdogs, 29 coyotes, eight golden jackals and three black backed jackals. The Taimyr wolf was kept in the dataset despite containing 57% of missing data (see below).

Prior to the analysis of population structure, we pruned the dataset from loci in high linkage disequilibrium. This was achieved by removing loci having alleles with genome-wide pairwise genotypic association coefficient r^2^ ≥ 0.5, calculated in Plink within 50 SNP sliding windows, with a step size of 10 SNPs. We also pruned invariant loci and those with very low minor allele frequency (MAF) (<0.01), as well as loci with more than 10% of missing data. This resulted in a dataset of 90,554 loci. The pruning was carried out based on variation in the entire dataset.

The whole-genome sequence of the ancient Taimyr wolf had low coverage (1.03x), but the SNP genotyping was performed only for loci that had the minimum Phred Quality Score of 30^10^, which corresponds to 99.9% of base call accuracy. As a result, reliable genotypes were available for 41.9% of the total number of 8,686,809 SNP loci known for dogs, and for 45,735 (42.9%) of SNP loci out of 106,549 total loci in the merged dataset.

A high percentage of missing data can affect the estimates of genetic differentiation of an individual from populations it is compared with. Therefore, we created a new dataset including only loci that contained data for the Taimyr wolf, had MAF > 0.01 and were free of strong LD (r^2^ ≤ 0.5). This resulted in a dataset of 42,320 loci.

Both datasets were analysed using the maximum likelihood clustering approach implemented in Admixture^31^. Admixture analysis was run for K values (the number of clusters) ranging from 1 to 10, using the default run termination criterion, which stops iterations when the increase in the log-likelihood between iterations is less than 10^−4^. The optimal K was identified as the value associated with the lowest cross-validation error compared to other K values. This error was assessed in a cross-validation procedure^31^, where all the observed genotypes (loci) were divided into ten partitions of roughly equal size, then all genotypes within each partition was converted to missing iteratively, and runs were repeated for the reduced datasets.

We also carried out a principal component analysis (PCA) using the package Smartpca from the software Eigensoft^62^ to visualize the dominant components of variability in the dataset. The inferred relationships between the modern populations were consistent between the dataset of 90,554 loci and the reduced dataset of 42,320 loci, but the results for the Taimyr wolf differed considerably (see Results). Therefore, in all further analyses we used the dataset of 42,320 loci that did not contain any missing data for the Taimyr wolf.

### Analysis of ancestry relationships and admixture patterns

We reconstructed ancestry relationships among wolf populations and assessed the presence of gene flow from dogs using the software TreeMix^33^. The dataset used in this analysis consisted of wolves from Asia, Europe and North America, free-ranging dogs from the same continents, pure-breed dogs of European and East Asian origin, coyotes and golden jackals. Black backed jackals were used as outgroup.

The ancestry relationships were reconstructed assuming either no post-divergence gene flow among populations, or a defined number (either 5 or 10) migration edges (gene flow events) inferred according to the order of their intensity. The maximum inferred number of 10 migration edges resulted from a trade-off between the possibility of detecting gene flow events of moderate intensity and maintaining clarity of the resulting plot. The analysis was based on the LD-pruned dataset, and the trees were constructed using blocks of 100 SNP loci rather than individual loci, to further account for LD. Node support was assessed through 100 bootstrap replicates, generated by re-sampling blocks of 100 SNPs. We carried out a jackknife analysis to assess whether the inclusion of each migration edge to the model significantly improved the fit of this model to the data. Besides the analysis including the full set of populations, we carried out an analysis including only modern canids from Asia and the Taimyr wolf, to identify the gene flow events between their respective lineages.

### Ancestry blocks analysis using software LAMP

We used the software Lamp^34^ to assess the presence of chromosomal blocks originating from hybridisation in individual canids. We selected this software because it allows ancestry blocks estimation without defining *a priori* ancestral non-admixed populations. Because Eurasian wolf populations show evidence of past admixture with dogs^15,48^, and some free-ranging dog populations and ancient dog breeds show signatures of wolf admixture^12,30,46^, prior identification of non-admixed individuals is not possible. In Lamp, the identification of ancestral populations is integrated with the admixture analysis, and this unique approach made this software ideal for the analysis of our dataset.

The analysis of wolf-dog admixture was based on the dataset containing 461 wolf genotypes (all individuals having less than 20% of missing data), and 622 dog genotypes including all free-ranging dogs and one representative of each modern breed (to avoid overrepresentation of dog breeds). This dataset was filtered to include only those SNP loci that were genotyped for the Taimyr wolf, were variable in the dataset (MAF > 0.01) and with no more than 10% of missing data. In the Lamp analysis, this dataset was subsequently pruned for loci with alleles in strong linkage disequilibrium (r^2^ > 0.1).

The mixture proportion of 0.43:0.57 was determined based on the frequencies of wolf and dog genotypes. This ratio accommodated a conservative scenario where no individuals are admixed. We used a recombination rate of 1e-10, and fraction of overlap between adjacent windows (offset) of 0.2. We assumed 10 generations since admixture, because power to detect recent admixture was diminished when more distant admixture events were assumed (i.e. the assumption of 100 generations since admixture resulted in high admixture estimates for all individuals).

We also carried out an analysis of wolf - golden jackal admixture. For this purpose, we used all the genotypes of golden jackals (8 individuals) and Eurasian wolves (122 individuals) with the genotyping rate above 80%. We assumed a mixture proportion of 0.06:0.94, which was the proportion of jackals to wolves. The remaining parameters were the same as in the previous analysis.

### Ancestry blocks analysis using software ELAI

We carried out a further ancestry block analysis using the software Elai^35^. While Lamp assumes one admixture episode a specified number of generations before present, Elai implements a model assuming continuous admixture throughout multiple generations, which is more realistic for wolf-dog admixture. Elai does not require a recombination map, but instead it estimates local recombination rates as part of the ancestry analysis, ensuring high accuracy. Unlike Lamp, Elai does not require filtering loci for linkage disequilibrium. In addition, Elai accounts for the presence of population substructure within the admixing groups. For this analysis we assumed admixture between wolves and dogs, representing two main clusters, over 100 generations, and the presence of 10 lower-layer clusters (5 times the number of upper level clusters, as recommended in Guan^35^). Parameters of the hidden Markov model were used to estimate 20 expected maximisation steps. Non-admixed individuals had to be indentified *a priori*, which was done using the Lamp results described above. Individuals were classified as non-admixed based on estimated admixture proportions <0.001 for dogs and <0.01 for wolves; we used different thresholds in order to obtain similar, representative numbers of dogs (304) and wolves (334) classified as non-admixed. The thresholds are very low in both cases and therefore the accuracy of non-admixed individuals’ identification is not compromised for either dogs or wolves.

## Supplementary information

Supplementary Information

## Data Availability

The canid genotypes produced in this study and the associated geographic information can be accessed using the following link: https://data.mendeley.com/datasets/4k3yrn3brm/draft?a=1de061e5-6af3-4093-aabe-d45ea8a2dec4.
