# Supplementary material for: Global Phylogeographic and Admixture Patterns in Grey Wolves and Genetic Legacy of An Ancient Siberian Lineage
Source: Sci Rep. 2019 Nov 22;9:17328. doi: 10.1038/s41598-019-53492-9 (PMC6874602; doi:10.1038/s41598-019-53492-9)
Supplement: Supplementary file 1 — Supplementary Information [file 41598_2019_53492_MOESM1_ESM.pdf]

**Supplementary Information**

# Global Phylogeographic and Admixture Patterns in Grey Wolves and Genetic Legacy of an Ancient Siberian Lineage

Małgorzata Pilot, Andre E. Moura, Innokentiy M. Okhlopkov, Nikolay Mamaev, Abdulaziz N. Alagaili, Osama B. Mohammed, Eduard G. Yavruyan, Ninna H. Manaseryan, Vahram Hayrapetyan, Natia Kopaliani, Elena Tsingarska, Miha Krofel, Pontus Skoglund, Wiesław Bogdanowicz

Table of Contents:


| | |
|---|---|
| Supplementary Results | Page 2 |
| Supplementary Discussion | Pages 3-4 |
| Supplementary References | Page 5-6 |
| Supplementary Figure 1 | Page 7 |
| Supplementary Figure 2 | Page 8-9 |
| Supplementary Figure 3 | Page 10-11 |
| Supplementary Figure 4 | Page 12-13 |
| Supplementary Figure 5 | Page 14 |
| Supplementary Figure 6 | Page 15-16 |
| Supplementary Figure 7 | Page 17-18 |
| Supplementary Figure 8 | Page 19-20 |
| Supplementary Figure 9 | Page 21-22 |
| Supplementary Figure 10 | Page 23-24 |
| Supplementary Figure 11 | Page 25 |
| Supplementary Figure 12 | Page 26 |
| Supplementary Figure 13 | Page 27 |
| Supplementary Figure 14 | Page 28 |
| Supplementary Table 1 | Page 29-30 |
| Supplementary Table 2 | Page 31 |
| Supplementary Table 3 | Page 32 |
| Supplementary Table 4 | Page 33-34 |
| Supplementary Table 5 | Page 35 |
| Supplementary Table 6 | Page 36 |
| Supplementary Table 7 | Page 37 |
| Supplementary Table 8 | Page 38-64 |

**Supplementary Results - Ancestry analyses for chromosomes 23 and 37**

The LAMP analysis inferred dog ancestry in the Taimyr wolf for 22% of SNP loci in chromosome 15 and 100% of SNP loci in chromosome 23 (Supplementary Figure 10). Such pattern could not have resulted from neutral introgression of dog-derived alleles; moreover, the presence of the dog admixture in a wolf dated at 35 Kya is unlikely. The Taimyr wolf also showed a signature of golden jackal ancestry in only one chromosome (chromosome 37), where 48% of chromosomal blocks were assigned as originating from jackals (Supplementary Figure 11). This pattern was also inconsistent with the neutral introgression, and the occurrence of a hybridisation event was unlikely due to a large distance between the Taimyr Peninsula and the distribution range of golden jackals.

Because of the unexpected ancestry patterns observed for the Taimyr wolf in chromosomes 23 and 37, we carried out the ADMIXTURE and TREEMIX analyses for each of these chromosomes separately, using the same approach as in the respective analyses described in the main text. For chromosome 23, the ADMIXTURE analysis at K=3 assigned the Taimyr wolf to the dog cluster and wolf cluster with the proportions of 0.64 and 0.36, respectively, with no admixture from golden jackals detected (Supplementary Table 7). In the tree constructed in TREEMIX for this chromosome, the Taimyr wolf clustered with dogs with 100% bootstrap support, although its clustering with particular breeds or populations was not supported (Supplementary Figure 13). For chromosome 37, ADMIXTURE analysis at K=3 identified the clusters composed of (a) dogs, (b) Eurasian wolves and (c) coyotes, jackals, and North American wolves. The assignment proportions of the Taimyr wolf to these three clusters were 0.13, 0.29 and 0.58, respectively. In the tree constructed in TREEMIX for this chromosome, the Taimyr wolf was placed as a sister lineage of all modern wolves except Minnesota wolves (Supplementary Figure 14). Further investigation based on whole-genome sequence data is required to provide a correct interpretation of the distinct patterns observed in chromosomes 23 and 37.

**Supplementary Discussion - The effect of shared ancestral variation and ascertainment bias on the accuracy of admixture estimates**

This part of the discussion could not be included in the main text due to the manuscript length constraints.

The accuracy of admixture estimates may be affected by the presence of shared ancestral variation, leading to incorrect inference of hybridisation in the presence of population structure (Yang et al. 2012, Eriksson & Manica 2014). Our analysis represents an example of admixture inference in a structured population, given that the divergence of Eurasian and North American wolves preceded dog domestication (Fan et al. 2016). In principle, higher estimated dog admixture in Eurasian wolves compared with North American wolves could be explained by the common ancestry domestic dogs share only with Eurasian wolf populations, given that the domestication took place in Eurasia. However, the wolf population ancestral to dogs is likely extinct, given that dogs are sister group to all Eurasian wolves (Friedman et al. 2013, Fan et al. 2016). Furthermore, the divergence of Eurasian and North American wolves was estimated to occur only 285-1,565 years before the estimated divergence of dogs from Eurasian wolves (Fan et al. 2016) - almost simultaneously in the evolutionary time scale. Therefore, the amount of shared ancestral variation with dogs should be comparable between Eurasian and North American wolves.

If most of the detected admixture resulted from shared ancestral polymorphisms, we should expect random distribution of admixture proportions, but the observed distributions were highly non-random. For example, pure-bred dogs had – as expected – very low frequencies of inferred wolf admixture, wolf-dog breeds (Czechoslovakian wolfdog and Saarloos wolfdog) had high frequencies of wolf admixture, and among North American wolves one population (Mexican wolves) had high proportion of individuals with dog admixture, while other populations were nearly free of dog admixture. Therefore, it’s unlikely that shared ancestral variation considerably biased our results.

The accuracy of admixture estimates can also be affected by ascertainment bias, but relatively little is known about how this effect would be manifested in analytical tests. McTavish and Hillis (2015) showed that ascertainment bias can generate biases in PCA-based analyses of admixture. However, our admixture inference was not based on genome-average PCA analysis, but on the ancestry block analysis along individual chromosomes. This approach allowed us to correctly assign one copy of each chromosome to wolf and dog, respectively, in an F1 hybrid. If the ascertainment scheme or the presence of ancestral polymorphisms introduced strong bias in the ancestry inference, this would have resulted in deviations from the correct ancestry inference of 50%:50% at each autosomal chromosome.

Our results are consistent with studies that inferred admixture in genus *Canis* based on the analysis of whole genome sequence data, which are free of ascertainment bias (Fan et al. 2016, Sinding et al. 2018). Moreover, a large number of earlier studies – mostly based on the analysis of microsatellite loci – reported recent dog admixture in different Eurasian wolf populations (reviewed in Hindrikson et al. 2017), but such reports from North America are scarce (Munoz-Fuentes et al. 2010). The hybridisation patterns inferred from genetic data are also consistent with reports of wild wolf-like canids with morphological features unusual for wild grey wolves (e.g. Galaverni et al. 2018).

References cited

Eriksson, A., & Manica, A. (2014). The doubly conditioned frequency spectrum does not distinguish between ancient population structure and hybridization. *Molecular Biology & Evolution*, 31(6), 1618-1621. https://doi.org/10.1093/molbev/msu103

Fan, Z., et al. (2016). Worldwide patterns of genomic variation and admixture in gray wolves. *Genome Research*, 26, 163-173. https://doi.org/10.1101/gr.197517.115

Freedman, A. H., et al. (2014). Genome sequencing highlights the dynamic early history of dogs. *PLoS Genetics*, 10, e1004016. https://doi.org/10.1371/journal.pgen.1004016

Galaverni, M., Caniglia, R., Pagani, L., Fabbri, E., Boattini, A., & Randi, E. (2017). Disentangling timing of admixture, patterns of introgression, and phenotypic indicators in a hybridizing wolf population. *Molecular Biology & Evolution*, 34(9), 2324-2339. https://doi.org/10.1093/molbev/msx169

Hindrikson, M., et al. (2017). Wolf population genetics in Europe: A systematic review, meta-analysis and suggestions for conservation and management. Biological Reviews, 92(3), 1601–1629. https://doi.org/10.1111/brv.12298

McTavish, E. J., & Hillis, D. M. (2015). How do SNP ascertainment schemes and population demographics affect inferences about population history? *BMC Genomics*, 16(1), 266.

Munoz-Fuentes, V., Darimont, C. T., Paquet, P. C., & Leonard, J. A. (2010). The genetic legacy of extirpation and re-colonization in Vancouver Island wolves. Conservation Genetics, 11, 547–556. https://doi.org/10.1007/s10592-009-9974-1

Sinding, M. H. S., et al. (2018). Population genomics of grey wolves and wolf-like canids in North America. *PLoS Genetics*, 14(11), e1007745.

Yang, M. A., Malaspinas, A. S., Durand, E. Y., & Slatkin, M. (2012). Ancient structure in Africa unlikely to explain Neanderthal and non-African genetic similarity. *Molecular Biology & Evolution*, 29(10), 2987-2995. https://doi.org/10.1093/molbev/mss117

**Supplementary References - Publications on genetic differentiation and taxonomic status of canids from the Great Lakes region in North America**

The taxonomic status of Great Lakes wolves is the subject of a long-term controversy. Our study is not focused on this topic, but we refer to it while discussing our results. To avoid bias in referencing the studies that address this question using genetic methods, we provide here an extensive list of references obtained via an automatic search of Web of Science. We used two sets of keywords to carry out the search: (1) "*Canis*" AND "Great Lakes" and "genetic" and (2) "Great Lakes wolf" AND "genetic" and combined the resulting reference lists. We then checked the titles and abstracts for relevance, and removed three papers that were not focused on North American canids: two were focused on canids from other continents (Himalayan wolves and Australian dingoes) and one was focused on the lake sturgeon. The remaining papers are listed below.


Benson, JF; Patterson, BR; Wheeldon, TJ. 2012. Spatial genetic and morphologic structure of wolves and coyotes in relation to environmental heterogeneity in a Canis hybrid zone. MOLECULAR ECOLOGY 21: 5934-5954.

Bozarth, CA; Hailer, F; Rockwood, LL; Edwards, CW; Maldonado, JE 2011. Coyote colonization of northern Virginia and admixture with Great Lakes wolves. JOURNAL OF MAMMALOGY 92: 1070-1080.

Fain, SR; Straughan, DJ; Taylor, BF 2010. Genetic outcomes of wolf recovery in the western Great Lakes states. CONSERVATION GENETICS 11: 1747-1765.

Heppenheimer, E; Cosio, DS; Brzeski, KE; Caudill, D; Van Why, K; Chamberlain, MJ; Hinton, JW; vonHoldt, B. 2018. Demographic history influences spatial patterns of genetic diversity in recently expanded coyote (Canis latrans) populations. HEREDITY 120: 183-195.

Heppenheimer, E; Harrigan, RJ; Rutledge, LY; Koepfli, KP; DeCandia, AL; Brzeski, KE; Benson, JF; Wheeldon, T; Patterson, BR; Kays, R; Hohenlohe, PA; von Holdt, BM 2018. Population Genomic Analysis of North American Eastern Wolves (Canis lycaon) Supports Their Conservation Priority Status. GENES 9: 606.

Hohenlohe, PA; Rutledge, LY; Waits, LP; Andrews, KR; Adams, JR; Hinton, JW; Nowak, RM; Patterson, BR; Wydeven, AP; Wilson, PA; White, BN 2017. Comment on "Whole genome sequence analysis shows two endemic species of North American wolf are admixtures of the coyote and gray wolf" SCIENCE ADVANCES 3: e1602250.

Kays, R; Curtis, A; Kirchman, JJ 2010. Rapid adaptive evolution of northeastern coyotes via hybridization with wolves. BIOLOGY LETTERS 6: 89-93.

Koblmuller, S; Nord, M; Wayne, RK; Leonard, J 2009 REPLY - More is better. MOLECULAR ECOLOGY 18: 4994-4996.

Koblmuller, S; Nord, M; Wayne, RK; Leonard, JA 2009. Origin and status of the Great Lakes wolf. MOLECULAR ECOLOGY 18: 2313-2326.

Manlick, PJ; Romanski, MC; Pauli, JN 2018. Dynamic colonization history in a rediscovered Isle Royale carnivore. SCIENTIFIC REPORTS 8: 12711.

Mech, LD 2010. What is the taxonomic identity of Minnesota wolves? CANADIAN JOURNAL OF ZOOLOGY-REVUE CANADIENNE DE ZOOLOGIE 88: 129-138.

Mech, LD 2011. Minnesota Wolf Ear Lengths as Possible Indicators of Taxonomic Differences. NORTHEASTERN NATURALIST 18: 265-274.

Mech, LD 2011. Non-genetic Data Supporting Genetic Evidence for the Eastern Wolf. NORTHEASTERN NATURALIST 18: 521-526.

Nowak, RM 2002. The original status of wolves in eastern North America. SOUTHEASTERN NATURALIST 1: 95-130.
Power, JWB; LeBlanc, N; Bondrup-Nielsen, S; Boudreau, MJ; O'Brien, MS; Stewart, DT. 2015. Spatial Genetic and Body-Size Trends in Atlantic Canada *Canis latrans* (Coyote) Populations. NORTHEASTERN NATURALIST 22: 598-612
Randi, E 2010. Wolves in the Great Lakes region: a phylogeographic puzzle. MOLECULAR ECOLOGY 19: 4386-4388.
Rutledge, LY; Wilson, PJ; Klutsch, CFC; Patterson, BR; White, BN 2012. Conservation genomics in perspective: A holistic approach to understanding *Canis* evolution in North America. BIOLOGICAL CONSERVATION 155: 186-192.
Schwartz, MK; Vucetich, JA 2009. Molecules and beyond: assessing the distinctness of the Great Lakes wolf. MOLECULAR ECOLOGY 18: 2307-2309.
Sinding, MHS; Gopalakrishan, S; Vieira, FG; Castruita, JAS; Raundrup, K; Jorgensen, MPH; Meldgaard, M; Petersen, B; Sicheritz-Ponten, T; Mikkelsen, JB; Marquard-Petersen, U; Dietz, R; Sonne, C; Dalen, L; Bachmann, L; Wiig, O; Hansen, AJ; Gilbert, MTP 2018. Population genomics of grey wolves and wolf-like canids in North America. PLOS GENETICS 14: e1007745.
Stronen, AV; Paquet, PC 2013. Perspectives on the conservation of wild hybrids. BIOLOGICAL CONSERVATION 167: 390-395.
Stronen, AV; Tessier, N; Jolicoeur, H; Paquet, PC; Henault, M; Villemure, M; Patterson, BR; Sallows, T; Goulet, G; Lapointe, FJ. 2012. Canid hybridization: contemporary evolution in human-modified landscapes. ECOLOGY AND EVOLUTION 2: 2128-2140.
Thiel, RP. 2006. Conditions for sexual interactions between wild Grey Wolves*, Canis lupus*, and Coyotes, *Canis latrans*. CANADIAN FIELD-NATURALIST 120: 27-30.
vonHoldt, BM; Cahill, JA; Fan, ZX; Gronau, I; Robinson, J; Pollinger, JP; Shapiro, B; Wall, J; Wayne, RK 2016. Whole-genome sequence analysis shows that two endemic species of North American wolf are admixtures of the coyote and gray wolf. SCIENCE ADVANCES 2: e1501714.
vonHoldt, BM; Kays, R; Pollinger, JP; Wayne, RK 2016. Admixture mapping identifies introgressed genomic regions in North American canids. MOLECULAR ECOLOGY 25: 2443-2453.
vonHoldt, BM; Pollinger, JP; Earl, DA; Knowles, JC; Boyko, AR; Parker, H; Geffen, E; Pilot, M; Jedrzejewski, W; Jedrzejewska, B; Sidorovich, V; Greco, C; Randi, E; Musiani, M; Kays, R; Bustamante, CD; Ostrander, EA; Novembre, J; Wayne, RK 2011. A genome-wide perspective on the volutionary history of enigmatic wolf-like canids. GENOME RESEARCH 21: 1294-1305.
Wheeldon, T; White, BN2009. Genetic analysis of historic western Great Lakes region wolf samples reveals early Canis lupus/lycaon hybridization. BIOLOGY LETTERS 5: 101-104.
Wheeldon, TJ; Patterson, BR; White, BN 2010. Sympatric wolf and coyote populations of the western Great Lakes region are reproductively isolated. MOLECULAR ECOLOGY 19: 4428-4440.
Wheeldon, TJ; Rutledge, LY; Patterson, BR; White, BN; Wilson, PJ 2013 Y-chromosome evidence supports asymmetric dog introgression into eastern coyotes. ECOLOGY AND EVOLUTION 3: 3005-3020.
Wilson, PJ; Grewal, SK; Mallory, FF; White, BN. 2009. Genetic Characterization of Hybrid Wolves across Ontario. J. HEREDITY 100: S80-S89. 4th International Conference on Advances in Canine and Feline Genomics and Inherited Diseases, May 21-24, 2008. St Malo, Fr Polynesia, CNRS, Univ Rennes 1.

## SUPPLEMENTARY FIGURE 1

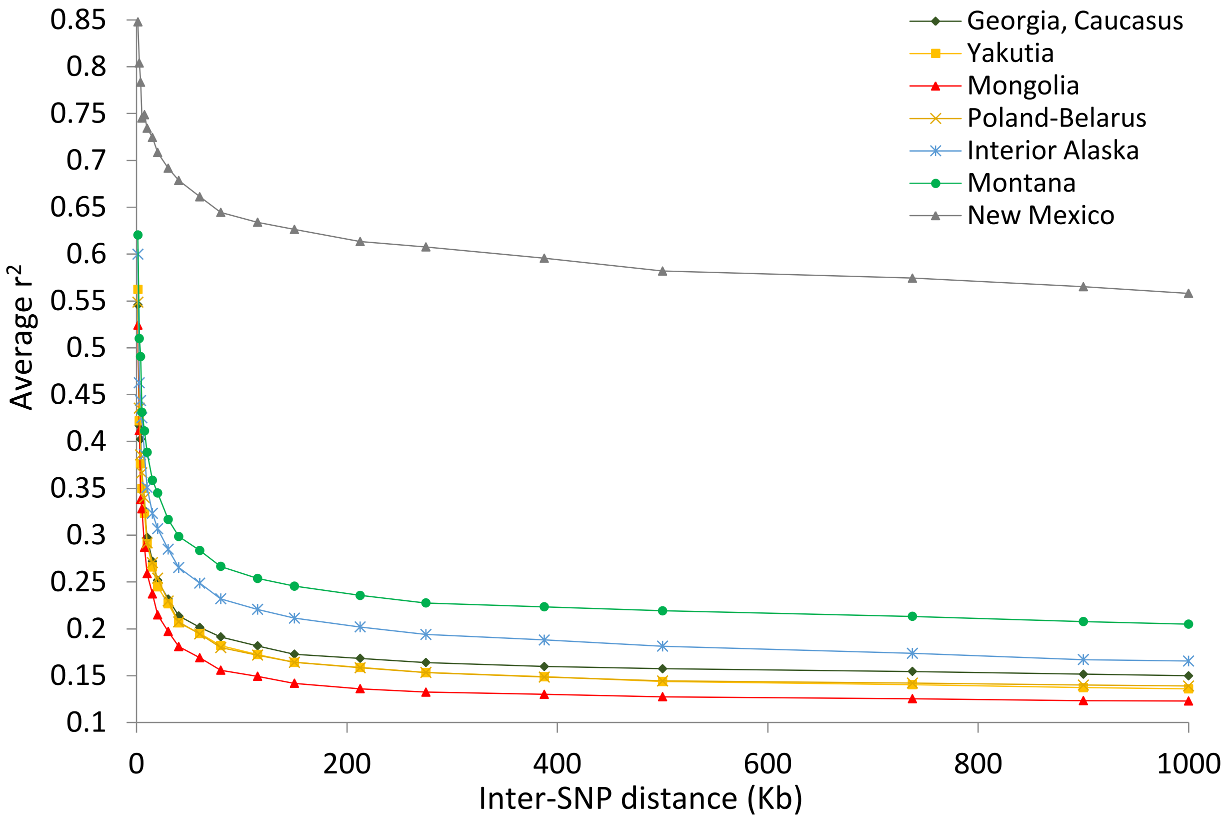


Supplementary Figure 1. Linkage disequilibrium patterns in wolf populations, including the Mexican wolves (New Mexico population). Linkage disequilibrium is represented by average genotypic association coefficient $r^2$ and is plotted as a function of inter-SNP distance. The estimate was carried out for 10 unrelated individuals per population.

## Supplementary Figure 2

**A**

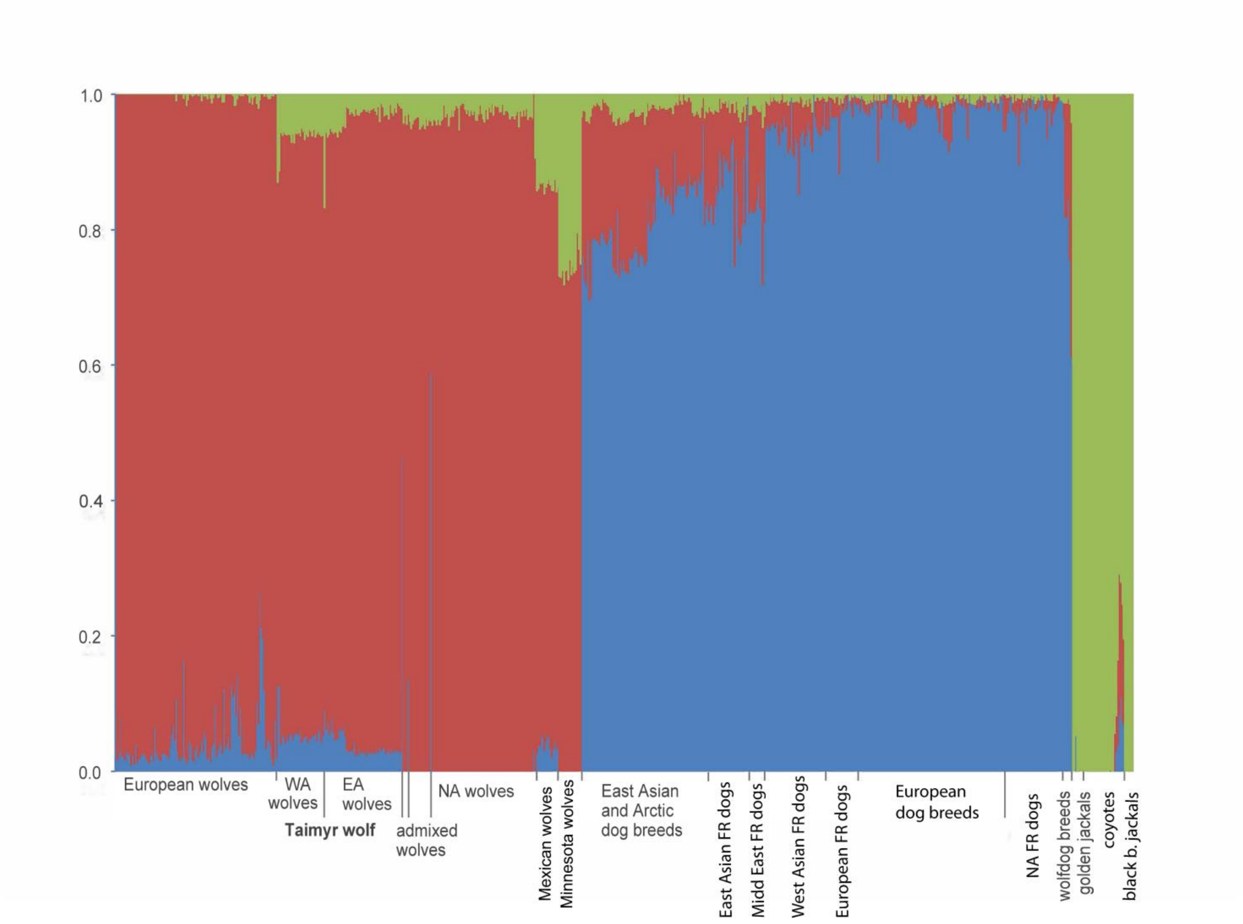

**B**

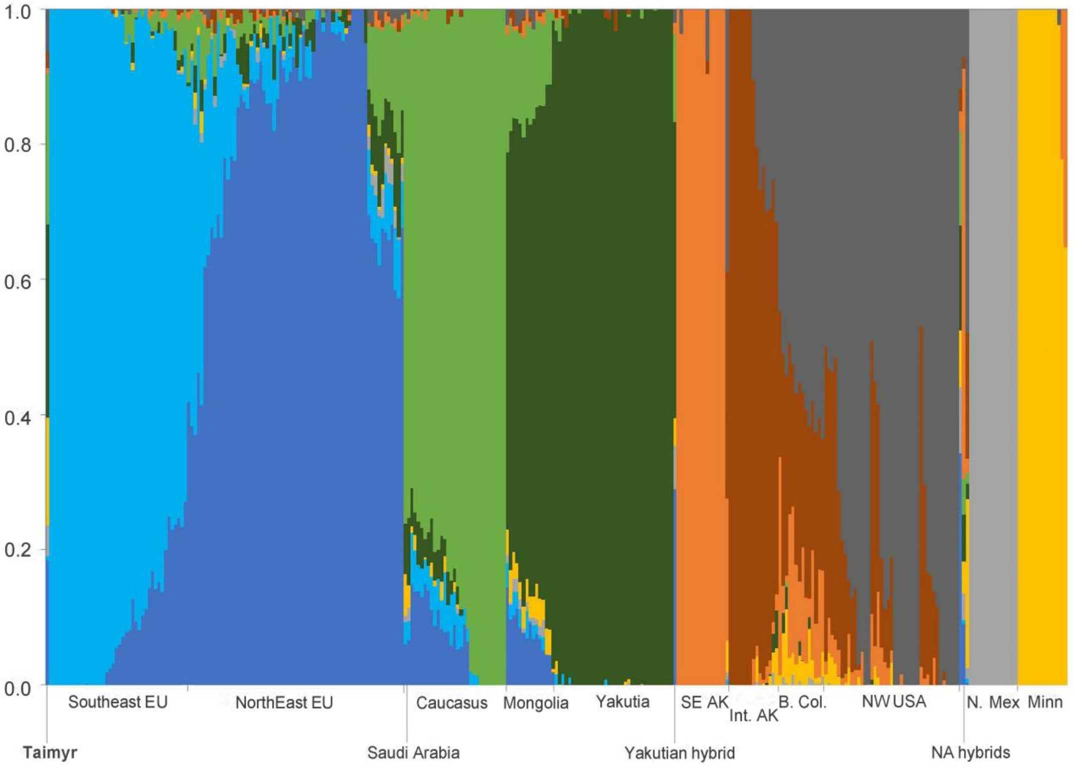


Supplementary Figure 2. Population structure inferred in Admixture for the dataset of (A) dogs, wolves and other wild canids, assuming three genetic clusters, (B) worldwide grey wolves assuming nine clusters. These figures are the same as Figures 3A and 3C, but provide more details. EU - Europe, NA - North America, AK - Alaska, B. Col - British Columbia, N. Mex - New Mexico, Minn - Minnesota. In Figure 2A, individuals labeled as “admixed wolves” are (in the order of listing): F1 black wolf-dog hybrid from Yakutia, back-crossed individual from British Columbia, and wolf-dog hybrid from British Columbia.

## Supplementary Figure 3

**A**

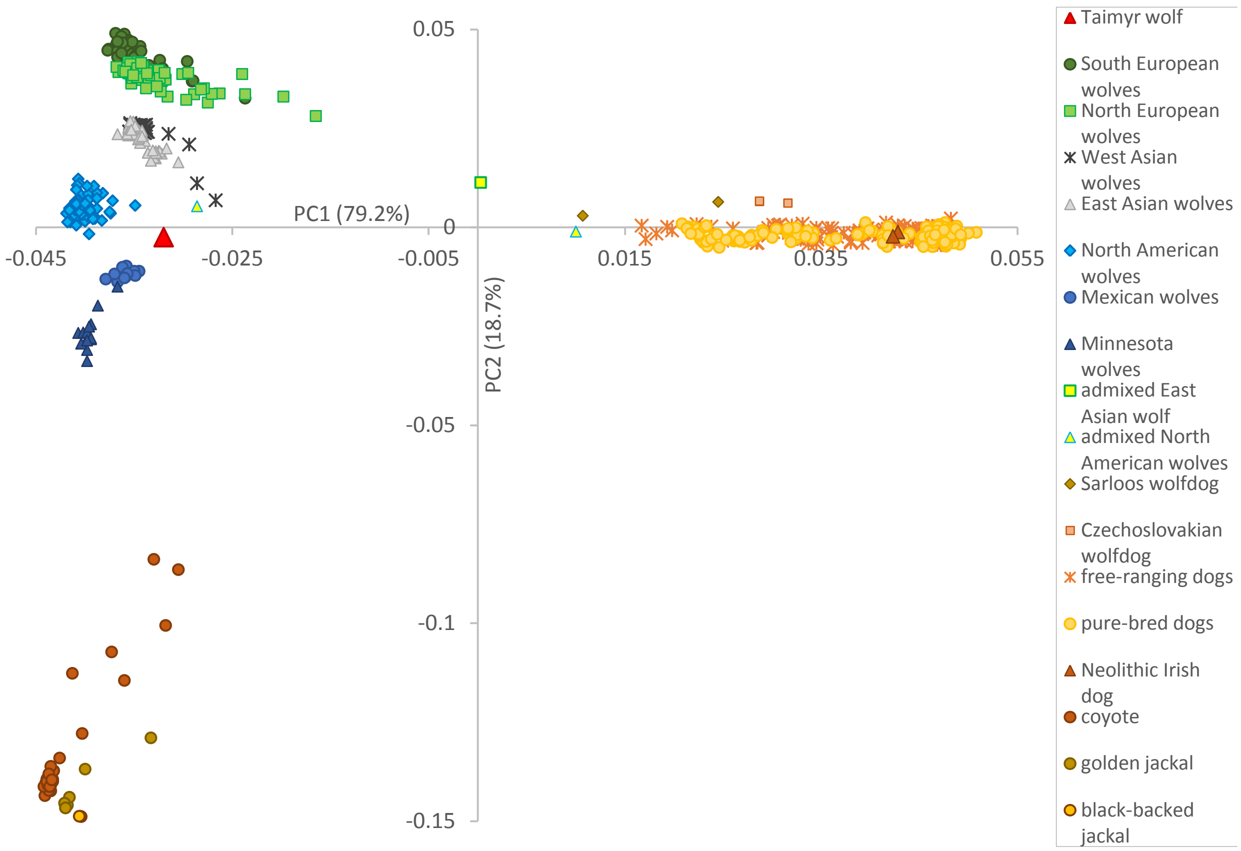

**B**

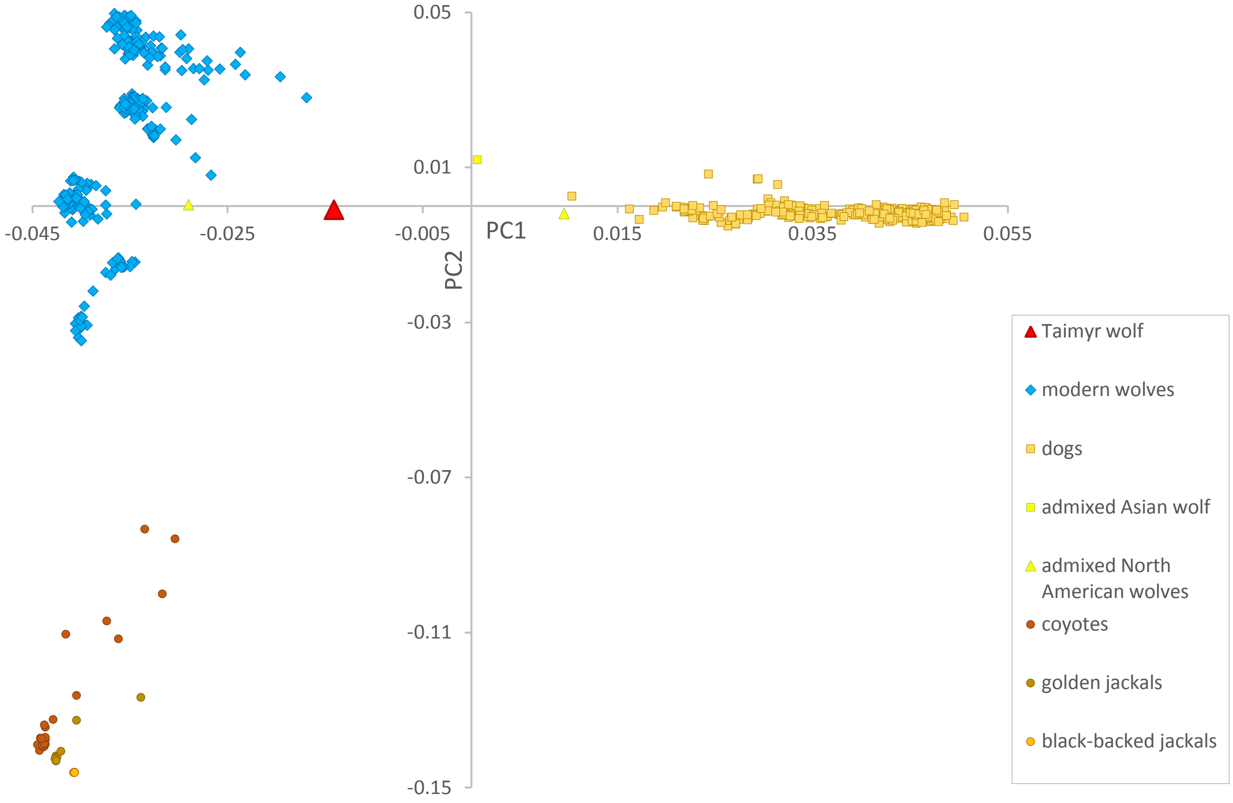


Supplementary Figure 3. PCA plot for a dataset of wolves, dogs and wild canids, with the number of samples reduced to ensure similar sizes of wolf and dog populations. (A) Based on the SNPs set was pruned to remove loci missing for the Taimyr wolf as well as those with strong LD, MAF<0.01 and more than 20% of missing data. This plot corresponds to Figure 3B, but shows more details. (B) Based on the SNPs set pruned to remove loci in strong LD, those with MAF<0.01 and more than 20% of missing data, and with the Taimyr wolf being included in the analysis despite having over 50% of missing data.

## SUPPLEMENTARY FIGURE 4

A

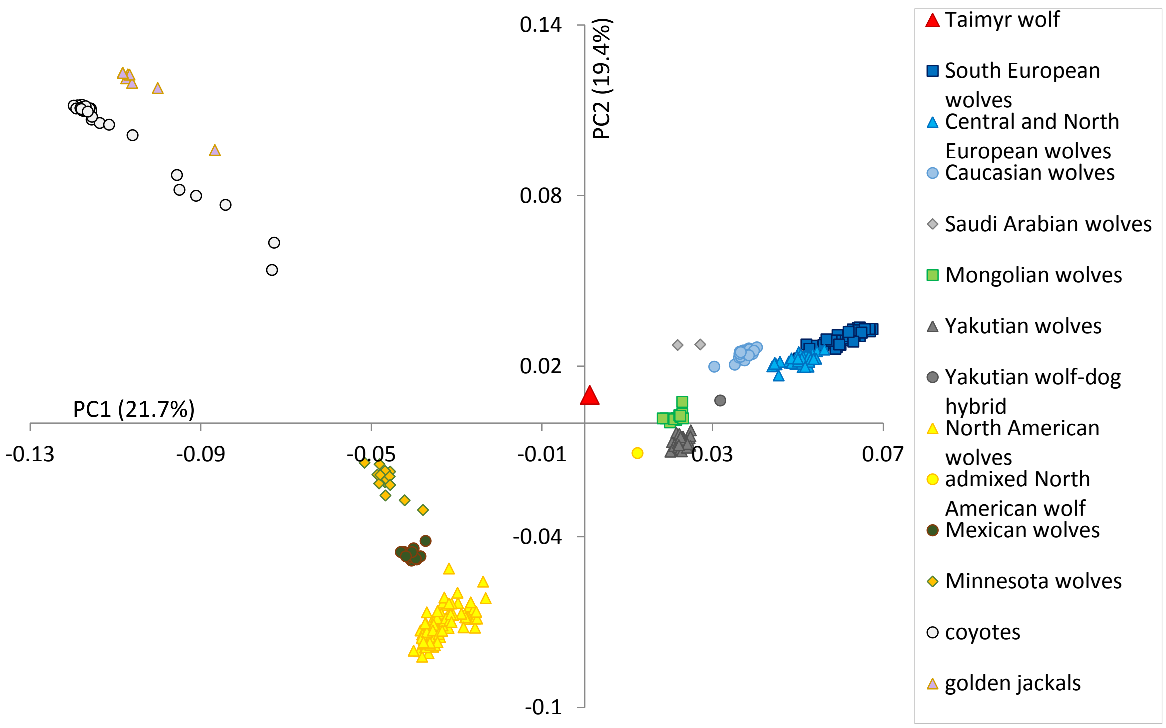


B

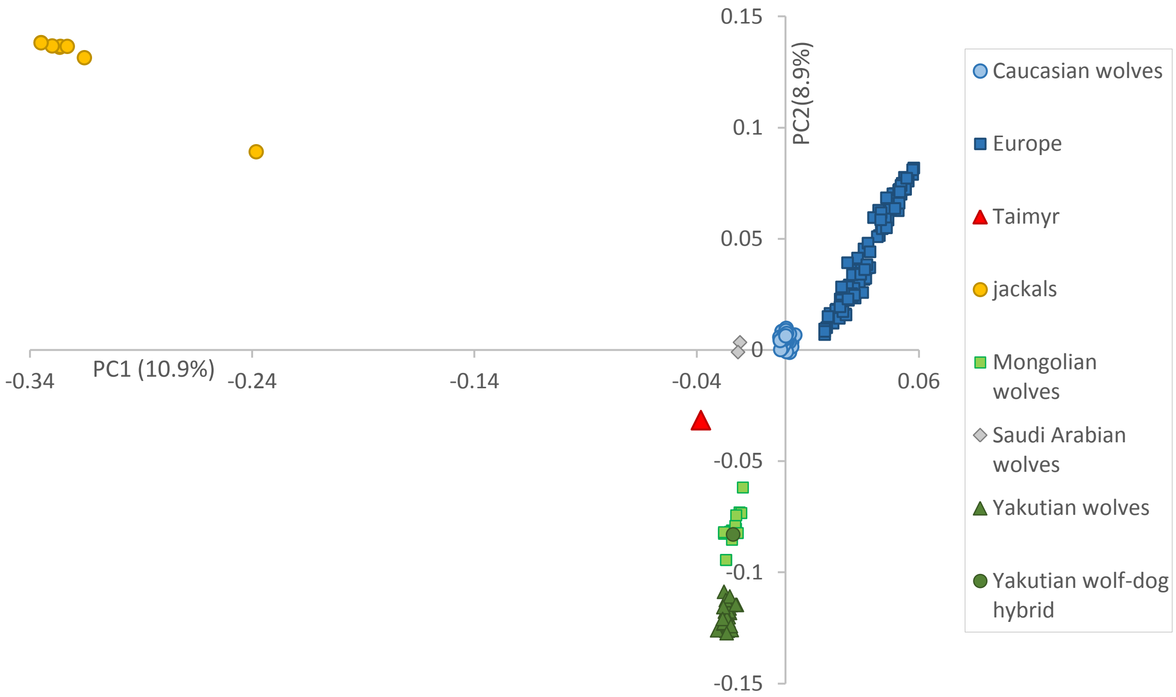


C

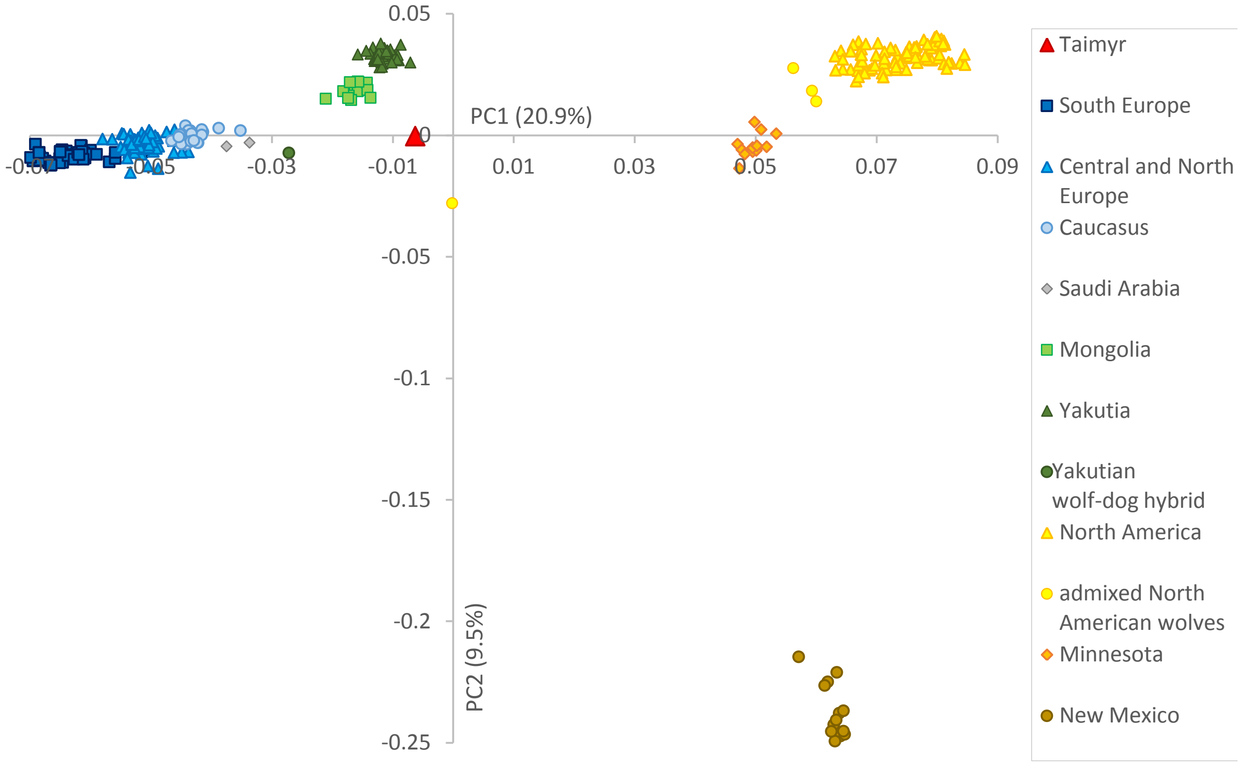


Supplementary Figure 4. PCA plot for a dataset of (A) wolves, golden jackals and coyotes, (B) Eurasian wolves and golden jackals, (C) worldwide wolves. The plot (C) corresponds to Figure 2C, but shows more details. The SNPs set was pruned to remove loci in strong LD, with over 10% missing data, MAF<0.01 and those missing for the Taimyr wolf.

## SUPPLEMENTARY FIGURE 5

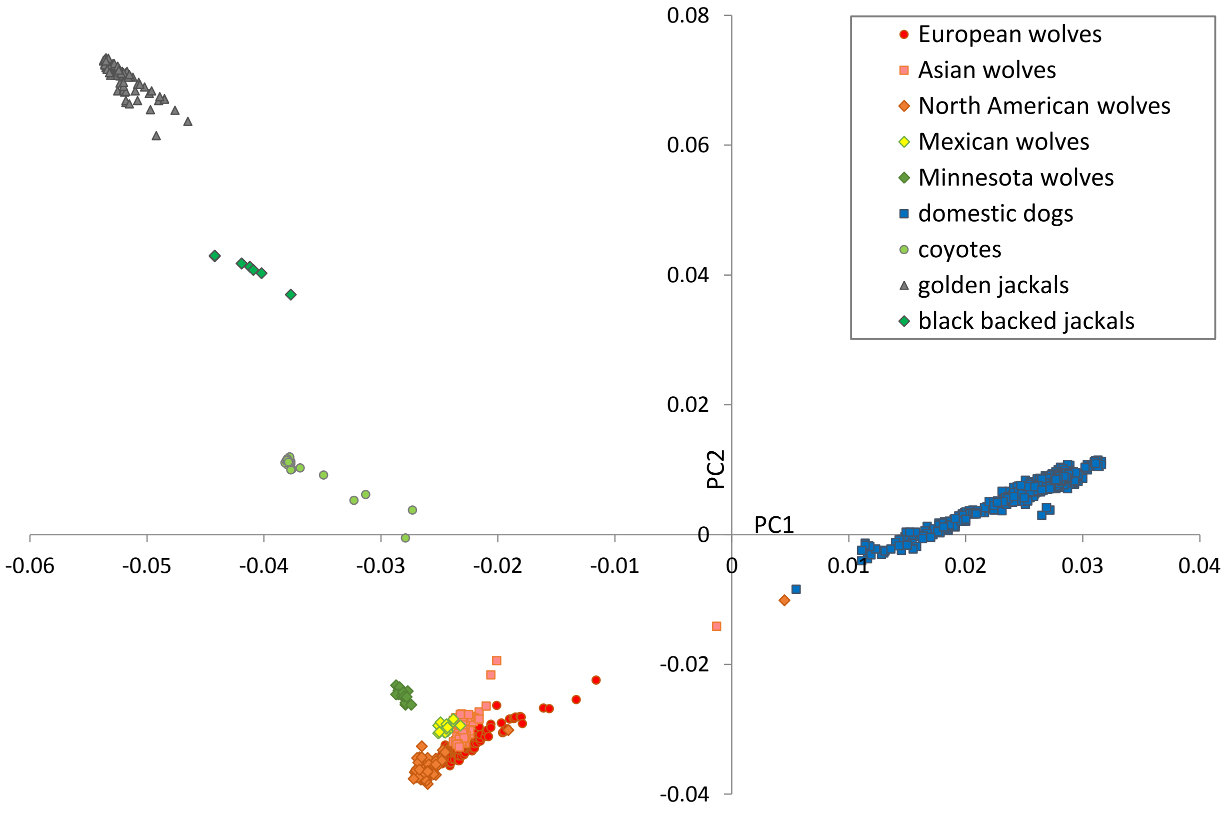


Supplementary Figure 5. PCA plot for a dataset of wolves, dogs and wild canids, including additional samples of 96 golden jackals and 6 black backed jackals that were not included in the PCA plots above. The plot was constructed for the SNPs set pruned to remove loci in strong LD, those with MAF<0.01 and more than 20% of missing data, and did not include the Taimyr wolf. In this plot coyotes, golden jackals and black backed jackals form three distinct clusters. This demonstrates that the reason why these species clustered together in the earlier analyses of population structure (Figure 3A,B, Supplementary Figures 2A, 3 and 4A) was the unbalance sample size for different populations rather than the ascertainment bias.

## Supplementary Figure 6

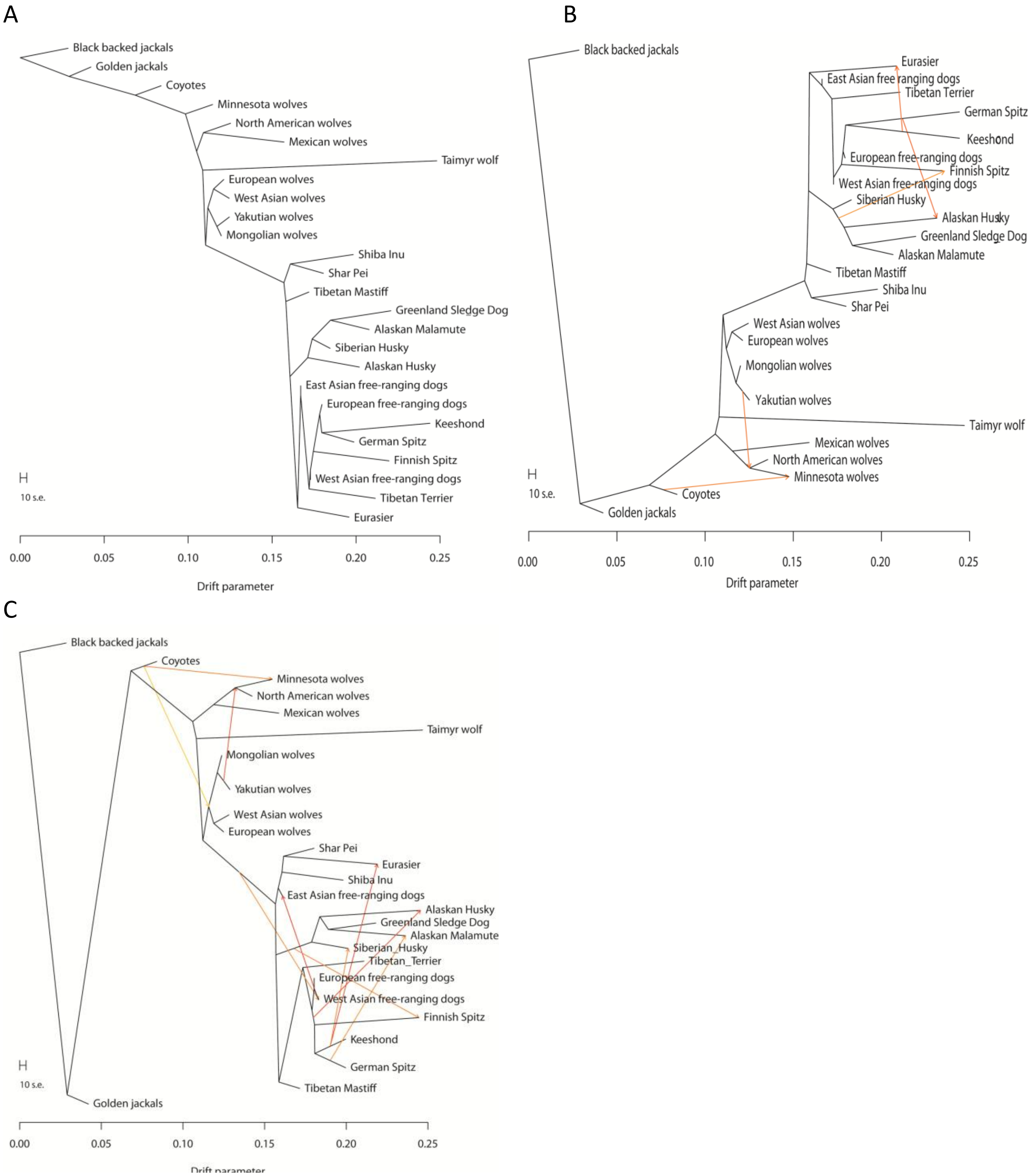

D

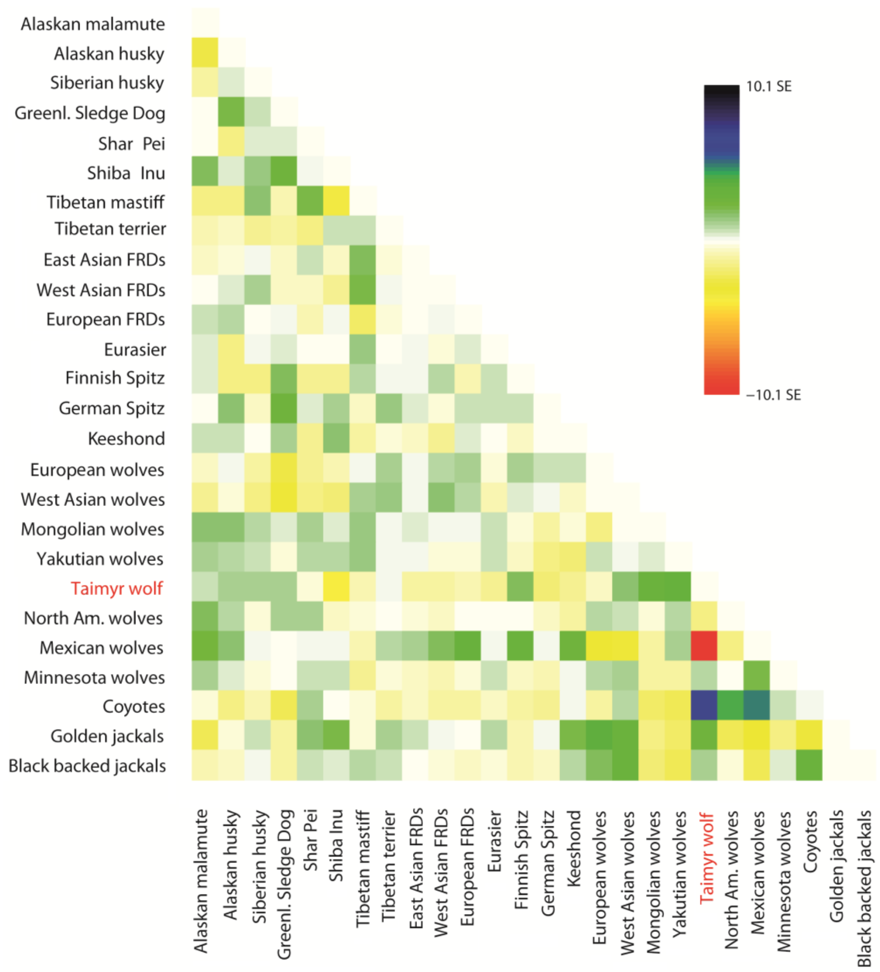


Supplementary Figure 6. Ancestry relationships between the Taimyr and modern wolf and dog populations inferred in TreeMix (A) without assuming gene flow, (B) assuming 5 events of gene flow. (C) assuming 10 events of gene flow. The colours of arrows reflect the intensity of gene flow, from lowest (yellow) to highest (red). (D) Plot of residuals for the phylogenetic reconstruction from figure 6C. "North American wolves" represent all North American populations studied except Minnesota wolves and Mexican wolves.

## SUPPLEMENTARY FIGURE 7

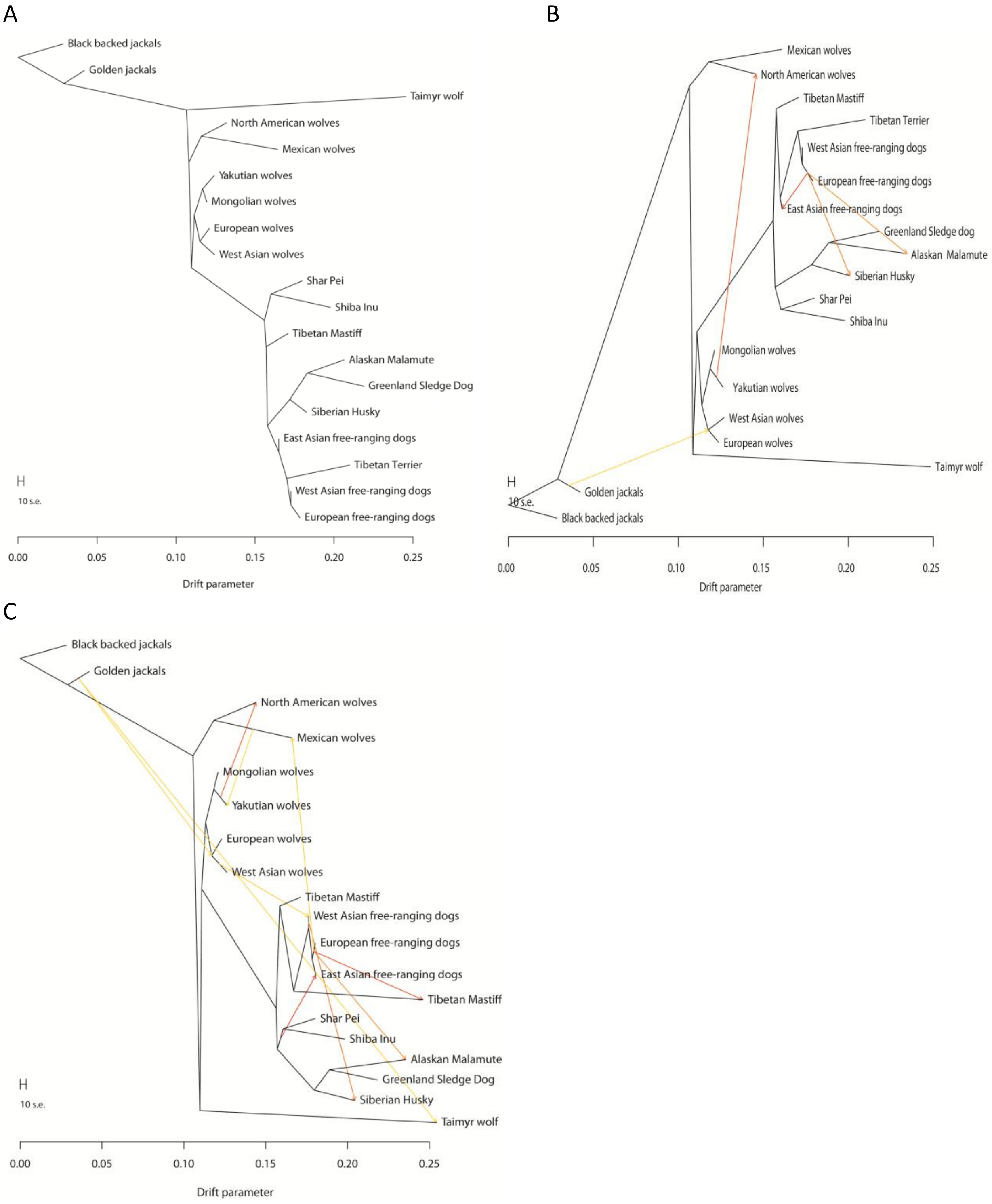

D

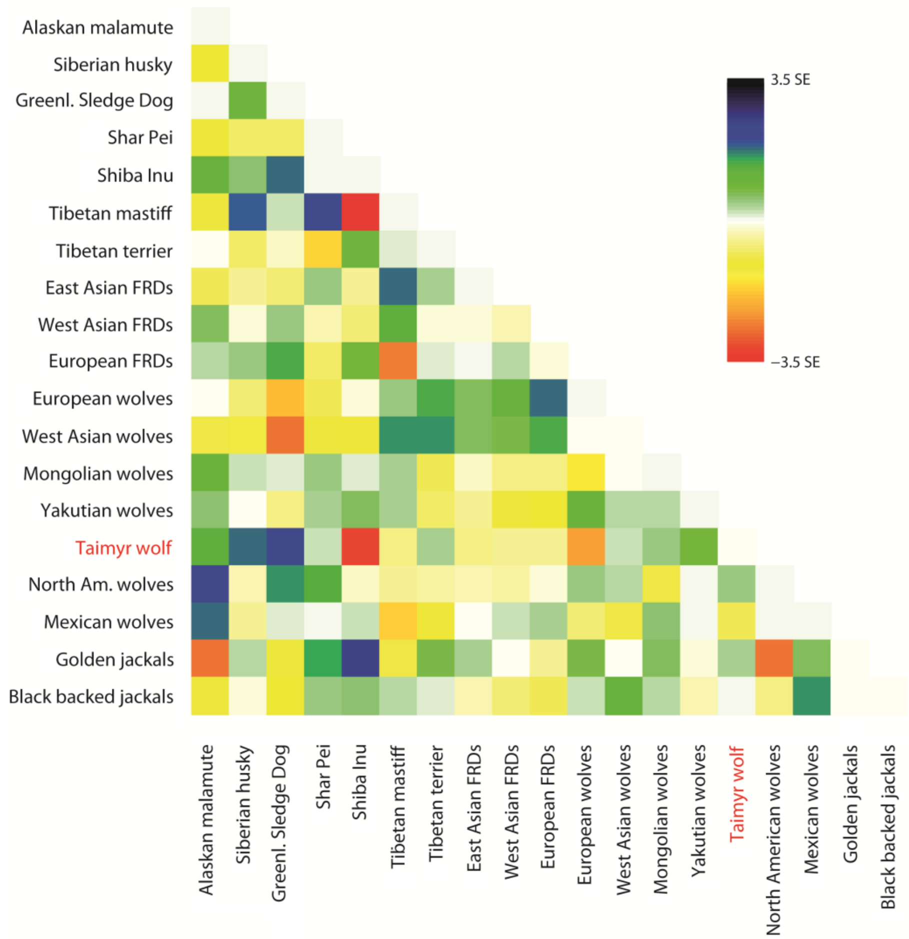


Supplementary Figure 7. Ancestry relationships between the Taimyr and modern wolf and dog populations, excluding populations with known admixture history. The dendrograms were reconstructed in TreeMix (A) without assuming gene flow, (B) assuming 5 events of gene flow. (C) assuming 10 events of gene flow. The colours of arrows reflect the intensity of gene flow, from lowest (yellow) to highest (red). (D) Plot of residuals for the phylogenetic reconstruction from figure 7C. "North American wolves" represent all North American populations studied except Minnesota wolves and Mexican wolves.

## SUPPLEMENTARY FIGURE 8

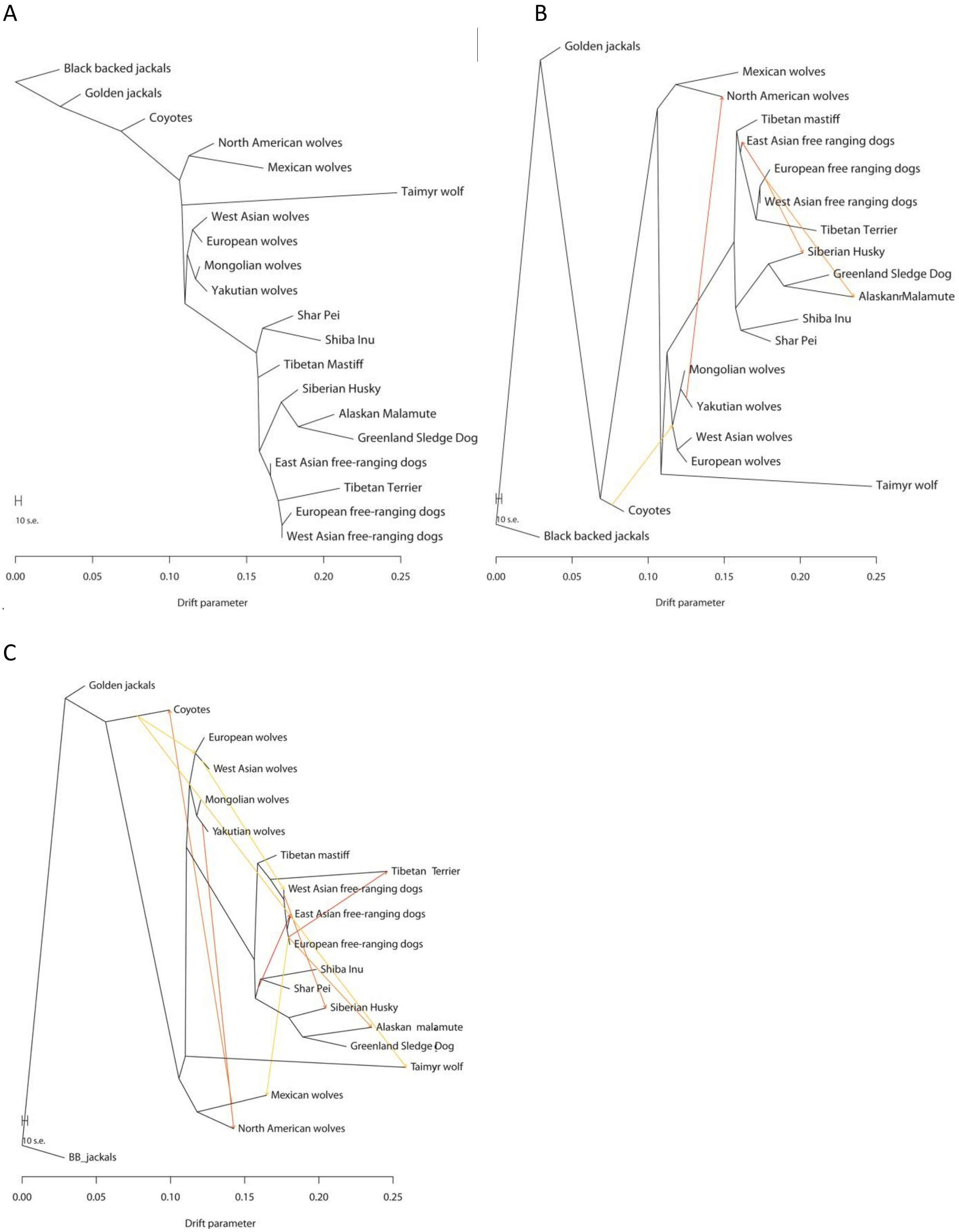

C

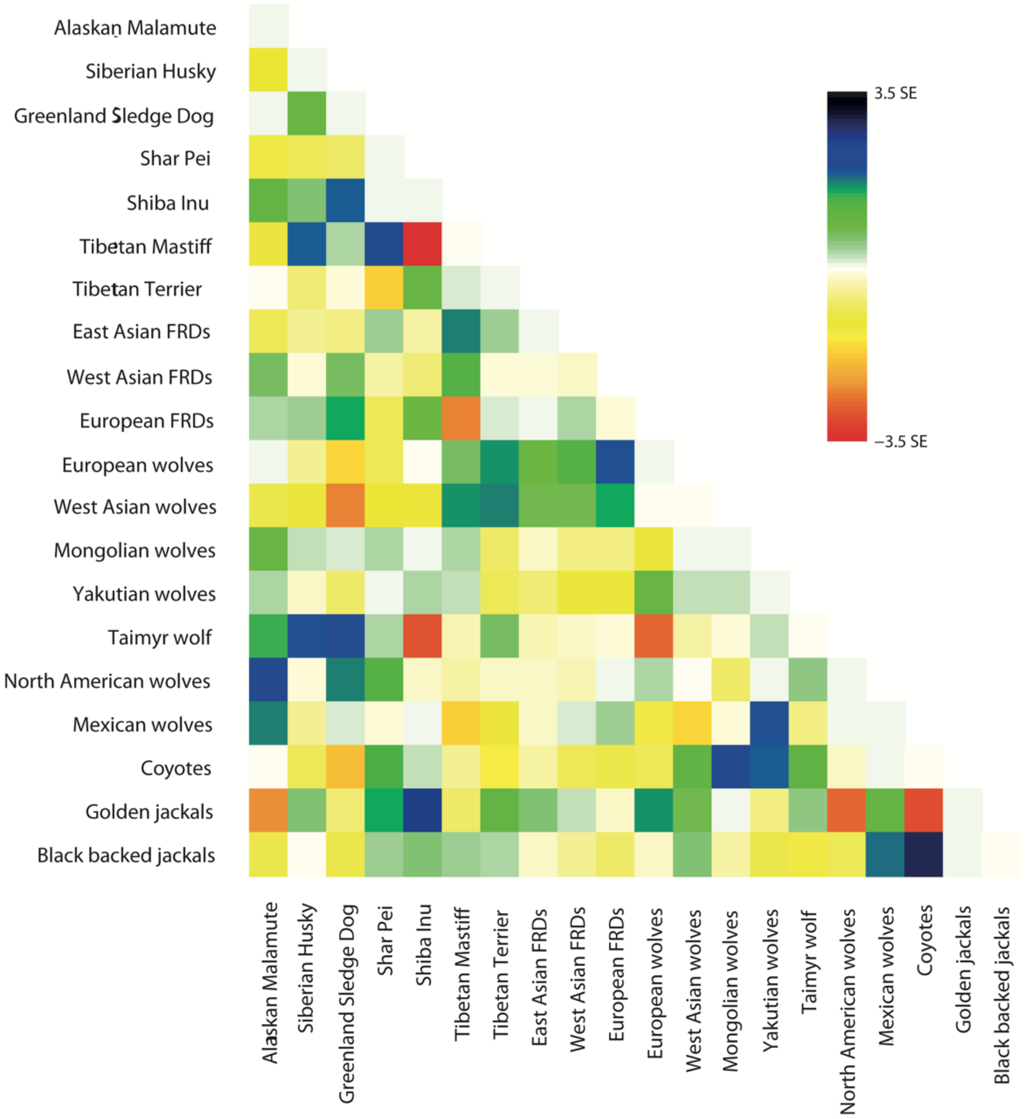


Supplementary Figure 8. Ancestry relationships between the Taimyr and modern wolf and dog populations, excluding populations with known admixture history, with the exception of coyotes. The dendrograms were reconstructed in TREEMIX (A) without assuming gene flow, (B) assuming 5 events of gene flow. (C) assuming 10 events of gene flow. The colours of arrows reflect the intensity of gene flow, from lowest (yellow) to highest (red). (D) Plot of residuals for the phylogenetic reconstruction from figure 8C. "North American wolves" represent all North American populations studied except Minnesota wolves and Mexican wolves.

## SUPPLEMENTARY FIGURE 9

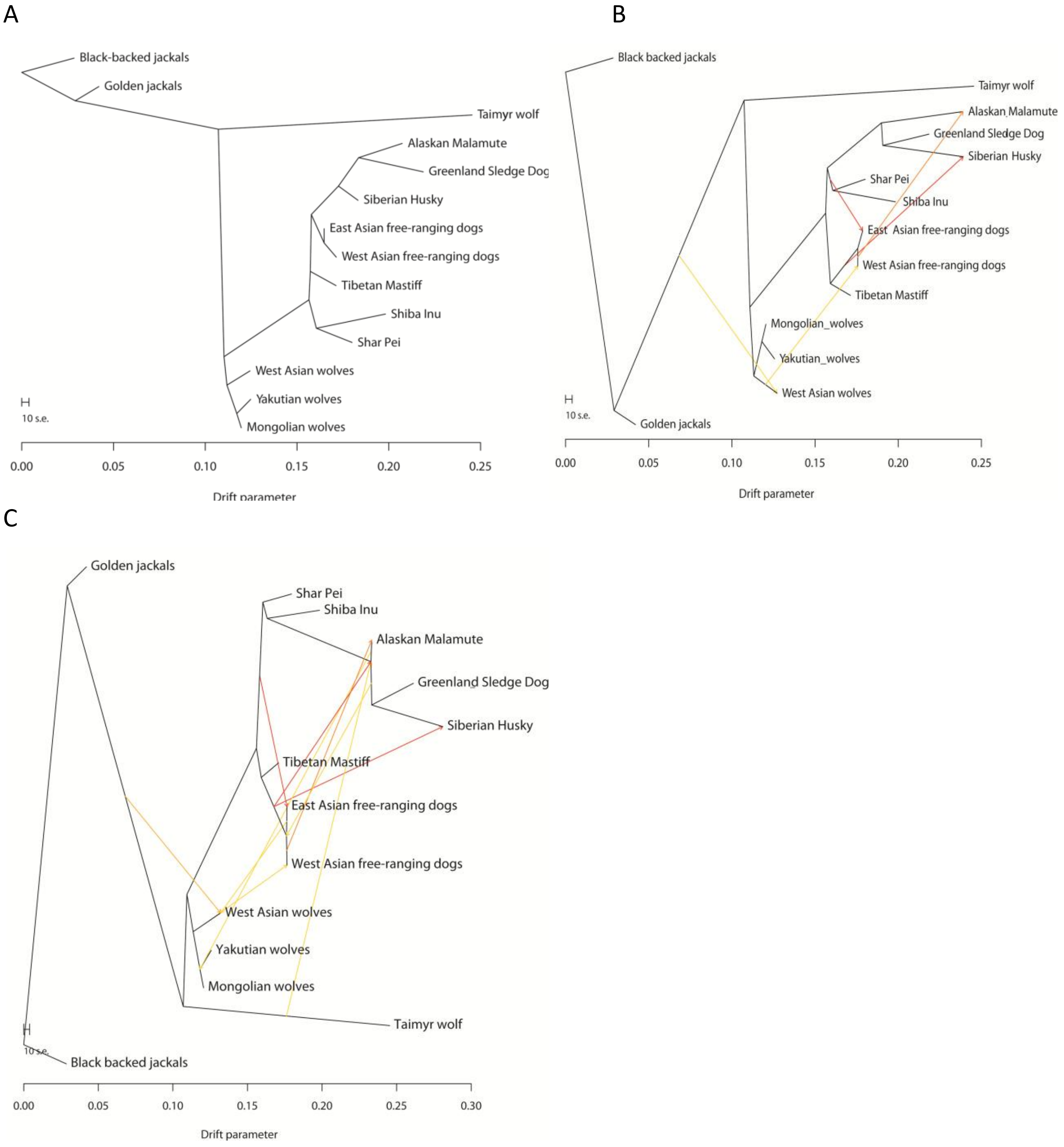

D

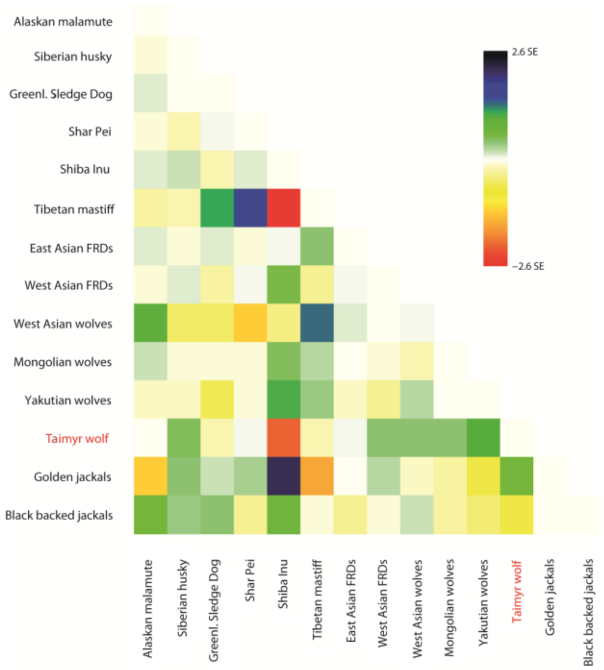


Supplementary Figure 9. Ancestry relationships among Asian wolf and dog populations. Populations from other continents were removed from the analysis. The dendrograms were reconstructed in TreeMix (A) without assuming gene flow, (B) assuming 5 events of gene flow. (C) assuming 10 events of gene flow. The colours of arrows reflect the intensity of gene flow, from lowest (yellow) to highest (red). (D) Plot of residuals for the phylogenetic reconstruction from figure 9C.

## SUPPLEMENTARY FIGURE 10

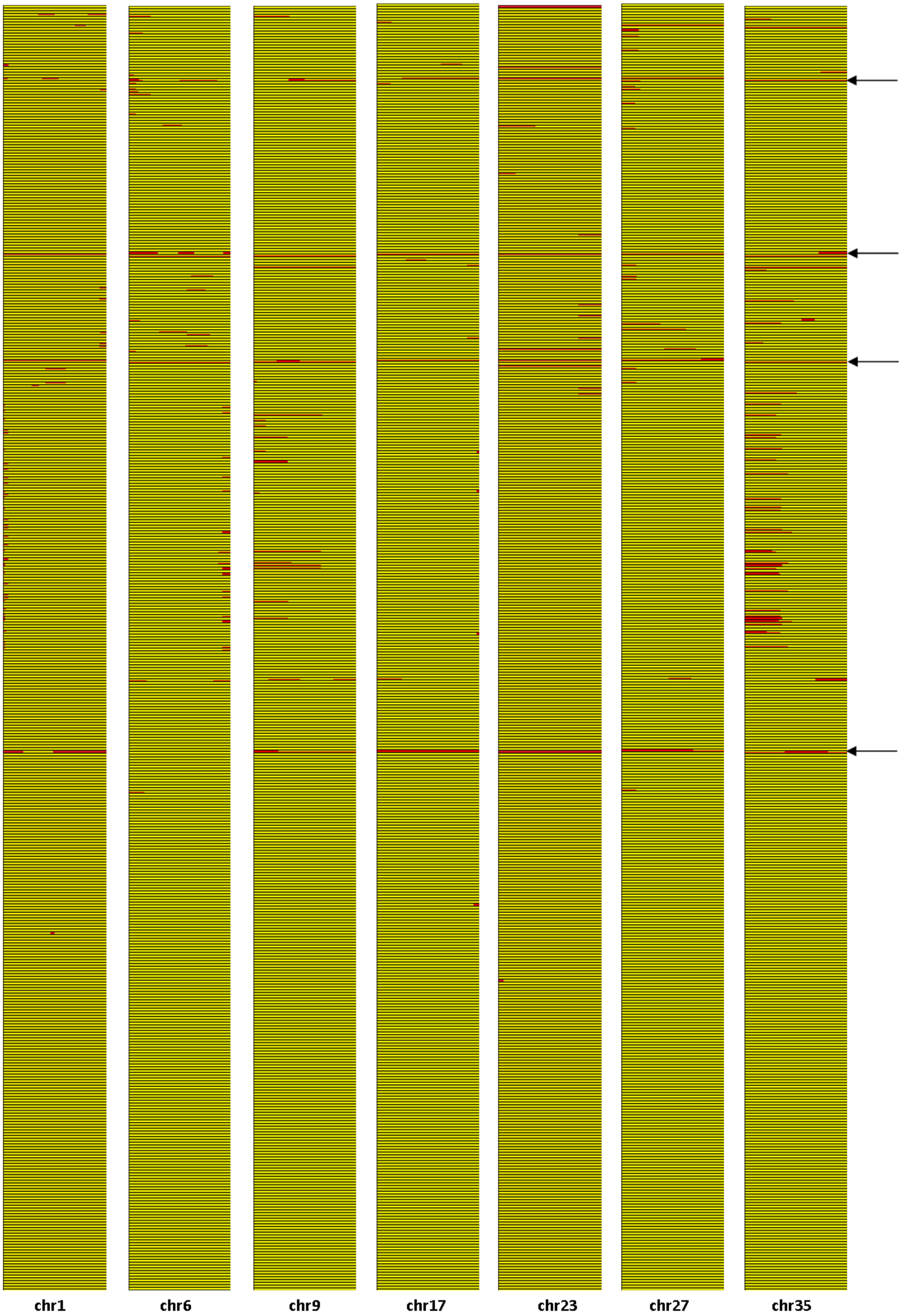


Supplementary Figure 10. Results of the ancestry block analysis in wolf populations, shown for selected chromosomes. Only a part of the ancestry graph representing wolves is shown for

each chromosome, while dogs included in the analysis are not shown, because of the large sizes of these graphs. Each line represents one individual, yellow colour represents wolf ancestry and red colour represents dog ancestry. The Taimyr wolf is placed at the top of the graph. F1 hybrids and recent backcrosses are indicated with arrows.

## SUPPLEMENTARY FIGURE 11

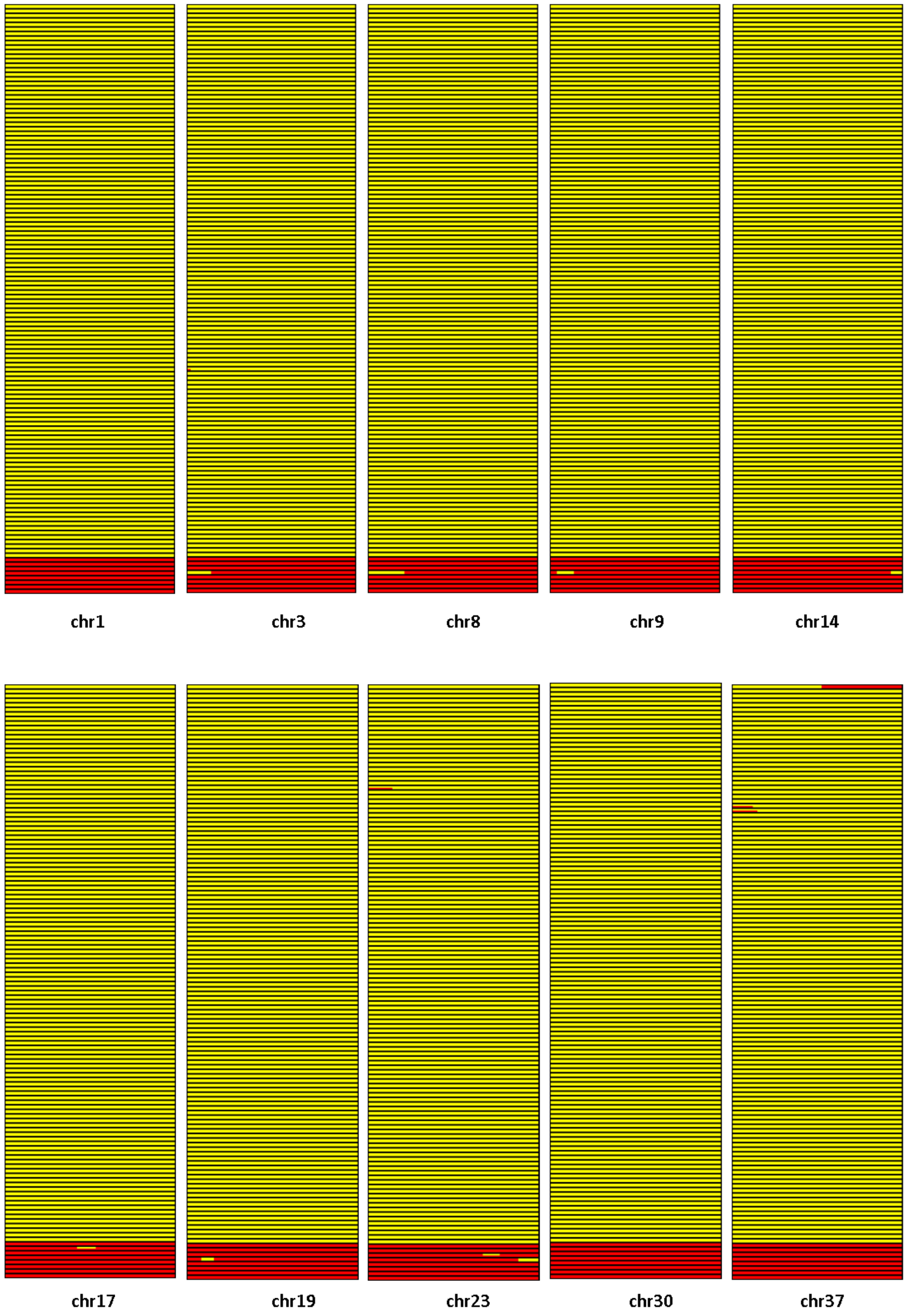


Supplementary Figure 11. Results of the ancestry block analysis in Eurasian wolves and golden jackals, shown for selected chromosomes. Each line represents one individual, yellow colour represents wolf ancestry and red colour represents jackal ancestry. The Taimyr wolf, placed at the top of the graph, shows a jackal ancestry in a large fragment of chromosome 37.

## SUPPLEMENTARY FIGURE 12

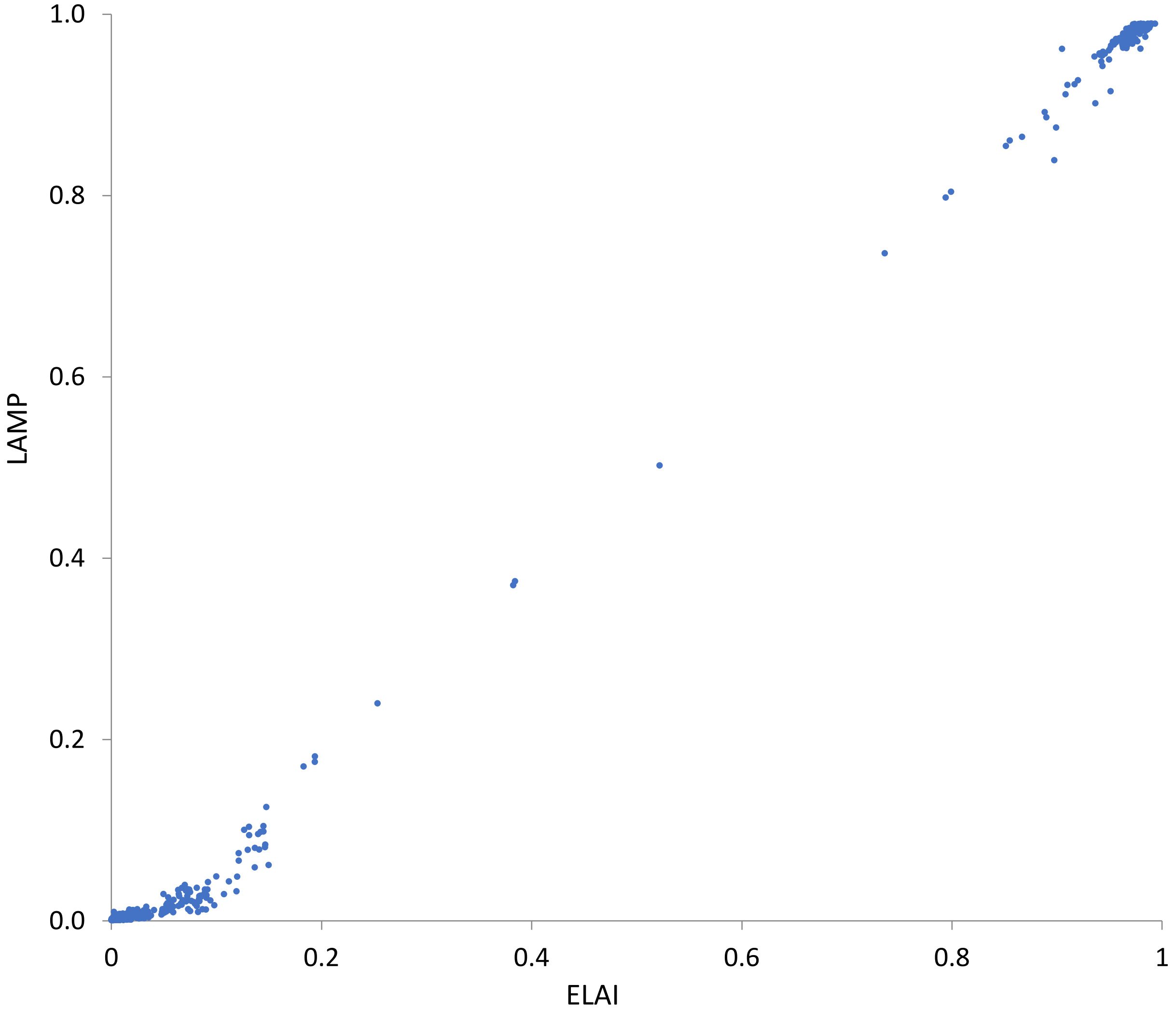


Supplementary Figure 12. Comparison of the percentage of wolf admixture estimated for grey wolves and domestic dogs using LAMP (with the assumption of 10 generations since admixture) and ELAI (with the assumption of continuous admixture throughout last 100 generations). Pearson correlation coefficient R= 0.9991, P<0.0001. Both analyses were carried out for the full sets of loci, without excluding loci missing for the Taimyr wolf; LAMP excluded loci in strong LD ($r^2$>0.1). The largest differences occur for individuals with small admixture proportions (less than 0.05), especially for pure-bred dogs, where ELAI inferred higher admixture proportions than Lamp, consistent with the fact that cross-breeding with wolves could not occur in the recent 10 generations due to close breeding, but could have taken place throughout a longer time scale.

## SUPPLEMENTARY FIGURE 13

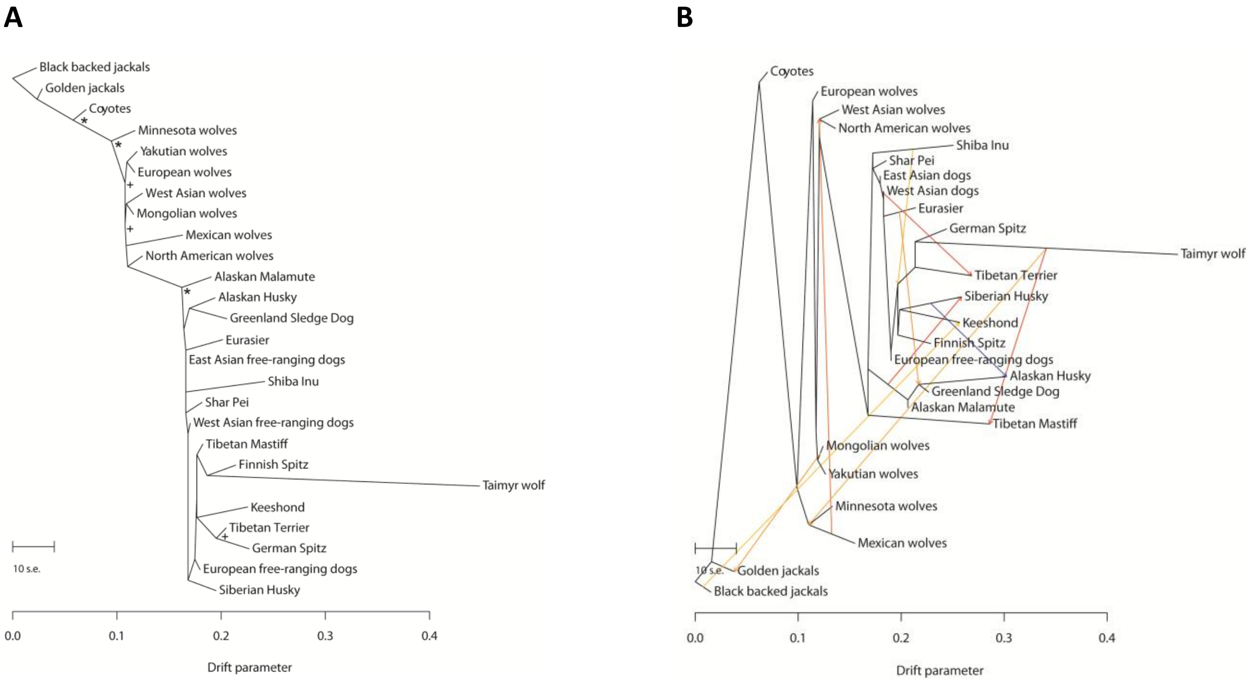


Supplementary Figure 13. Ancestry relationships among wolf and dog populations based on chromosome 23 only, reconstructed in TREEMIX (A) without assuming gene flow, (B) assuming 10 events of gene flow. The colours of arrows reflect the intensity of gene flow, from lowest (yellow) to highest (red). "North American wolves" represent all North American populations studied except Minnesota wolves and Mexican wolves. Nodes with bootstrap support above 50% are marked with a plus symbol, and nodes with 95%-100% bootstrap support are marked with an asterisk.

## SUPPLEMENTARY FIGURE 14

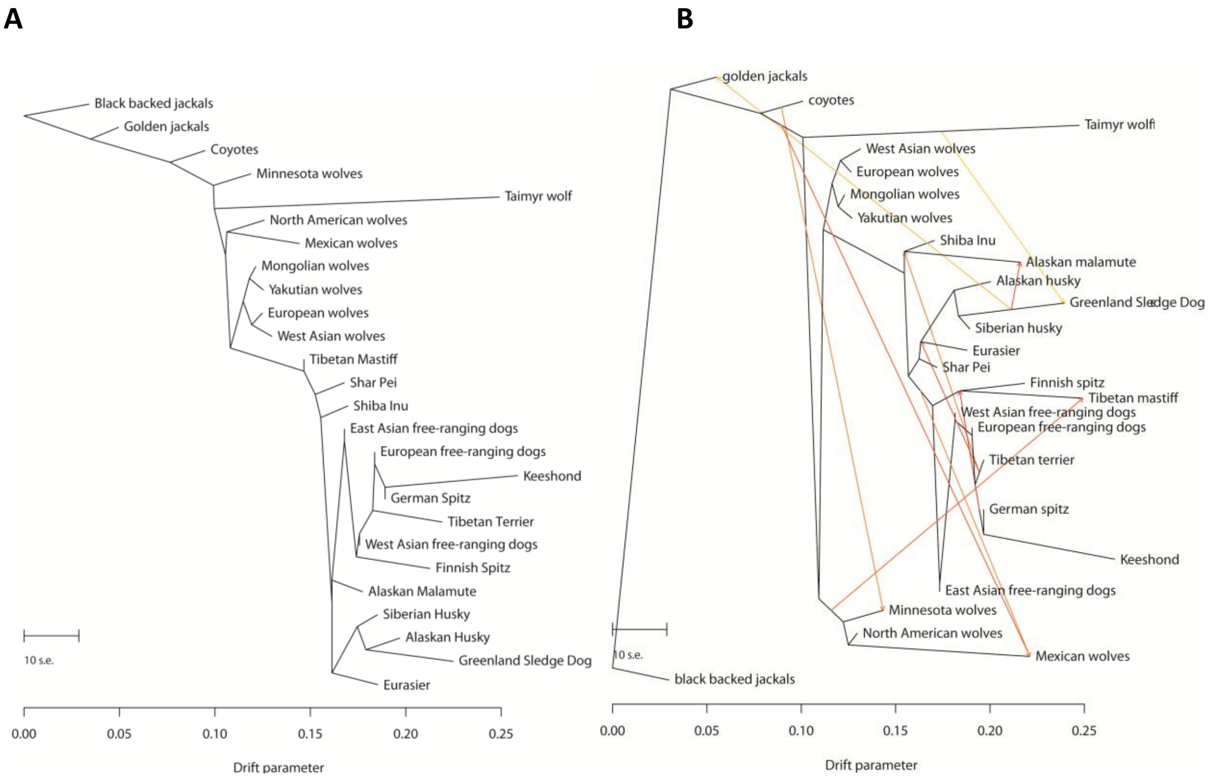


Supplementary Figure 14. Ancestry relationships among wolf and dog populations based on chromosome 37 only, reconstructed in TreeMix (A) without assuming gene flow, (B) assuming 10 events of gene flow. The colours of arrows reflect the intensity of gene flow, from lowest (yellow) to highest (red). "North American wolves" represent all North American populations studied except Minnesota and Mexican wolves.

**Supplementary Table 1.** List of samples included in the dataset analysed. The number of samples in overrepresented populations was subsequently reduced via random sampling to ensure the balanced representation of wolves and dogs from different regions.

| Species/subspecies | Country | N | Source |
|---|---|---|---|
| grey wolf | Georgia | 11 | this study |
| grey wolf | Mongolia | 14 | this study |
| grey wolf | Nagorny Karabakh | 15 | this study |
| grey wolf | Saudi Arabia | 2 | this study |
| grey wolf | Yakutia, Russia | 41 | this study |
| grey wolf | Bulgaria | 38 | this study |
| grey wolf | Belarus | 8 | Stronen et al. 2013 |
| grey wolf | Bulgaria | 2 | Stronen et al. 2013 |
| grey wolf | Croatia | 4 | Stronen et al. 2013 |
| grey wolf | Finland | 5 | Stronen et al. 2013 |
| grey wolf | Greece | 2 | Stronen et al. 2013 |
| grey wolf | Latvia | 6 | Stronen et al. 2013 |
| grey wolf | Poland | 8 | Stronen et al. 2013 |
| grey wolf | Russia | 5 | Stronen et al. 2013 |
| grey wolf | Slovakia | 1 | Stronen et al. 2013 |
| grey wolf | Ukraine | 16 | Stronen et al. 2013 |
| grey wolf | British Columbia | 35 | Cronin et al. 2015 |
| grey wolf | Idaho | 23 | Cronin et al. 2015 |
| grey wolf | Interior Alaska | 40 | Cronin et al. 2015 |
| grey wolf | Minnesota | 20 | Cronin et al. 2015 |
| grey wolf | Montana | 16 | Cronin et al. 2015 |
| grey wolf | New Mexico | 8 | Cronin et al. 2015 |
| grey wolf | Southeast Alaska | 138 | Cronin et al. 2015 |
| grey wolf | Wyoming | 25 | Cronin et al. 2015 |
| grey wolf | New Mexico, Arizona | 87 | Fitak et al. 2018 |
| grey wolf | Europe | 12 | Vaysse et al. 2011 |
| grey wolf | North America | 1 | Vaysse et al. 2011 |
| grey wolf (Late Pleistocene) | Taimyr Peninsula, Russia | 1 | Skoglund et al. 2015 |
| free-ranging dog | Armenia | 25 | Pilot et al. 2015 |
| free-ranging dog | Bulgaria | 9 | Pilot et al. 2015 |
| free-ranging dog | Central Russia | 16 | Pilot et al. 2015 |
| free-ranging dog | China | 9 | Pilot et al. 2015 |
| free-ranging dog | East Russia | 19 | Pilot et al. 2015 |
| free-ranging dog | Iraq | 8 | Pilot et al. 2015 |
| free-ranging dog | Kazakhstan | 20 | Pilot et al. 2015 |
| free-ranging dog | Mongolia | 27 | Pilot et al. 2015 |
| free-ranging dog | Poland | 21 | Pilot et al. 2015 |
| free-ranging dog | Saudi Arabia | 27 | Pilot et al. 2015 |

| | | | |
|---|---|---|---|
| free-ranging dog | Slovenia | 13 | Pilot et al. 2015 |
| free-ranging dog | Tajikistan | 19 | Pilot et al. 2015 |
| free-ranging dog | Thailand | 21 | Pilot et al. 2015 |
| free-ranging dog | China | 6 | Frantz et al. 2015 |
| free-ranging dog | India | 2 | Frantz et al. 2015 |
| free-ranging dog | Portugal | 2 | Frantz et al. 2015 |
| pure-bred dogs (various breeds) | N/A | 446 | Vaysse et al. 2011 |
| pure-bred dogs (various breeds) | N/A | 192 | Pilot et al. 2015 |
| pure-bred dogs (2 breeds) | Asia | 12 | Frantz et al. 2015 |
| mixed-breed dog | North America | 36 | Fitak et al. 2018 |
| mixed-breed dog (Alaskan husky) | Alaska | 10 | Vernau et al. 2013 |
| Neolithic dog | Ireland | 1 | Frantz et al. 2015 |
| coyote | Alaska | 2 | Fitak et al. 2018 |
| coyote | Arizona | 15 | Fitak et al. 2018 |
| coyote | Colorado | 1 | Fitak et al. 2018 |
| coyote | Mississipi | 2 | Fitak et al. 2018 |
| coyote | Nebraska | 1 | Fitak et al. 2018 |
| coyote | Nevada | 1 | Fitak et al. 2018 |
| coyote | Wisconsin | 2 | Fitak et al. 2018 |
| coyote | Wyoming | 6 | Fitak et al. 2018 |
| golden jackal | Bulgaria | 3 | this study |
| golden jackal | Nagorny Karabakh | 4 | this study |
| black backed jackal | Namibia | 3 | this study |
| golden jackal | Armenia and Georgia | 5 | included in Suppl. Fig. 5 |
| golden jackal | Bulgaria | 10 | included in Suppl. Fig. 5 |
| golden jackal | Croatia | 2 | included in Suppl. Fig. 5 |
| golden jackal | Estonia | 2 | included in Suppl. Fig. 5 |
| golden jackal | Greece | 10 | included in Suppl. Fig. 5 |
| golden jackal | Hungary | 43 | included in Suppl. Fig. 5 |
| golden jackal | Serbia | 14 | included in Suppl. Fig. 5 |
| golden jackal | Slovenia | 2 | included in Suppl. Fig. 5 |
| golden jackal | Romania | 1 | included in Suppl. Fig. 5 |
| golden jackal | Ukraine | 5 | included in Suppl. Fig. 5 |
| black backed jackal | Namibia | 6 | included in Suppl. Fig. 5 |

**Supplementary Table 2.** Heterozygosity in worldwide wolf populations. North American wolves are more distant relatives of domestic dogs than Eurasian wolves, and therefore their lower heterozygosity may result at least in part from ascertainment bias.

| Region | Continent | Ho | He |
|---|---|---|---|
| Latvia/Russia | Europe | 0.280 | 0.273 |
| Belarus/Poland | Europe | 0.271 | 0.276 |
| Bulgaria | Europe | 0.253 | 0.256 |
| Georgia | Asia | 0.273 | 0.263 |
| Nagorny Karabakh | Asia | 0.274 | 0.260 |
| Mongolia | Asia | 0.271 | 0.273 |
| Yakutia | Asia | 0.272 | 0.262 |
| South-East Alaska | N. America | 0.187 | 0.211 |
| Interior Alaska | N. America | 0.248 | 0.239 |
| British Columbia | N. America | 0.242 | 0.247 |
| Montana | N. America | 0.242 | 0.231 |
| Idaho | N. America | 0.251 | 0.244 |
| Wyoming | N. America | 0.254 | 0.241 |
| Minnesota | N. America | 0.235 | 0.231 |
| New Mexico | N. America | 0.163 | 0.171 |

**Supplementary Table 3.** Admixture proportions in the Taimyr wolf estimated in ADMIXTURE assuming the number of clusters (K) from 3 to 15. To make the results for different K-values directly comparable, the table includes only three categories: grey wolves, domestic dogs, and coyotes/jackals (including coyotes, golden jackals and black backed jackals). If more than one cluster for one category was distinguished at a given K value (e.g. two clusters distinguishing different wolf populations), the inferred admixture proportions were summed up.

| K | assignment | | | N clusters | | |
|---|---|---|---|---|---|---|
| | wolves | dogs | coyotes/jackals | wolf | dog | coyotes/jackals |
| 3 | 0.7415 | 0.0902 | 0.1683 | 1 | 1 | 1 |
| 4 | 0.7609 | 0.0753 | 0.1638 | 2 | 1 | 1 |
| 5 | - | 0.1073 | - | 2 | 3 | 0* |
| 6 | 0.7320 | 0.1070 | 0.1611 | 2 | 3 | 1 |
| 7 | 0.7309 | 0.1068 | 0.1622 | 2 | 3 | 2 |
| 8 | 0.7362 | 0.1060 | 0.1578 | 3 | 3 | 2 |
| 9 | 0.7608 | 0.0907 | 0.1485 | 4 | 4 | 1 |
| 10 | 0.7609 | 0.0905 | 0.1487 | 4 | 5 | 1 |
| 11 | 0.7602 | 0.0903 | 0.1495 | 4 | 5 | 2 |
| 12 | 0.7742 | 0.0879 | 0.1379 | 5 | 6 | 1 |
| 13 | 0.7582 | 0.0938 | 0.1480 | 5 | 7 | 1 |
| 14 | 0.7738 | 0.0878 | 0.1384 | 6 | 6 | 2 |
| 15 | 0.7754 | 0.0933 | 0.1313 | 6 | 7 | 2 |
| mean | 0.7554 | 0.0944 | 0.1513 | | | |
| S.D. | 0.0163 | 0.0097 | 0.0116 | | | |

*coyotes and jackals clustered with NA wolves

**Supplementary Table 4.** Assignment of populations to three genetic clusters corresponding to dog, wolves and other wild canids (coyotes, golden jackals and black backed jackals), inferred in ADMIXTURE assuming 3 clusters (K=3).

| Population | N | dogs | wolves | coyotes & jackals |
|---|---|---|---|---|
| Taimyr wolf | 1 | 0.09 | 0.74 | 0.17 |
| Yakutian wolves | 37 | 0.02-0.04 | 0.92-0.96 | 0.01-0.04 |
| Mongolian wolves | 14 | 0.04-0.08 | 0.87-0.90 | 0.04-0.06 |
| Georgian wolves | 11 | 0.04-0.06 | 0.87-0.90 | 0.06-0.07 |
| Nagorny Karabakh wolves | 15 | 0.04-0.06 | 0.87-0.91 | 0.05-0.07 |
| Saudi Arabian wolves | 2 | 0.13 | 0.74-0.76 | 0.11-0.13 |
| North European wolves | 62 | 0.01-0.26 | 0.73-0.99 | 0.00001-0.017 |
| South European wolves | 47 | 0.009-0.16 | 0.83-0.99 | 0.00001-0.01 |
| North American wolves | 102 | 0.00001 | 0.95-0.99 | 0.01-0.05 |
| Mexican wolves | 15 | 0.03-0.06 | 0.81-0.84 | 0.13-0.15 |
| Minnesota wolves | 15 | 0.00001 | 0.72-0.79 | 0.21-0.28 |
| Wolf-dog hybrid (Yakutia) | 1 | 0.462997 | 0.51629 | 0.020713 |
| Admixed North American wolves | 2 | 0.14-0.59 | 0.37-0.84 | 0.03-0.04 |
| East Asian dogs | 35 | 0.70-0.99 | 0.004-0.27 | 0.004-0.04 |
| East Asian and Arctic dog breeds | | 0.73-0.85 | 0.14-0.24 | 0.01-0.05 |
| Middle Eastern dogs | 17 | 0.82-0.89 | 0.09-0.16 | 0.005-0.03 |
| Indian dogs | 2 | 0.72-0.81 | 0.16-0.23 | 0.03-0.05 |
| Central and West Asian dogs | 39 | 0.91-0.99 | 0.00001-0.08 | 0.00001-0.01 |
| European dogs | 28 | 0.95-0.99 | 0.00001-0.05 | 0.0003-0.02 |
| European dog breeds | 90 | 0.90-0.99 | 0.00001-0.09 | 0.00001-0.01 |
| Mix-bred North American dogs | 38 | 0.90-0.99 | 0.00001-0.10 | 0.00001-0.01 |
| Samoyed | 2 | 0.89 | 0.08-0.09 | 0.02 |
| Eurasier | 12 | 0.84-0.92 | 0.07-0.15 | 0.01-0.03 |
| Admixed European dog | 1 | 0.88 | 0.10 | 0.02 |
| Admixed Central Asian dog | 1 | 0.85 | 0.14 | 0.01 |
| Czechoslovakian wolfdogs | 3 | 0.82-0.85 | 0.15-0.17 | 0.007-0.013 |
| Saarloos wolfdogs | 2 | 0.61-0.75 | 0.23-0.35 | 0.01-0.04 |

| | | | | |
|---|---|---|---|---|
| Neolithic Irish dog | 1 | 0.95 | 0.05 | 0.002 |
| Coyotes | 19 | 0.00001 | 0.00001 | 0.99998 |
| Coyotes (Wisconsin, Mississippi, Wyoming) | 10 | 0.00001-0.11 | 0.00001-0.21 | 0.71-0.99 |
| Golden jackals | 7 | 0.00001 | 0.00001 | 0.99998 |
| Golden jackal (admixed) | 1 | 0.051855 | 0.00001 | 0.948135 |
| Black-backed jackals | 3 | 0.00001 | 0.00001 | 0.99998 |
| Andean fox | 1 | 0.00001 | 0.00001 | 0.99998 |

**Supplementary Table 5.** Proportion of wolf ancestry in different canid populations inferred using LAMP ancestry blocks analysis for wolf-dog and wolf-golden jackal admixture.

| Population | wolf-dog admixture | wolf-golden jackal admixture |
|---|---|---|
| Taimyr wolf | 0.971 | 0.988 |
| West Asian wolves | 0.727-1.00 | 0.998-1.00 |
| East Asian wolves | 0.902-1.00 | 0.999-1.00 |
| East Asian F1 wolf-dog hybrid | 0.500 | 0.999 |
| European wolf-dog backcross | 0.592 | 1 |
| European wolves | 0.923-0.998 | 1 |
| Mexican wolves | 0.962-0.999 | - |
| North American wolves | 0.991-1.00 | - |
| Minnesota wolves | 0.996-1.00 | - |
| North American wolf backcross | 0.861 | - |
| North American dog backcross | 0.339 | - |
| European dog breeds | 0.000-0.003 | - |
| East Asian dog breeds | 0.001-0.034 | - |
| Eurasier | 0.000-0.006 | - |
| Alaskan husky | 0.000-0.008 | - |
| Czechoslovakian wolfdogs | 0.122 | - |
| Saarloos wolfdogs | 0.177-0.327 | - |
| East Asian free-ranging dogs | 0.000-0.069 | - |
| West Eurasian free-ranging dogs | 0.000-0.007 | - |
| North American free-ranging dogs | 0.000-0.013 | - |
| Neolithic Irish dog | 0.002 | - |
| Golden jackals | - | 0.001-0.017 |

**Supplementary Table 6.** Proportion of wolf ancestry in different canid populations inferred using LAMP and ELAI ancestry blocks analyses for wolf-dog admixture.

| Population | LAMP | ELAI |
|---|---|---|
| Taimyr wolf | 0.971 | - |
| West Asian wolves | 0.727-1.00 | 0.897-1.00 |
| East Asian wolves | 0.902-1.00 | 0.967-1.00 |
| East Asian F1 wolf-dog hybrid | 0.500 | 0.522 |
| European wolves | 0.923-0.998 | 0.942-1.00 |
| Mexican wolves | 0.962-0.999 | 0.936-0.999 |
| North American wolves | 0.991-1.00 | 0.989-1.00 |
| Minnesota wolves | 0.996-1.00 | 1.00 |
| North American wolf backcross | 0.861 | 0.851 |
| North American dog backcross | 0.339 | 0.384 |
| European dog breeds | 0.000-0.003 | 0.000-0.018 |
| East Asian dog breeds | 0.001-0.034 | 0.073-0.149 |
| Eurasier | 0.000-0.006 | 0.021-0.072 |
| Alaskan husky | 0.000-0.008 | 0.048-0.071 |
| Czechoslovakian wolfdogs | 0.122 | 0.183-0.194 |
| Saarloos wolfdogs | 0.177-0.327 | 0.253-0.382 |
| East Asian free-ranging dogs | 0.000-0.069 | 0.000-0.140 |
| West Eurasian free-ranging dogs | 0.000-0.007 | 0.000-0.075 |
| North American free-ranging dogs | 0.000-0.013 | 0.000-0.019 |
| Neolithic Irish dog | 0.002 | 0.018 |

**Supplementary Table 7.** Proportion of dog and golden jackal ancestry in modern and ancient canid populations in chromosome 23 inferred using ADMIXTURE and LAMP. ADMIXTURE software infers long-term patterns of admixture, while the LAMP analysis was run assuming admixture in last 10 generations of ancestors.

| Population | Dog ancestry ADMIXTURE | Golden jackal[a] ancestry ADMIXTURE | Dog ancestry LAMP | Golden jackal ancestry LAMP |
|---|---|---|---|---|
| Taimyr wolf | 0.64 | 0.00 | 1.00 | 0.00 |
| modern Eurasian wolves | 0.00-0.31 | 0.00-0.29 | 0.00-0.50[b] | 0.00-0.07 |
| modern dogs | 0.49-0.96 | 0.00-0.14 | 0.50-1.00 | - |
| golden jackal | 0.00-0.06 | 0.91-1.00 | - | 0.89-1.00 |

a. Assignment to the cluster consisting of golden jackals as well as black-backed jackals and coyotes, the last two absent from Eurasia.

b. only 11 wolves had assignment values >0.00

**Supplementary Table 8**. Results of ADMIXTURE analysis for K=3. Data for individuals deriving from different datasets is displayed with different colours. This result is shown to demonstrate the accuracy of the dataset merging by confirming joint clustering of individuals belonging to the same populations and species, but originating from different datasets. This result is shown as an example; the assessment of the accuracy of merging was carried out using both ADMIXTURE analyses for different K-values and PCA plots for the complete merging dataset, before reducing the number of individuals and loci. The clustering of individuals from different datasets was assessed at the level of regional populations of wild canids and individual breeds of domestic dogs.

| ID1 | ID2 | species | location | dataset | dogs | wolves | coyotes/jackals |
|---|---|---|---|---|---|---|---|
| 192 | 8957 | black coated wolf-like canid | Yakutia | New Dataset | 0.462997 | 0.51629 | 0.020713 |
| 186 | 8937 | black-backed jackal | Namibia | New Dataset | 0.00001 | 0.00001 | 0.99998 |
| 187 | 8938 | black-backed jackal | Namibia | New Dataset | 0.00001 | 0.00001 | 0.99998 |
| 188 | 8939 | black-backed jackal | Namibia | New Dataset | 0.00001 | 0.00001 | 0.99998 |
| 3 | CO_Alaska | coyote | North America | Fitak et al. 2018 | 0.00001 | 0.00001 | 0.99998 |
| 2 | CO_Alaska | coyote | North America | Fitak et al. 2018 | 0.00001 | 0.00001 | 0.99998 |
| 5 | CO_Arizona | coyote | North America | Fitak et al. 2018 | 0.00001 | 0.00001 | 0.99998 |
| 6 | CO_Arizona | coyote | North America | Fitak et al. 2018 | 0.00001 | 0.00001 | 0.99998 |
| 7 | CO_Arizona | coyote | North America | Fitak et al. 2018 | 0.00001 | 0.00001 | 0.99998 |
| 8 | CO_Arizona | coyote | North America | Fitak et al. 2018 | 0.00001 | 0.00001 | 0.99998 |
| 9 | CO_Arizona | coyote | North America | Fitak et al. 2018 | 0.00001 | 0.00001 | 0.99998 |
| 10 | CO_Arizona | coyote | North America | Fitak et al. 2018 | 0.00001 | 0.00001 | 0.99998 |
| 11 | CO_Arizona | coyote | North America | Fitak et al. 2018 | 0.00001 | 0.00001 | 0.99998 |
| 12 | CO_Arizona | coyote | North America | Fitak et al. 2018 | 0.00001 | 0.00001 | 0.99998 |
| 13 | CO_Arizona | coyote | North America | Fitak et al. 2018 | 0.00001 | 0.00001 | 0.99998 |
| 14 | CO_Arizona | coyote | North America | Fitak et al. 2018 | 0.00001 | 0.00001 | 0.99998 |
| 15 | CO_Arizona | coyote | North America | Fitak et al. 2018 | 0.00001 | 0.00001 | 0.99998 |
| 16 | CO_Arizona | coyote | North America | Fitak et al. 2018 | 0.00001 | 0.00001 | 0.99998 |
| 17 | CO_Arizona | coyote | North America | Fitak et al. 2018 | 0.00001 | 0.00001 | 0.99998 |

| | | | | | | | |
|---|---|---|---|---|---|---|---|
| 18 | CO_Arizona | coyote | North America | Fitak et al. 2018 | 0.00001 | 0.00001 | 0.99998 |
| 19 | CO_Arizona | coyote | North America | Fitak et al. 2018 | 0.00001 | 0.00001 | 0.99998 |
| CO | CO_Colorado | coyote | North America | Fitak et al. 2018 | 0.00001 | 0.001579 | 0.998411 |
| COYOTE-02 | CO_Mississippi | coyote | North America | Fitak et al. 2018 | 0.03807 | 0.125571 | 0.836359 |
| NE | CO_Nebraska | coyote | North America | Fitak et al. 2018 | 0.00001 | 0.00001 | 0.99998 |
| NV | CO_Nevada | coyote | North America | Fitak et al. 2018 | 0.00001 | 0.00001 | 0.99998 |
| WI-1 | CO_Wisconsin | coyote | North America | Fitak et al. 2018 | 0.028919 | 0.052475 | 0.918606 |
| WI-2 | CO_Wisconsin | coyote | North America | Fitak et al. 2018 | 0.001643 | 0.054267 | 0.94409 |
| Coyote-1 | CO_Wyoming | coyote | North America | Fitak et al. 2018 | 0.08209 | 0.208395 | 0.709514 |
| Coyote-10 | CO_Wyoming | coyote | North America | Fitak et al. 2018 | 0.00001 | 0.00001 | 0.99998 |
| Coyote-15 | CO_Wyoming | coyote | North America | Fitak et al. 2018 | 0.109266 | 0.169679 | 0.721055 |
| Coyote-16 | CO_Wyoming | coyote | North America | Fitak et al. 2018 | 0.073673 | 0.172344 | 0.753983 |
| Coyote-19 | CO_Wyoming | coyote | North America | Fitak et al. 2018 | 0.069265 | 0.126063 | 0.804672 |
| Coyote-3 | CO_Wyoming | coyote | North America | Fitak et al. 2018 | 0.00001 | 0.00001 | 0.99998 |
| 4 | 15523 | domestic dog | Alaskan husky | Fitak et al. 2018 | 0.864974 | 0.119904 | 0.015122 |
| 7 | 50501 | domestic dog | Alaskan husky | Fitak et al. 2018 | 0.87286 | 0.12713 | 0.00001 |
| 26 | 15522redo | domestic dog | Alaskan husky | Fitak et al. 2018 | 0.864073 | 0.126778 | 0.009149 |
| 29 | 50532redo | domestic dog | Alaskan husky | Fitak et al. 2018 | 0.864596 | 0.120486 | 0.014918 |
| 31 | 50537redo | domestic dog | Alaskan husky | Fitak et al. 2018 | 0.86529 | 0.1347 | 0.00001 |
| 34 | 50545redo | domestic dog | Alaskan husky | Fitak et al. 2018 | 0.871624 | 0.12002 | 0.008357 |
| 44 | 15516 | domestic dog | Alaskan husky | Fitak et al. 2018 | 0.84746 | 0.141757 | 0.010782 |
| 47 | 15524 | domestic dog | Alaskan husky | Fitak et al. 2018 | 0.853434 | 0.142167 | 0.004399 |
| 48 | 15536 | domestic dog | Alaskan husky | Fitak et al. 2018 | 0.881969 | 0.113312 | 0.004719 |
| 49 | 15509 | domestic dog | Alaskan husky | Fitak et al. 2018 | 0.866152 | 0.123135 | 0.010713 |
| 1 | 9 | domestic dog | Bulgaria | New Dataset | 0.966223 | 0.030164 | 0.003613 |
| 22 | 171 | domestic dog | Bulgaria | New Dataset | 0.882015 | 0.102678 | 0.015307 |
| 31 | 201 | domestic dog | Bulgaria | New Dataset | 0.981125 | 0.013075 | 0.0058 |
| 6 | 10BL | domestic dog | Bulgaria | Pilot et al. 2015 | 0.948641 | 0.04096 | 0.010399 |
| 12 | 11BL | domestic dog | Bulgaria | Pilot et al. 2015 | 0.946754 | 0.050096 | 0.00315 |

| | | | | | | | |
|---|---|---|---|---|---|---|---|
| 17 | 3BL | domestic dog | Bulgaria | Pilot et al. 2015 | 0.991926 | 0.003841 | 0.004233 |
| 18 | 12BL | domestic dog | Bulgaria | Pilot et al. 2015 | 0.965515 | 0.02905 | 0.005435 |
| 29 | 6BL | domestic dog | Bulgaria | Pilot et al. 2015 | 0.966334 | 0.026099 | 0.007567 |
| 41 | 8BL | domestic dog | Bulgaria | Pilot et al. 2015 | 0.967899 | 0.019446 | 0.012655 |
| 47 | 9BL | domestic dog | Bulgaria | Pilot et al. 2015 | 0.966737 | 0.029641 | 0.003623 |
| 11 | 2BL | domestic dog | Bulgaria | Pilot et al. 2015 | 0.961638 | 0.02855 | 0.009812 |
| 193 | 23A | domestic dog | Caucasus | Pilot et al. 2015 | 0.948844 | 0.044472 | 0.006684 |
| 195 | 25A | domestic dog | Caucasus | Pilot et al. 2015 | 0.945315 | 0.035898 | 0.018788 |
| 199 | 29A | domestic dog | Caucasus | Pilot et al. 2015 | 0.923896 | 0.061656 | 0.014448 |
| 201 | 31A | domestic dog | Caucasus | Pilot et al. 2015 | 0.939136 | 0.052258 | 0.008606 |
| 203 | 33A | domestic dog | Caucasus | Pilot et al. 2015 | 0.959456 | 0.022835 | 0.017709 |
| 207 | 37A | domestic dog | Caucasus | Pilot et al. 2015 | 0.94627 | 0.047456 | 0.006275 |
| 209 | 39A | domestic dog | Caucasus | Pilot et al. 2015 | 0.925257 | 0.0615 | 0.013244 |
| 212 | 42A | domestic dog | Caucasus | Pilot et al. 2015 | 0.914607 | 0.059186 | 0.026207 |
| 57 | 89R | domestic dog | Central Asia | Pilot et al. 2015 | 0.948658 | 0.04164 | 0.009702 |
| 58 | 91R | domestic dog | Central Asia | Pilot et al. 2015 | 0.955471 | 0.031652 | 0.012877 |
| 60 | 93R | domestic dog | Central Asia | Pilot et al. 2015 | 0.945818 | 0.037974 | 0.016207 |
| 64 | 97R | domestic dog | Central Asia | Pilot et al. 2015 | 0.956292 | 0.040357 | 0.003352 |
| 66 | 99R | domestic dog | Central Asia | Pilot et al. 2015 | 0.954357 | 0.035079 | 0.010565 |
| 68 | 102R | domestic dog | Central Asia | Pilot et al. 2015 | 0.957789 | 0.037954 | 0.004257 |
| 69 | 104R | domestic dog | Central Asia | Pilot et al. 2015 | 0.950857 | 0.040334 | 0.008809 |
| 72 | 107R | domestic dog | Central Asia | Pilot et al. 2015 | 0.959953 | 0.028921 | 0.011126 |
| 145 | 1KZ | domestic dog | Central Asia | Pilot et al. 2015 | 0.939477 | 0.051525 | 0.008998 |
| 147 | 3KZ | domestic dog | Central Asia | Pilot et al. 2015 | 0.924423 | 0.062142 | 0.013435 |
| 149 | 5KZ | domestic dog | Central Asia | Pilot et al. 2015 | 0.95731 | 0.034489 | 0.0082 |
| 153 | 9KZ | domestic dog | Central Asia | Pilot et al. 2015 | 0.94384 | 0.05615 | 0.00001 |
| 155 | 11KZ | domestic dog | Central Asia | Pilot et al. 2015 | 0.96829 | 0.020497 | 0.011214 |
| 160 | 16KZ | domestic dog | Central Asia | Pilot et al. 2015 | 0.952529 | 0.036423 | 0.011048 |
| 164 | 20KZ | domestic dog | Central Asia | Pilot et al. 2015 | 0.953133 | 0.038668 | 0.008199 |

| | | | | | | | |
|---|---|---|---|---|---|---|---|
| 165 | 13KZ | domestic dog | Central Asia | Pilot et al. 2015 | 0.912131 | 0.071778 | 0.01609 |
| 174 | 1TDZ | domestic dog | Central Asia | Pilot et al. 2015 | 0.920669 | 0.069271 | 0.010061 |
| 176 | 3TDZ | domestic dog | Central Asia | Pilot et al. 2015 | 0.995157 | 0.00001 | 0.004833 |
| 178 | 5TDZ | domestic dog | Central Asia | Pilot et al. 2015 | 0.911575 | 0.077169 | 0.011257 |
| 182 | 9TDZ | domestic dog | Central Asia | Pilot et al. 2015 | 0.90582 | 0.082444 | 0.011736 |
| 184 | 12TDZ | domestic dog | Central Asia | Pilot et al. 2015 | 0.937671 | 0.05121 | 0.011119 |
| 188 | 16TDZ | domestic dog | Central Asia | Pilot et al. 2015 | 0.928089 | 0.062168 | 0.009743 |
| 190 | 18TDZ | domestic dog | Central Asia | Pilot et al. 2015 | 0.851391 | 0.136578 | 0.012031 |
| 192 | 20TDZ | domestic dog | Central Asia | Pilot et al. 2015 | 0.937283 | 0.060093 | 0.002625 |
| ChineseVDog | DQ1 | domestic dog | China | Frantz et al. 2016 | 0.750788 | 0.215319 | 0.033892 |
| ChineseVDog | DQ3 | domestic dog | China | Frantz et al. 2016 | 0.762136 | 0.213344 | 0.02452 |
| ChineseVDog | LJ1 | domestic dog | China | Frantz et al. 2016 | 0.727008 | 0.248181 | 0.024811 |
| ChineseVDog | LJ4 | domestic dog | China | Frantz et al. 2016 | 0.717551 | 0.242626 | 0.039823 |
| ChineseVDog | TW04 | domestic dog | China | Frantz et al. 2016 | 0.785037 | 0.175349 | 0.039614 |
| ChineseVDog | YJ1 | domestic dog | China | Frantz et al. 2016 | 0.695368 | 0.261772 | 0.04286 |
| ChineseVDog | YJ3 | domestic dog | China | Frantz et al. 2016 | 0.698517 | 0.268906 | 0.032577 |
| 1 | 2CH | domestic dog | China | Pilot et al. 2015 | 0.833643 | 0.141057 | 0.0253 |
| 2 | 10CH | domestic dog | China | Pilot et al. 2015 | 0.842785 | 0.129613 | 0.027602 |
| 7 | 3CH | domestic dog | China | Pilot et al. 2015 | 0.814036 | 0.167593 | 0.01837 |
| 13 | 4CH | domestic dog | China | Pilot et al. 2015 | 0.836017 | 0.139323 | 0.02466 |
| 19 | 5CH | domestic dog | China | Pilot et al. 2015 | 0.83906 | 0.135056 | 0.025884 |
| 25 | 6CH | domestic dog | China | Pilot et al. 2015 | 0.807633 | 0.163873 | 0.028495 |
| 31 | 7CH | domestic dog | China | Pilot et al. 2015 | 0.834677 | 0.141619 | 0.023705 |
| 43 | 9CH | domestic dog | China | Pilot et al. 2015 | 0.866324 | 0.108959 | 0.024716 |
| 225 | 108R | domestic dog | East Asia | Pilot et al. 2015 | 0.99998 | 0.00001 | 0.00001 |
| 227 | 110R | domestic dog | East Asia | Pilot et al. 2015 | 0.965298 | 0.024486 | 0.010215 |
| 229 | 112R | domestic dog | East Asia | Pilot et al. 2015 | 0.950555 | 0.044759 | 0.004686 |
| 232 | 116R | domestic dog | East Asia | Pilot et al. 2015 | 0.945231 | 0.050298 | 0.004471 |
| 233 | 118R | domestic dog | East Asia | Pilot et al. 2015 | 0.940812 | 0.050052 | 0.009136 |

| | | | | | | | |
|---|---|---|---|---|---|---|---|
| 235 | 120R | domestic dog | East Asia | Pilot et al. 2015 | 0.938822 | 0.05472 | 0.006457 |
| 239 | 124R | domestic dog | East Asia | Pilot et al. 2015 | 0.953588 | 0.043025 | 0.003387 |
| 240 | 126R | domestic dog | East Asia | Pilot et al. 2015 | 0.988587 | 0.005029 | 0.006384 |
| IndiaVDog | ID60.realigned | domestic dog | India | Frantz et al. 2016 | 0.717588 | 0.23283 | 0.049582 |
| IndiaVDog | ID125.realigned | domestic dog | India | Frantz et al. 2016 | 0.810873 | 0.156544 | 0.032582 |
| aIrish | aIrish | domestic dog | Ireland, ancient | Frantz et al. 2016 | 0.945521 | 0.054469 | 0.00001 |
| aIrish_R | aIrish_R | domestic dog | Ireland, ancient | Frantz et al. 2016 | 0.94708 | 0.051203 | 0.001716 |
| 73 | 1MG | domestic dog | Mongolia | Pilot et al. 2015 | 0.917001 | 0.077045 | 0.005953 |
| 75 | 3MG | domestic dog | Mongolia | Pilot et al. 2015 | 0.9044 | 0.074308 | 0.021292 |
| 77 | 6MG | domestic dog | Mongolia | Pilot et al. 2015 | 0.862517 | 0.125883 | 0.0116 |
| 78 | 8MG | domestic dog | Mongolia | Pilot et al. 2015 | 0.909572 | 0.077359 | 0.013069 |
| 80 | 10MG | domestic dog | Mongolia | Pilot et al. 2015 | 0.898941 | 0.091178 | 0.009882 |
| 82 | 12MG | domestic dog | Mongolia | Pilot et al. 2015 | 0.904447 | 0.076213 | 0.01934 |
| 84 | 14MG | domestic dog | Mongolia | Pilot et al. 2015 | 0.88878 | 0.101691 | 0.009529 |
| 86 | 16MG | domestic dog | Mongolia | Pilot et al. 2015 | 0.885795 | 0.087178 | 0.027027 |
| 90 | 21MG | domestic dog | Mongolia | Pilot et al. 2015 | 0.925979 | 0.059858 | 0.014163 |
| 92 | 23MG | domestic dog | Mongolia | Pilot et al. 2015 | 0.935457 | 0.050786 | 0.013757 |
| 112 | 8156 | domestic dog | Mongolia | New Dataset | 0.853679 | 0.119979 | 0.026341 |
| 1-N3669 | DO_MixedBreed | domestic dog | North America | Fitak et al. 2018 | 0.972367 | 0.021975 | 0.005658 |
| 2-P1871 | DO_MixedBreed | domestic dog | North America | Fitak et al. 2018 | 0.972604 | 0.022591 | 0.004805 |
| 3-N3677 | DO_MixedBreed | domestic dog | North America | Fitak et al. 2018 | 0.991963 | 0.008027 | 0.00001 |
| 4-P3664 | DO_MixedBreed | domestic dog | North America | Fitak et al. 2018 | 0.996831 | 0.00001 | 0.003159 |
| 5-P3665 | DO_MixedBreed | domestic dog | North America | Fitak et al. 2018 | 0.97594 | 0.011589 | 0.01247 |
| 6-N3672 | DO_MixedBreed | domestic dog | North America | Fitak et al. 2018 | 0.973474 | 0.026516 | 0.00001 |
| 7-P3659 | DO_MixedBreed | domestic dog | North America | Fitak et al. 2018 | 0.966823 | 0.022981 | 0.010196 |
| 8-P3656 | DO_MixedBreed | domestic dog | North America | Fitak et al. 2018 | 0.980853 | 0.0136 | 0.005548 |
| 9-P3662 | DO_MixedBreed | domestic dog | North America | Fitak et al. 2018 | 0.895484 | 0.098707 | 0.00581 |
| 10-N3313 | DO_MixedBreed | domestic dog | North America | Fitak et al. 2018 | 0.973056 | 0.019932 | 0.007012 |
| 11-N1902 | DO_MixedBreed | domestic dog | North America | Fitak et al. 2018 | 0.98526 | 0.01473 | 0.00001 |

| | | | | | | | |
|---|---|---|---|---|---|---|---|
| 12-N1880 | DO_MixedBreed | domestic dog | North America | Fitak et al. 2018 | 0.974221 | 0.014368 | 0.011411 |
| 13-N1894 | DO_MixedBreed | domestic dog | North America | Fitak et al. 2018 | 0.993817 | 0.00001 | 0.006173 |
| 14-P3666 | DO_MixedBreed | domestic dog | North America | Fitak et al. 2018 | 0.955827 | 0.035034 | 0.009139 |
| 15-N3655 | DO_MixedBreed | domestic dog | North America | Fitak et al. 2018 | 0.99297 | 0.001868 | 0.005162 |
| 16-P3673 | DO_MixedBreed | domestic dog | North America | Fitak et al. 2018 | 0.982258 | 0.012318 | 0.005423 |
| 17-N3729 | DO_MixedBreed | domestic dog | North America | Fitak et al. 2018 | 0.978522 | 0.020105 | 0.001373 |
| 18-P3667 | DO_MixedBreed | domestic dog | North America | Fitak et al. 2018 | 0.992316 | 0.00001 | 0.007674 |
| 19-P1874 | DO_MixedBreed | domestic dog | North America | Fitak et al. 2018 | 0.997385 | 0.00001 | 0.002605 |
| 20-P3676 | DO_MixedBreed | domestic dog | North America | Fitak et al. 2018 | 0.976141 | 0.010036 | 0.013823 |
| 21-N3728 | DO_MixedBreed | domestic dog | North America | Fitak et al. 2018 | 0.9788 | 0.011986 | 0.009214 |
| 22-P1875 | DO_MixedBreed | domestic dog | North America | Fitak et al. 2018 | 0.988781 | 0.005834 | 0.005385 |
| 23-P1917 | DO_MixedBreed | domestic dog | North America | Fitak et al. 2018 | 0.981251 | 0.009815 | 0.008934 |
| 24-N3674 | DO_MixedBreed | domestic dog | North America | Fitak et al. 2018 | 0.99998 | 0.00001 | 0.00001 |
| 25-N1885 | DO_MixedBreed | domestic dog | North America | Fitak et al. 2018 | 0.996872 | 0.00001 | 0.003118 |
| 26-P3671 | DO_MixedBreed | domestic dog | North America | Fitak et al. 2018 | 0.977435 | 0.013828 | 0.008737 |
| 27-N3440 | DO_MixedBreed | domestic dog | North America | Fitak et al. 2018 | 0.976916 | 0.023074 | 0.00001 |
| 28-N3661 | DO_MixedBreed | domestic dog | North America | Fitak et al. 2018 | 0.933797 | 0.060624 | 0.005579 |
| 29-N1972 | DO_MixedBreed | domestic dog | North America | Fitak et al. 2018 | 0.978936 | 0.011725 | 0.009338 |
| 30-N1915 | DO_MixedBreed | domestic dog | North America | Fitak et al. 2018 | 0.984848 | 0.005931 | 0.009221 |
| 31-P1891 | DO_MixedBreed | domestic dog | North America | Fitak et al. 2018 | 0.953892 | 0.036484 | 0.009625 |
| 32-P3668 | DO_MixedBreed | domestic dog | North America | Fitak et al. 2018 | 0.97259 | 0.0274 | 0.00001 |
| 33-P1879 | DO_MixedBreed | domestic dog | North America | Fitak et al. 2018 | 0.975485 | 0.024505 | 0.00001 |
| 34-P3663 | DO_MixedBreed | domestic dog | North America | Fitak et al. 2018 | 0.983706 | 0.011573 | 0.004721 |
| 35-N3660 | DO_MixedBreed | domestic dog | North America | Fitak et al. 2018 | 0.987654 | 0.009276 | 0.00307 |
| 36-N1963 | DO_MixedBreed | domestic dog | North America | Fitak et al. 2018 | 0.996583 | 0.00001 | 0.003407 |
| DOG-Angel | DO_Montana+Texas | domestic dog | North America | Fitak et al. 2018 | 0.991956 | 0.00001 | 0.008034 |
| 1 | DO_Montana+Texas | domestic dog | North America | Fitak et al. 2018 | 0.989119 | 0.00001 | 0.010871 |
| 126 | 1PL | domestic dog | Poland | Pilot et al. 2015 | 0.99998 | 0.00001 | 0.00001 |
| 128 | 3PL | domestic dog | Poland | Pilot et al. 2015 | 0.98587 | 0.011244 | 0.002885 |

| | | | | | | | |
|---|---|---|---|---|---|---|---|
| 132 | 7PL | domestic dog | Poland | Pilot et al. 2015 | 0.984579 | 0.009841 | 0.00558 |
| 134 | 9PL | domestic dog | Poland | Pilot et al. 2015 | 0.986631 | 0.009073 | 0.004297 |
| 136 | 13PL | domestic dog | Poland | Pilot et al. 2015 | 0.9744 | 0.019066 | 0.006534 |
| 138 | 15PL | domestic dog | Poland | Pilot et al. 2015 | 0.997473 | 0.00001 | 0.002517 |
| 140 | 17PL | domestic dog | Poland | Pilot et al. 2015 | 0.981184 | 0.011923 | 0.006894 |
| 144 | 21PL | domestic dog | Poland | Pilot et al. 2015 | 0.974412 | 0.01619 | 0.009398 |
| PortVDog | PT61.realigned | domestic dog | Portugal | Frantz et al. 2016 | 0.971575 | 0.013971 | 0.014454 |
| PortVDog | PT71.realigned | domestic dog | Portugal | Frantz et al. 2016 | 0.989038 | 0.002336 | 0.008626 |
| Asian_Breed | KM1 | domestic dog | pure breed | Frantz et al. 2016 | 0.99998 | 0.00001 | 0.00001 |
| Asian_Breed | TM1 | domestic dog | pure breed | Frantz et al. 2016 | 0.76079 | 0.211307 | 0.027903 |
| Asian_Breed | TM2 | domestic dog | pure breed | Frantz et al. 2016 | 0.755186 | 0.21862 | 0.026194 |
| Asian_Breed | TM3 | domestic dog | pure breed | Frantz et al. 2016 | 0.757936 | 0.215177 | 0.026887 |
| Asian_Breed | TM4 | domestic dog | pure breed | Frantz et al. 2016 | 0.766325 | 0.211987 | 0.021688 |
| Asian_Breed | TM5 | domestic dog | pure breed | Frantz et al. 2016 | 0.775213 | 0.191783 | 0.033004 |
| Asian_Breed | TM6 | domestic dog | pure breed | Frantz et al. 2016 | 0.766949 | 0.200447 | 0.032604 |
| Asian_Breed | TM7 | domestic dog | pure breed | Frantz et al. 2016 | 0.746498 | 0.220332 | 0.03317 |
| Asian_Breed | TM8 | domestic dog | pure breed | Frantz et al. 2016 | 0.765205 | 0.204585 | 0.030211 |
| Asian_Breed | TM9 | domestic dog | pure breed | Frantz et al. 2016 | 0.761627 | 0.211931 | 0.026442 |
| Asian_Breed | TM10 | domestic dog | pure breed | Frantz et al. 2016 | 0.761093 | 0.212978 | 0.025929 |
| 3 | 1264 | domestic dog | pure breed | Pilot et al. 2015 | 0.973178 | 0.015801 | 0.011021 |
| 9 | 1363 | domestic dog | pure breed | Pilot et al. 2015 | 0.997957 | 0.002033 | 0.00001 |
| 15 | 1357 | domestic dog | pure breed | Pilot et al. 2015 | 0.989472 | 0.010518 | 0.00001 |
| 21 | 1334 | domestic dog | pure breed | Pilot et al. 2015 | 0.747486 | 0.207866 | 0.044648 |
| 22 | 727 | domestic dog | pure breed | Pilot et al. 2015 | 0.752336 | 0.215036 | 0.032628 |
| 25 | 1326 | domestic dog | pure breed | Pilot et al. 2015 | 0.985812 | 0.00717 | 0.007017 |
| 28 | 4520 | domestic dog | pure breed | Pilot et al. 2015 | 0.996697 | 0.003293 | 0.00001 |
| 29 | 1295 | domestic dog | pure breed | Pilot et al. 2015 | 0.806839 | 0.178233 | 0.014928 |
| 30 | 1373 | domestic dog | pure breed | Pilot et al. 2015 | 0.797526 | 0.181652 | 0.020821 |
| 31 | 1420 | domestic dog | pure breed | Pilot et al. 2015 | 0.986956 | 0.007934 | 0.00511 |

| | | | | | | | |
|---|---|---|---|---|---|---|---|
| 41 | 583 | domestic dog | pure breed | Pilot et al. 2015 | 0.846389 | 0.140194 | 0.013418 |
| 42 | 725 | domestic dog | pure breed | Pilot et al. 2015 | 0.809802 | 0.166126 | 0.024072 |
| 49 | 1468 | domestic dog | pure breed | Pilot et al. 2015 | 0.986387 | 0.00001 | 0.013603 |
| 50 | 594 | domestic dog | pure breed | Pilot et al. 2015 | 0.981196 | 0.003325 | 0.015479 |
| 55 | 1307 | domestic dog | pure breed | Pilot et al. 2015 | 0.979032 | 0.012526 | 0.008442 |
| 56 | 621 | domestic dog | pure breed | Pilot et al. 2015 | 0.994195 | 0.00001 | 0.005795 |
| 59 | 1347 | domestic dog | pure breed | Pilot et al. 2015 | 0.981772 | 0.009873 | 0.008355 |
| 60 | 4552 | domestic dog | pure breed | Pilot et al. 2015 | 0.99998 | 0.00001 | 0.00001 |
| 61 | 1323 | domestic dog | pure breed | Pilot et al. 2015 | 0.995851 | 0.001184 | 0.002965 |
| 66 | 571 | domestic dog | pure breed | Pilot et al. 2015 | 0.975214 | 0.013571 | 0.011215 |
| 68 | 604 | domestic dog | pure breed | Pilot et al. 2015 | 0.991834 | 0.00001 | 0.008156 |
| 69 | 1417 | domestic dog | pure breed | Pilot et al. 2015 | 0.934827 | 0.050056 | 0.015117 |
| 70 | 1350 | domestic dog | pure breed | Pilot et al. 2015 | 0.9145 | 0.072303 | 0.013198 |
| 81 | 726 | domestic dog | pure breed | Pilot et al. 2015 | 0.988509 | 0.002564 | 0.008927 |
| 89 | 1314 | domestic dog | pure breed | Pilot et al. 2015 | 0.983217 | 0.015292 | 0.001491 |
| 90 | 283 | domestic dog | pure breed | Pilot et al. 2015 | 0.993817 | 0.00001 | 0.006173 |
| 95 | 1460 | domestic dog | pure breed | Pilot et al. 2015 | 0.992085 | 0.004848 | 0.003067 |
| 2 | 749 | domestic dog | pure breed | Pilot et al. 2015 | 0.98387 | 0.013206 | 0.002923 |
| 14 | 2774 | domestic dog | pure breed | Pilot et al. 2015 | 0.99187 | 0.007772 | 0.000358 |
| 19 | 296 | domestic dog | pure breed | Pilot et al. 2015 | 0.935747 | 0.046919 | 0.017334 |
| 20 | 2726 | domestic dog | pure breed | Pilot et al. 2015 | 0.931683 | 0.055394 | 0.012923 |
| 21 | 2743 | domestic dog | pure breed | Pilot et al. 2015 | 0.841424 | 0.143328 | 0.015249 |
| 22 | 342 | domestic dog | pure breed | Pilot et al. 2015 | 0.812973 | 0.166615 | 0.020412 |
| 27 | 295 | domestic dog | pure breed | Pilot et al. 2015 | 0.981802 | 0.01323 | 0.004968 |
| 28 | 4710 | domestic dog | pure breed | Pilot et al. 2015 | 0.980509 | 0.012581 | 0.00691 |
| 29 | 2739 | domestic dog | pure breed | Pilot et al. 2015 | 0.928175 | 0.063757 | 0.008067 |
| 30 | 2721 | domestic dog | pure breed | Pilot et al. 2015 | 0.930229 | 0.056266 | 0.013505 |
| 31 | 2761 | domestic dog | pure breed | Pilot et al. 2015 | 0.933938 | 0.053982 | 0.012079 |
| 32 | 4585 | domestic dog | pure breed | Pilot et al. 2015 | 0.998065 | 0.001925 | 0.00001 |

| | | | | | | | |
|---|---|---|---|---|---|---|---|
| 36 | 2863 | domestic dog | pure breed | Pilot et al. 2015 | 0.985206 | 0.001484 | 0.013309 |
| 40 | 2882 | domestic dog | pure breed | Pilot et al. 2015 | 0.985546 | 0.00001 | 0.014444 |
| 58 | 2957 | domestic dog | pure breed | Pilot et al. 2015 | 0.979447 | 0.018549 | 0.002004 |
| 60 | 4444 | domestic dog | pure breed | Pilot et al. 2015 | 0.981307 | 0.017311 | 0.001383 |
| 62 | 1796 | domestic dog | pure breed | Pilot et al. 2015 | 0.987359 | 0.001032 | 0.011609 |
| 85 | 3235 | domestic dog | pure breed | Pilot et al. 2015 | 0.987474 | 0.00001 | 0.012516 |
| 91 | 2630 | domestic dog | pure breed | Pilot et al. 2015 | 0.975635 | 0.011016 | 0.013348 |
| 94 | 2743 | domestic dog | pure breed | Pilot et al. 2015 | 0.99998 | 0.00001 | 0.00001 |
| Australian_Shepherd | LU1 | domestic dog | pure breed | Vaysse et al. 2011 | 0.968294 | 0.01896 | 0.012746 |
| Belgian_Tervuren | GT13 | domestic dog | pure breed | Vaysse et al. 2011 | 0.985309 | 0.006343 | 0.008347 |
| Beagle | GT9 | domestic dog | pure breed | Vaysse et al. 2011 | 0.979287 | 0.008814 | 0.011899 |
| Bernese_Mountain_Dog | GT29 | domestic dog | pure breed | Vaysse et al. 2011 | 0.99178 | 0.00821 | 0.00001 |
| Border_Collie | GT75 | domestic dog | pure breed | Vaysse et al. 2011 | 0.989642 | 0.000889 | 0.009469 |
| Border_Terrier | LU45 | domestic dog | pure breed | Vaysse et al. 2011 | 0.985206 | 0.013766 | 0.001028 |
| Boxer | LU134 | domestic dog | pure breed | Vaysse et al. 2011 | 0.901126 | 0.089625 | 0.00925 |
| Brittany_Spaniel | GT76 | domestic dog | pure breed | Vaysse et al. 2011 | 0.993602 | 0.006388 | 0.00001 |
| Chihuahua | LU136 | domestic dog | pure breed | Vaysse et al. 2011 | 0.964117 | 0.026545 | 0.009338 |
| Cavalier_King_Charles_Spaniel | GT88 | domestic dog | pure breed | Vaysse et al. 2011 | 0.986848 | 0.003573 | 0.009579 |
| Cocker_Spaniel | GT99 | domestic dog | pure breed | Vaysse et al. 2011 | 0.987407 | 0.012583 | 0.00001 |
| Czechoslovakian_Wolf_Dog | GT106 | domestic dog | pure breed | Vaysse et al. 2011 | 0.817675 | 0.168895 | 0.01343 |
| Czechoslovakian_Wolf_Dog | GT107 | domestic dog | pure breed | Vaysse et al. 2011 | 0.817477 | 0.169068 | 0.013454 |
| Czechoslovakian_Wolf_Dog | GT108 | domestic dog | pure breed | Vaysse et al. 2011 | 0.846165 | 0.146722 | 0.007112 |

| | | | | | | | |
|---|---|---|---|---|---|---|---|
| Dachshund | GT109 | domestic dog | pure breed | Vaysse et al. 2011 | 0.987587 | 0.004576 | 0.007837 |
| Dalmatian | GT127 | domestic dog | pure breed | Vaysse et al. 2011 | 0.983779 | 0.016211 | 0.00001 |
| Doberman_Pinscher | LU142 | domestic dog | pure breed | Vaysse et al. 2011 | 0.997253 | 0.002737 | 0.00001 |
| English_Bulldog | GT160 | domestic dog | pure breed | Vaysse et al. 2011 | 0.99998 | 0.00001 | 0.00001 |
| English_Bull_Terrier | GT140 | domestic dog | pure breed | Vaysse et al. 2011 | 0.99998 | 0.00001 | 0.00001 |
| Elkhound | GT139 | domestic dog | pure breed | Vaysse et al. 2011 | 0.962646 | 0.030057 | 0.007296 |
| English_Springer_Spaniel | LU598 | domestic dog | pure breed | Vaysse et al. 2011 | 0.985151 | 0.008038 | 0.006811 |
| English_Setter | GT172 | domestic dog | pure breed | Vaysse et al. 2011 | 0.989642 | 0.010348 | 0.00001 |
| Eurasian | GT173 | domestic dog | pure breed | Vaysse et al. 2011 | 0.869978 | 0.112624 | 0.017399 |
| Eurasian | GT174 | domestic dog | pure breed | Vaysse et al. 2011 | 0.848244 | 0.130487 | 0.02127 |
| Eurasian | GT175 | domestic dog | pure breed | Vaysse et al. 2011 | 0.862541 | 0.117595 | 0.019864 |
| Eurasian | GT176 | domestic dog | pure breed | Vaysse et al. 2011 | 0.865824 | 0.116165 | 0.018011 |
| Eurasian | GT177 | domestic dog | pure breed | Vaysse et al. 2011 | 0.840327 | 0.138132 | 0.021541 |
| Eurasian | GT178 | domestic dog | pure breed | Vaysse et al. 2011 | 0.850798 | 0.126551 | 0.022651 |
| Eurasian | GT179 | domestic dog | pure breed | Vaysse et al. 2011 | 0.849832 | 0.130032 | 0.020136 |
| Eurasian | GT180 | domestic dog | pure breed | Vaysse et al. 2011 | 0.835783 | 0.145244 | 0.018973 |
| Eurasian | GT181 | domestic dog | pure breed | Vaysse et al. 2011 | 0.859996 | 0.119644 | 0.02036 |
| Eurasian | GT182 | domestic dog | pure breed | Vaysse et al. 2011 | 0.823679 | 0.147406 | 0.028915 |
| Eurasian | GT183 | domestic dog | pure breed | Vaysse et al. 2011 | 0.915949 | 0.067975 | 0.016077 |
| Eurasian | GT184 | domestic | pure | Vaysse | 0.85172 | 0.136661 | 0.011619 |

| | | | | | | | |
|---|---|---|---|---|---|---|---|
| | | dog | breed | et al. 2011 | | | |
| Flatcoated_Retriever | GT198 | domestic dog | pure breed | Vaysse et al. 2011 | 0.981059 | 0.010564 | 0.008377 |
| Finnish_Spitz | GT185 | domestic dog | pure breed | Vaysse et al. 2011 | 0.959171 | 0.028979 | 0.01185 |
| Finnish_Spitz | GT186 | domestic dog | pure breed | Vaysse et al. 2011 | 0.956623 | 0.031999 | 0.011378 |
| Finnish_Spitz | GT187 | domestic dog | pure breed | Vaysse et al. 2011 | 0.958144 | 0.039337 | 0.002518 |
| Finnish_Spitz | GT188 | domestic dog | pure breed | Vaysse et al. 2011 | 0.963272 | 0.025216 | 0.011512 |
| Finnish_Spitz | GT189 | domestic dog | pure breed | Vaysse et al. 2011 | 0.96097 | 0.031873 | 0.007157 |
| Finnish_Spitz | GT190 | domestic dog | pure breed | Vaysse et al. 2011 | 0.951773 | 0.045676 | 0.002551 |
| Finnish_Spitz | GT191 | domestic dog | pure breed | Vaysse et al. 2011 | 0.956887 | 0.034777 | 0.008336 |
| Finnish_Spitz | GT192 | domestic dog | pure breed | Vaysse et al. 2011 | 0.948554 | 0.032078 | 0.019368 |
| Finnish_Spitz | GT193 | domestic dog | pure breed | Vaysse et al. 2011 | 0.95336 | 0.035861 | 0.010779 |
| Finnish_Spitz | GT194 | domestic dog | pure breed | Vaysse et al. 2011 | 0.959854 | 0.040136 | 0.00001 |
| Finnish_Spitz | GT195 | domestic dog | pure breed | Vaysse et al. 2011 | 0.954094 | 0.043035 | 0.002871 |
| Finnish_Spitz | GT196 | domestic dog | pure breed | Vaysse et al. 2011 | 0.956828 | 0.033128 | 0.010043 |
| Gordon_Setter | LU604 | domestic dog | pure breed | Vaysse et al. 2011 | 0.989455 | 0.007608 | 0.002936 |
| Greyhound | GT249 | domestic dog | pure breed | Vaysse et al. 2011 | 0.985095 | 0.014895 | 0.00001 |
| Golden_Retriever | GT212 | domestic dog | pure breed | Vaysse et al. 2011 | 0.989493 | 0.008572 | 0.001934 |
| German_Shepherd | GT211 | domestic dog | pure breed | Vaysse et al. 2011 | 0.99998 | 0.00001 | 0.00001 |
| Greenland_Sledge_Dog | GT226 | domestic dog | pure breed | Vaysse et al. 2011 | 0.784354 | 0.195126 | 0.020519 |
| Greenland_Sledge_Dog | GT227 | domestic dog | pure breed | Vaysse et al. | 0.787895 | 0.196373 | 0.015732 |

| | | | | | | | |
|---|---|---|---|---|---|---|---|
| | | | | 2011 | | | |
| Greenland_Sledge_Dog | GT228 | domestic dog | pure breed | Vaysse et al. 2011 | 0.785708 | 0.197169 | 0.017123 |
| Greenland_Sledge_Dog | GT229 | domestic dog | pure breed | Vaysse et al. 2011 | 0.783691 | 0.203754 | 0.012555 |
| Greenland_Sledge_Dog | GT230 | domestic dog | pure breed | Vaysse et al. 2011 | 0.783709 | 0.201173 | 0.015119 |
| Greenland_Sledge_Dog | GT231 | domestic dog | pure breed | Vaysse et al. 2011 | 0.777058 | 0.205683 | 0.017259 |
| Greenland_Sledge_Dog | GT232 | domestic dog | pure breed | Vaysse et al. 2011 | 0.794118 | 0.182549 | 0.023334 |
| Greenland_Sledge_Dog | GT233 | domestic dog | pure breed | Vaysse et al. 2011 | 0.79791 | 0.194888 | 0.007202 |
| Greenland_Sledge_Dog | GT234 | domestic dog | pure breed | Vaysse et al. 2011 | 0.786364 | 0.201154 | 0.012482 |
| Greenland_Sledge_Dog | GT235 | domestic dog | pure breed | Vaysse et al. 2011 | 0.781993 | 0.206275 | 0.011732 |
| Greenland_Sledge_Dog | GT236 | domestic dog | pure breed | Vaysse et al. 2011 | 0.777576 | 0.206744 | 0.01568 |
| Greenland_Sledge_Dog | GT237 | domestic dog | pure breed | Vaysse et al. 2011 | 0.787313 | 0.19513 | 0.017558 |
| Siberian_Husky | GT403 | domestic dog | pure breed | Vaysse et al. 2011 | 0.804015 | 0.16628 | 0.029705 |
| Siberian_Husky | GT405 | domestic dog | pure breed | Vaysse et al. 2011 | 0.799567 | 0.174996 | 0.025437 |
| Irish_Wolfhound | LU1210 | domestic dog | pure breed | Vaysse et al. 2011 | 0.983683 | 0.010555 | 0.005762 |
| Jack_Russell_Terrier | GT263 | domestic dog | pure breed | Vaysse et al. 2011 | 0.985004 | 0.014122 | 0.000874 |
| Large_Munsterlander | GT279 | domestic dog | pure breed | Vaysse et al. 2011 | 0.978925 | 0.01905 | 0.002025 |
| Labrador_Retriever | GT264 | domestic dog | pure breed | Vaysse et al. 2011 | 0.99454 | 0.00545 | 0.00001 |
| Mops | GT282 | domestic dog | pure breed | Vaysse et al. 2011 | 0.983802 | 0.015377 | 0.000821 |
| Newfoundland | LU1375 | domestic dog | pure breed | Vaysse et al. 2011 | 0.985819 | 0.006287 | 0.007894 |
| NSDTR | GT310 | domestic dog | pure breed | Vaysse et al. 2011 | 0.980681 | 0.015072 | 0.004247 |

| | | | | | | | |
|---|---|---|---|---|---|---|---|
| Rottweiler | GT357 | domestic dog | pure breed | Vaysse et al. 2011 | 0.985883 | 0.012025 | 0.002092 |
| Samoyed | GT401 | domestic dog | pure breed | Vaysse et al. 2011 | 0.890945 | 0.087886 | 0.021168 |
| Samoyed | GT402 | domestic dog | pure breed | Vaysse et al. 2011 | 0.892333 | 0.084454 | 0.023213 |
| Sarloos | LU1453 | domestic dog | pure breed | Vaysse et al. 2011 | 0.754344 | 0.230743 | 0.014913 |
| Sarloos | LU1454 | domestic dog | pure breed | Vaysse et al. 2011 | 0.610284 | 0.347732 | 0.041984 |
| Schipperke | LU1455 | domestic dog | pure breed | Vaysse et al. 2011 | 0.97902 | 0.02058 | 0.0004 |
| Schnauzer | LU1565 | domestic dog | pure breed | Vaysse et al. 2011 | 0.980578 | 0.010846 | 0.008576 |
| Shar_Pei | GT358 | domestic dog | pure breed | Vaysse et al. 2011 | 0.744415 | 0.214983 | 0.040602 |
| Shar_Pei | GT359 | domestic dog | pure breed | Vaysse et al. 2011 | 0.738374 | 0.22246 | 0.039166 |
| Shar_Pei | GT361 | domestic dog | pure breed | Vaysse et al. 2011 | 0.734954 | 0.218878 | 0.046168 |
| Shar_Pei | GT362 | domestic dog | pure breed | Vaysse et al. 2011 | 0.82736 | 0.136981 | 0.03566 |
| Shar_Pei | GT363 | domestic dog | pure breed | Vaysse et al. 2011 | 0.728972 | 0.237703 | 0.033324 |
| Shar_Pei | GT364 | domestic dog | pure breed | Vaysse et al. 2011 | 0.753934 | 0.201158 | 0.044907 |
| Shar_Pei | GT365 | domestic dog | pure breed | Vaysse et al. 2011 | 0.740203 | 0.219722 | 0.040075 |
| Shar_Pei | GT366 | domestic dog | pure breed | Vaysse et al. 2011 | 0.756583 | 0.201562 | 0.041855 |
| Shar_Pei | GT367 | domestic dog | pure breed | Vaysse et al. 2011 | 0.739712 | 0.222237 | 0.038051 |
| Shar_Pei | GT368 | domestic dog | pure breed | Vaysse et al. 2011 | 0.735719 | 0.223551 | 0.04073 |
| Shar_Pei | GT369 | domestic dog | pure breed | Vaysse et al. 2011 | 0.735452 | 0.226389 | 0.038159 |
| Standard_Poodle | GT370 | domestic dog | pure breed | Vaysse et al. 2011 | 0.962722 | 0.026319 | 0.010959 |
| Ter | GT387 | domestic | pure | Vaysse | 0.943494 | 0.03605 | 0.020456 |

| | | | | | | | |
|---|---|---|---|---|---|---|---|
| | | dog | breed | et al. 2011 | | | |
| Yorkshire_Terrier | GT388 | domestic dog | pure breed | Vaysse et al. 2011 | 0.989591 | 0.002524 | 0.007885 |
| Weimaraner | GT433 | domestic dog | pure breed | Vaysse et al. 2011 | 0.988045 | 0.00001 | 0.011945 |
| 133 | 8284 | domestic dog | Saudi Arabia | New Dataset | 0.806506 | 0.158154 | 0.035341 |
| 106 | 2AS | domestic dog | Saudi Arabia | Pilot et al. 2015 | 0.824026 | 0.145164 | 0.03081 |
| 107 | 10AS | domestic dog | Saudi Arabia | Pilot et al. 2015 | 0.832423 | 0.138094 | 0.029482 |
| 109 | 13AS | domestic dog | Saudi Arabia | Pilot et al. 2015 | 0.825098 | 0.157954 | 0.016949 |
| 111 | 15AS | domestic dog | Saudi Arabia | Pilot et al. 2015 | 0.82561 | 0.15752 | 0.016871 |
| 115 | 19AS | domestic dog | Saudi Arabia | Pilot et al. 2015 | 0.827461 | 0.155782 | 0.016758 |
| 116 | 20AS | domestic dog | Saudi Arabia | Pilot et al. 2015 | 0.84352 | 0.134994 | 0.021486 |
| 118 | 24AS | domestic dog | Saudi Arabia | Pilot et al. 2015 | 0.86825 | 0.105838 | 0.025912 |
| 121 | 28AS | domestic dog | Saudi Arabia | Pilot et al. 2015 | 0.831523 | 0.142741 | 0.025737 |
| 125 | 32AS | domestic dog | Saudi Arabia | Pilot et al. 2015 | 0.891312 | 0.093707 | 0.014981 |
| 93 | 2SL | domestic dog | Slovenia | Pilot et al. 2015 | 0.971922 | 0.014796 | 0.013282 |
| 96 | 5SL | domestic dog | Slovenia | Pilot et al. 2015 | 0.979578 | 0.015527 | 0.004894 |
| 97 | 8SL | domestic dog | Slovenia | Pilot et al. 2015 | 0.992909 | 0.005632 | 0.001459 |
| 98 | 11SL | domestic dog | Slovenia | Pilot et al. 2015 | 0.977024 | 0.022562 | 0.000414 |
| 99 | 6SL | domestic dog | Slovenia | Pilot et al. 2015 | 0.99998 | 0.00001 | 0.00001 |
| 102 | 10SL | domestic dog | Slovenia | Pilot et al. 2015 | 0.990706 | 0.00001 | 0.009284 |
| 103 | 12SL | domestic dog | Slovenia | Pilot et al. 2015 | 0.982865 | 0.016777 | 0.000358 |
| 105 | 14SL | domestic dog | Slovenia | Pilot et al. 2015 | 0.965927 | 0.029727 | 0.004346 |
| 157 | 1TAJ | domestic dog | Thailand | Pilot et al. 2015 | 0.745891 | 0.226816 | 0.027293 |
| 167 | 3TAJ | domestic dog | Thailand | Pilot et al. 2015 | 0.903314 | 0.086249 | 0.010437 |
| 169 | 5TAJ | domestic dog | Thailand | Pilot et al. 2015 | 0.852599 | 0.1246 | 0.022802 |
| 171 | 7TAJ | domestic dog | Thailand | Pilot et al. 2015 | 0.787644 | 0.178608 | 0.033747 |
| 173 | 10TAJ | domestic dog | Thailand | Pilot et al. 2015 | 0.778627 | 0.180057 | 0.041316 |
| 213 | 11TAJ | domestic dog | Thailand | Pilot et al. 2015 | 0.80755 | 0.166222 | 0.026228 |
| 215 | 13TAJ | domestic dog | Thailand | Pilot et al. 2015 | 0.905741 | 0.076749 | 0.017511 |

| | | | | | | | |
|---|---|---|---|---|---|---|---|
| 219 | 18TAJ | domestic dog | Thailand | Pilot et al. 2015 | 0.807967 | 0.152699 | 0.039334 |
| 221 | 21TAJ | domestic dog | Thailand | Pilot et al. 2015 | 0.954218 | 0.031411 | 0.014371 |
| 223 | 23TAJ | domestic dog | Thailand | Pilot et al. 2015 | 0.9923 | 0.004142 | 0.003558 |
| 49 | 1 | domestic dog | West Asia | Pilot et al. 2015 | 0.864276 | 0.108507 | 0.027217 |
| 50 | 2 | domestic dog | West Asia | Pilot et al. 2015 | 0.872096 | 0.096017 | 0.031887 |
| 51 | 3 | domestic dog | West Asia | Pilot et al. 2015 | 0.873831 | 0.096368 | 0.029801 |
| 52 | 4 | domestic dog | West Asia | Pilot et al. 2015 | 0.848312 | 0.126642 | 0.025046 |
| 53 | 5 | domestic dog | West Asia | Pilot et al. 2015 | 0.877857 | 0.092833 | 0.02931 |
| 54 | 6 | domestic dog | West Asia | Pilot et al. 2015 | 0.869176 | 0.096271 | 0.034553 |
| 55 | 7 | domestic dog | West Asia | Pilot et al. 2015 | 0.886246 | 0.094492 | 0.019261 |
| 56 | 8 | domestic dog | West Asia | Pilot et al. 2015 | 0.956265 | 0.038263 | 0.005472 |
| 29 | 103 | golden jackal | Bulgaria | New Dataset | 0.00001 | 0.00001 | 0.99998 |
| 33 | 104 | golden jackal | Bulgaria | New Dataset | 0.00001 | 0.00001 | 0.99998 |
| 37 | 106 | golden jackal | Bulgaria | New Dataset | 0.00001 | 0.00001 | 0.99998 |
| 30 | 7180 | golden jackal | Caucasus | Pilot et al. 2015 | 0.00001 | 0.00001 | 0.99998 |
| 189 | 5734 | golden jackal | Caucasus | New Dataset | 0.00001 | 0.00001 | 0.99998 |
| 190 | 7184 | golden jackal | Caucasus | New Dataset | 0.00001 | 0.00001 | 0.99998 |
| 191 | 8341 | golden jackal | Caucasus | New Dataset | 0.051855 | 0.00001 | 0.948135 |
| Jackal | Jackal | golden jackal | Eurasia | Frantz et al. 2016 | 0.00001 | 0.00001 | 0.99998 |
| BC-WOLF-01 | WO_BritishColumbiaCanada | grey wolf | British Columbia, Canada | Fitak et al. 2018 | 0.00001 | 0.957239 | 0.042751 |
| BC-WOLF-05 | WO_BritishColumbiaCanada | grey wolf | British Columbia, Canada | Fitak et al. 2018 | 0.00001 | 0.969033 | 0.030957 |
| BC-WOLF-09 | WO_BritishColumbiaCanada | grey wolf | British Columbia, Canada | Fitak et al. 2018 | 0.00001 | 0.955721 | 0.044269 |
| BC-WOLF-13 | WO_BritishColumbiaCanada | grey wolf | British Columbia, Canada | Fitak et al. 2018 | 0.135204 | 0.835427 | 0.029369 |
| BC-WOLF-15 | WO_BritishColumbiaCanada | grey wolf | British Columbia, Canada | Fitak et al. 2018 | 0.00001 | 0.959483 | 0.040507 |
| BC-WOLF-17 | WO_BritishColumbiaCanada | grey wolf | British Columbia, Canada | Fitak et al. 2018 | 0.00001 | 0.948765 | 0.051225 |
| BC-WOLF-19 | WO_BritishColumbiaCanada | grey wolf | British Columbia, | Fitak et al. 2018 | 0.00001 | 0.95602 | 0.04397 |

| | | | | | | | |
|---|---|---|---|---|---|---|---|
| | | | Canada | | | | |
| BC-WOLF-21 | WO_BritishColumbiaCanada | grey wolf | British Columbia, Canada | Fitak et al. 2018 | 0.00001 | 0.962539 | 0.037451 |
| BC-WOLF-23 | WO_BritishColumbiaCanada | grey wolf | British Columbia, Canada | Fitak et al. 2018 | 0.00001 | 0.965044 | 0.034946 |
| BC-WOLF-25 | WO_BritishColumbiaCanada | grey wolf | British Columbia, Canada | Fitak et al. 2018 | 0.00001 | 0.966494 | 0.033496 |
| BC-WOLF-27 | WO_BritishColumbiaCanada | grey wolf | British Columbia, Canada | Fitak et al. 2018 | 0.00001 | 0.966418 | 0.033572 |
| BC-WOLF-29 | WO_BritishColumbiaCanada | grey wolf | British Columbia, Canada | Fitak et al. 2018 | 0.00001 | 0.948642 | 0.051348 |
| BC-WOLF-31 | WO_BritishColumbiaCanada | grey wolf | British Columbia, Canada | Fitak et al. 2018 | 0.00001 | 0.95258 | 0.04741 |
| 175 | WO_BritishColumbiaCanada | grey wolf | British Columbia, Canada | Fitak et al. 2018 | 0.00001 | 0.955753 | 0.044237 |
| 177 | WO_BritishColumbiaCanada | grey wolf | British Columbia, Canada | Fitak et al. 2018 | 0.00001 | 0.949497 | 0.050493 |
| 2 | 117 | grey wolf | Bulgaria | New Dataset | 0.020205 | 0.979785 | 0.00001 |
| 3 | 186 | grey wolf | Bulgaria | New Dataset | 0.016555 | 0.983435 | 0.00001 |
| 4 | 211 | grey wolf | Bulgaria | New Dataset | 0.027692 | 0.972298 | 0.00001 |
| 5 | 15 | grey wolf | Bulgaria | New Dataset | 0.078835 | 0.921155 | 0.00001 |
| 7 | 187 | grey wolf | Bulgaria | New Dataset | 0.022002 | 0.977988 | 0.00001 |
| 8 | 213 | grey wolf | Bulgaria | New Dataset | 0.021604 | 0.978386 | 0.00001 |
| 9 | 47 | grey wolf | Bulgaria | New Dataset | 0.015984 | 0.984006 | 0.00001 |
| 10 | 162 | grey wolf | Bulgaria | New Dataset | 0.028829 | 0.971161 | 0.00001 |
| 11 | 188 | grey wolf | Bulgaria | New Dataset | 0.027379 | 0.972611 | 0.00001 |
| 12 | 214 | grey wolf | Bulgaria | New Dataset | 0.023821 | 0.976169 | 0.00001 |
| 13 | 60 | grey wolf | Bulgaria | New Dataset | 0.008834 | 0.991156 | 0.00001 |
| 14 | 166 | grey wolf | Bulgaria | New Dataset | 0.023466 | 0.976524 | 0.00001 |
| 15 | 191 | grey wolf | Bulgaria | New Dataset | 0.010829 | 0.989161 | 0.00001 |
| 16 | 215 | grey wolf | Bulgaria | New Dataset | 0.038879 | 0.961111 | 0.00001 |
| 18 | 170 | grey wolf | Bulgaria | New Dataset | 0.011832 | 0.988158 | 0.00001 |
| 19 | 192 | grey wolf | Bulgaria | New Dataset | 0.01717 | 0.98282 | 0.00001 |
| 20 | 216 | grey | Bulgaria | New | 0.019676 | 0.980314 | 0.00001 |

| | | | | | | | |
|---|---|---|---|---|---|---|---|
| | | wolf | | Dataset | | | |
| 21 | 78 | grey wolf | Bulgaria | New Dataset | 0.024539 | 0.975451 | 0.00001 |
| 23 | 198 | grey wolf | Bulgaria | New Dataset | 0.027611 | 0.972379 | 0.00001 |
| 24 | 217 | grey wolf | Bulgaria | New Dataset | 0.024377 | 0.975613 | 0.00001 |
| 25 | 89 | grey wolf | Bulgaria | New Dataset | 0.019503 | 0.980487 | 0.00001 |
| 26 | 172 | grey wolf | Bulgaria | New Dataset | 0.015464 | 0.984526 | 0.00001 |
| 27 | 200 | grey wolf | Bulgaria | New Dataset | 0.011918 | 0.988072 | 0.00001 |
| 28 | 222 | grey wolf | Bulgaria | New Dataset | 0.023736 | 0.976254 | 0.00001 |
| 30 | 178 | grey wolf | Bulgaria | New Dataset | 0.026283 | 0.973707 | 0.00001 |
| 32 | 224 | grey wolf | Bulgaria | New Dataset | 0.014518 | 0.985472 | 0.00001 |
| 34 | 180 | grey wolf | Bulgaria | New Dataset | 0.065557 | 0.934433 | 0.00001 |
| 36 | 225 | grey wolf | Bulgaria | New Dataset | 0.028537 | 0.971453 | 0.00001 |
| 38 | 181 | grey wolf | Bulgaria | New Dataset | 0.021493 | 0.978497 | 0.00001 |
| 39 | 204 | grey wolf | Bulgaria | New Dataset | 0.016192 | 0.983798 | 0.00001 |
| 40 | 228 | grey wolf | Bulgaria | New Dataset | 0.015314 | 0.984676 | 0.00001 |
| 42 | 182 | grey wolf | Bulgaria | New Dataset | 0.026791 | 0.973199 | 0.00001 |
| 43 | 206 | grey wolf | Bulgaria | New Dataset | 0.026309 | 0.973681 | 0.00001 |
| 44 | 230 | grey wolf | Bulgaria | New Dataset | 0.024565 | 0.975425 | 0.00001 |
| 45 | 114 | grey wolf | Bulgaria | New Dataset | 0.023444 | 0.976546 | 0.00001 |
| 46 | 185 | grey wolf | Bulgaria | New Dataset | 0.019258 | 0.980732 | 0.00001 |
| 47 | 207 | grey wolf | Bulgaria | New Dataset | 0.036477 | 0.963513 | 0.00001 |
| 48 | 233 | grey wolf | Bulgaria | New Dataset | 0.054169 | 0.945821 | 0.00001 |
| 36 | 5796 | grey wolf | Caucasus | Pilot et al. 2015 | 0.055953 | 0.884944 | 0.059103 |
| 42 | 5798 | grey wolf | Caucasus | Pilot et al. 2015 | 0.053679 | 0.88891 | 0.057411 |
| 48 | 5795 | grey wolf | Caucasus | Pilot et al. 2015 | 0.064039 | 0.874228 | 0.061732 |
| 98 | KTa | grey wolf | Caucasus | New Dataset | 0.039591 | 0.903452 | 0.056957 |
| 99 | W01 | grey wolf | Caucasus | New Dataset | 0.053585 | 0.885688 | 0.060727 |
| 100 | W04 | grey wolf | Caucasus | New Dataset | 0.050758 | 0.893804 | 0.055439 |
| 101 | W05 | grey wolf | Caucasus | New Dataset | 0.041328 | 0.899568 | 0.059104 |
| 102 | W07 | grey | Caucasus | New | 0.052258 | 0.887502 | 0.06024 |

| | | | | | | | |
|---|---|---|---|---|---|---|---|
| | | wolf | | Dataset | | | |
| 103 | W11 | grey wolf | Caucasus | New Dataset | 0.04575 | 0.895351 | 0.058898 |
| 104 | W10 | grey wolf | Caucasus | New Dataset | 0.056668 | 0.883068 | 0.060264 |
| 105 | WA | grey wolf | Caucasus | New Dataset | 0.053718 | 0.874781 | 0.071501 |
| 106 | WGJ | grey wolf | Caucasus | New Dataset | 0.046339 | 0.894927 | 0.058734 |
| 107 | WKG | grey wolf | Caucasus | New Dataset | 0.053642 | 0.874535 | 0.071822 |
| 108 | WUN | grey wolf | Caucasus | New Dataset | 0.056601 | 0.88568 | 0.057719 |
| 134 | 5282 | grey wolf | Caucasus | New Dataset | 0.042556 | 0.907414 | 0.05003 |
| 135 | 5283 | grey wolf | Caucasus | New Dataset | 0.047443 | 0.897656 | 0.054901 |
| 136 | 5285 | grey wolf | Caucasus | New Dataset | 0.051252 | 0.88109 | 0.067658 |
| 137 | 5286 | grey wolf | Caucasus | New Dataset | 0.051218 | 0.889409 | 0.059373 |
| 138 | 5289 | grey wolf | Caucasus | New Dataset | 0.042981 | 0.894733 | 0.062285 |
| 139 | 5324 | grey wolf | Caucasus | New Dataset | 0.050236 | 0.897333 | 0.052431 |
| 140 | 5325 | grey wolf | Caucasus | New Dataset | 0.047411 | 0.897714 | 0.054875 |
| 141 | 5326 | grey wolf | Caucasus | New Dataset | 0.054402 | 0.883791 | 0.061807 |
| 142 | 5328 | grey wolf | Caucasus | New Dataset | 0.051793 | 0.895775 | 0.052431 |
| 143 | 5329 | grey wolf | Caucasus | New Dataset | 0.055064 | 0.881246 | 0.06369 |
| 144 | 5330 | grey wolf | Caucasus | New Dataset | 0.044939 | 0.89613 | 0.058931 |
| 145 | 5331 | grey wolf | Caucasus | New Dataset | 0.05734 | 0.886713 | 0.055948 |
| 146 | 5332 | grey wolf | Caucasus | New Dataset | 0.061956 | 0.878572 | 0.059472 |
| 147 | 5333 | grey wolf | Caucasus | New Dataset | 0.04302 | 0.89489 | 0.06209 |
| 148 | 5334 | grey wolf | Caucasus | New Dataset | 0.052222 | 0.886832 | 0.060946 |
| Wolf | 19785 | grey wolf | Eurasia | Vaysse et al. 2011 | 0.016195 | 0.975345 | 0.00846 |
| Wolf | 22799 | grey wolf | Eurasia | Vaysse et al. 2011 | 0.045897 | 0.954093 | 0.00001 |
| Wolf | 22800 | grey wolf | Eurasia | Vaysse et al. 2011 | 0.039013 | 0.960633 | 0.000354 |
| Wolf | 22802 | grey wolf | Eurasia | Vaysse et al. 2011 | 0.016738 | 0.980612 | 0.00265 |
| Wolf | 22803 | grey wolf | Eurasia | Vaysse et al. 2011 | 0.014042 | 0.98472 | 0.001237 |

| | | | | | | | |
|---|---|---|---|---|---|---|---|
| Wolf | 22809 | grey wolf | Eurasia | Vaysse et al. 2011 | 0.030994 | 0.968996 | 0.00001 |
| Wolf | 22810 | grey wolf | Eurasia | Vaysse et al. 2011 | 0.030851 | 0.969139 | 0.00001 |
| Wolf | LU1655 | grey wolf | Eurasia | Vaysse et al. 2011 | 0.038332 | 0.961658 | 0.00001 |
| Wolf | LU1656 | grey wolf | Eurasia | Vaysse et al. 2011 | 0.020075 | 0.978029 | 0.001896 |
| Wolf | LU1657 | grey wolf | Eurasia | Vaysse et al. 2011 | 0.029095 | 0.970895 | 0.00001 |
| Wolf | LUb1 | grey wolf | Eurasia | Vaysse et al. 2011 | 0.046488 | 0.943401 | 0.010111 |
| Wolf | LUb2 | grey wolf | Eurasia | Vaysse et al. 2011 | 0.02924 | 0.97075 | 0.00001 |
| 2 | 5 | grey wolf | Europe | Stronen et al. 2015 | 0.058534 | 0.938616 | 0.00285 |
| 9 | 27 | grey wolf | Europe | Stronen et al. 2015 | 0.049231 | 0.948078 | 0.002691 |
| 10 | 52 | grey wolf | Europe | Stronen et al. 2015 | 0.018871 | 0.974086 | 0.007044 |
| 12 | 57 | grey wolf | Europe | Stronen et al. 2015 | 0.021797 | 0.978193 | 0.00001 |
| 13 | 62 | grey wolf | Europe | Stronen et al. 2015 | 0.014252 | 0.980056 | 0.005692 |
| 18 | 103 | grey wolf | Europe | Stronen et al. 2015 | 0.035379 | 0.951676 | 0.012945 |
| 21 | 112 | grey wolf | Europe | Stronen et al. 2015 | 0.040401 | 0.95434 | 0.005259 |
| 24 | 128 | grey wolf | Europe | Stronen et al. 2015 | 0.097909 | 0.902081 | 0.00001 |
| 25 | 132 | grey wolf | Europe | Stronen et al. 2015 | 0.020771 | 0.979219 | 0.00001 |
| 26 | 133 | grey wolf | Europe | Stronen et al. 2015 | 0.032602 | 0.962337 | 0.00506 |
| 29 | 163 | grey wolf | Europe | Stronen et al. 2015 | 0.047813 | 0.948353 | 0.003834 |
| 34 | 203 | grey wolf | Europe | Stronen et al. 2015 | 0.039643 | 0.960347 | 0.00001 |
| 35 | 206 | grey | Europe | Stronen | 0.026534 | 0.962149 | 0.011317 |

| | | | | | | | |
|---|---|---|---|---|---|---|---|
| | | wolf | | et al. 2015 | | | |
| 44 | 378 | grey wolf | Europe | Stronen et al. 2015 | 0.121053 | 0.867024 | 0.011923 |
| 50 | 424 | grey wolf | Europe | Stronen et al. 2015 | 0.034169 | 0.965821 | 0.00001 |
| 52 | 444 | grey wolf | Europe | Stronen et al. 2015 | 0.039879 | 0.960111 | 0.00001 |
| 57 | 527 | grey wolf | Europe | Stronen et al. 2015 | 0.050165 | 0.949825 | 0.00001 |
| 66 | 548 | grey wolf | Europe | Stronen et al. 2015 | 0.03173 | 0.96826 | 0.00001 |
| 71 | 555 | grey wolf | Europe | Stronen et al. 2015 | 0.127461 | 0.872529 | 0.00001 |
| 73 | 560 | grey wolf | Europe | Stronen et al. 2015 | 0.109771 | 0.888277 | 0.001952 |
| 74 | 562 | grey wolf | Europe | Stronen et al. 2015 | 0.117897 | 0.865216 | 0.016887 |
| 75 | 563 | grey wolf | Europe | Stronen et al. 2015 | 0.081176 | 0.902599 | 0.016226 |
| 76 | 564 | grey wolf | Europe | Stronen et al. 2015 | 0.141865 | 0.850991 | 0.007144 |
| 82 | 689 | grey wolf | Europe | Stronen et al. 2015 | 0.052361 | 0.947433 | 0.000206 |
| 84 | 846 | grey wolf | Europe | Stronen et al. 2015 | 0.094276 | 0.90389 | 0.001834 |
| 86 | 987 | grey wolf | Europe | Stronen et al. 2015 | 0.0251 | 0.97308 | 0.00182 |
| 87 | 988 | grey wolf | Europe | Stronen et al. 2015 | 0.063018 | 0.934808 | 0.002175 |
| 91 | 1003 | grey wolf | Europe | Stronen et al. 2015 | 0.068226 | 0.931764 | 0.00001 |
| 99 | 1135 | grey wolf | Europe | Stronen et al. 2015 | 0.026297 | 0.973693 | 0.00001 |
| 103 | 1148 | grey wolf | Europe | Stronen et al. 2015 | 0.025759 | 0.974166 | 0.000076 |
| 104 | 1150 | grey wolf | Europe | Stronen et al. 2015 | 0.026228 | 0.972458 | 0.001313 |
| 109 | 1174 | grey wolf | Europe | Stronen et al. | 0.045325 | 0.954665 | 0.00001 |

| | | | | | | | |
|---|---|---|---|---|---|---|---|
| | | | | 2015 | | | |
| 111 | 1219 | grey wolf | Europe | Stronen et al. 2015 | 0.106458 | 0.883622 | 0.009921 |
| 116 | 1262 | grey wolf | Europe | Stronen et al. 2015 | 0.018852 | 0.968654 | 0.012495 |
| 117 | 1263 | grey wolf | Europe | Stronen et al. 2015 | 0.028454 | 0.967376 | 0.00417 |
| 120 | 1267 | grey wolf | Europe | Stronen et al. 2015 | 0.024577 | 0.960918 | 0.014505 |
| 122 | 1271 | grey wolf | Europe | Stronen et al. 2015 | 0.019225 | 0.976283 | 0.004492 |
| 123 | 1282 | grey wolf | Europe | Stronen et al. 2015 | 0.03009 | 0.967527 | 0.002382 |
| 126 | 1344 | grey wolf | Europe | Stronen et al. 2015 | 0.103446 | 0.882655 | 0.013899 |
| 127 | 1345 | grey wolf | Europe | Stronen et al. 2015 | 0.069082 | 0.910094 | 0.020824 |
| 128 | 1355 | grey wolf | Europe | Stronen et al. 2015 | 0.263558 | 0.728262 | 0.008179 |
| 129 | 1360 | grey wolf | Europe | Stronen et al. 2015 | 0.211488 | 0.788502 | 0.00001 |
| 130 | 1367 | grey wolf | Europe | Stronen et al. 2015 | 0.197037 | 0.802953 | 0.00001 |
| 131 | 1370 | grey wolf | Europe | Stronen et al. 2015 | 0.119452 | 0.877513 | 0.003035 |
| 137 | 1382 | grey wolf | Europe | Stronen et al. 2015 | 0.029893 | 0.970097 | 0.00001 |
| 142 | 1387 | grey wolf | Europe | Stronen et al. 2015 | 0.016225 | 0.983765 | 0.00001 |
| 148 | 895 | grey wolf | Europe | Stronen et al. 2015 | 0.022448 | 0.977542 | 0.00001 |
| 149 | 1032 | grey wolf | Europe | Stronen et al. 2015 | 0.017587 | 0.982403 | 0.00001 |
| 150 | 1173 | grey wolf | Europe | Stronen et al. 2015 | 0.162507 | 0.831631 | 0.005862 |
| 153 | 1224 | grey wolf | Europe | Stronen et al. 2015 | 0.020455 | 0.978738 | 0.000806 |
| 154 | 833 | grey wolf | Europe | Stronen et al. 2015 | 0.033722 | 0.966268 | 0.00001 |

| | | | | | | | |
|---|---|---|---|---|---|---|---|
| 156 | 897 | grey wolf | Europe | Stronen et al. 2015 | 0.05213 | 0.944328 | 0.003542 |
| 159 | 1381 | grey wolf | Europe | Stronen et al. 2015 | 0.045502 | 0.9476 | 0.006897 |
| 160 | 574 | grey wolf | Europe | Stronen et al. 2015 | 0.042572 | 0.951313 | 0.006116 |
| 162 | 584 | grey wolf | Europe | Stronen et al. 2015 | 0.018201 | 0.979794 | 0.002005 |
| 165 | 531 | grey wolf | Europe | Stronen et al. 2015 | 0.008605 | 0.988566 | 0.002829 |
| 168 | 553 | grey wolf | Europe | Stronen et al. 2015 | 0.07493 | 0.92506 | 0.00001 |
| 171 | 347 | grey wolf | Europe | Stronen et al. 2015 | 0.019299 | 0.980691 | 0.00001 |
| ID-1 | WO_Idaho | grey wolf | Idaho | Fitak et al. 2018 | 0.00001 | 0.964286 | 0.035704 |
| ID-10 | WO_Idaho | grey wolf | Idaho | Fitak et al. 2018 | 0.00001 | 0.954106 | 0.045884 |
| ID-12 | WO_Idaho | grey wolf | Idaho | Fitak et al. 2018 | 0.00001 | 0.962093 | 0.037897 |
| ID-14 | WO_Idaho | grey wolf | Idaho | Fitak et al. 2018 | 0.587963 | 0.372452 | 0.039585 |
| ID-17 | WO_Idaho | grey wolf | Idaho | Fitak et al. 2018 | 0.00001 | 0.953895 | 0.046095 |
| ID-18 | WO_Idaho | grey wolf | Idaho | Fitak et al. 2018 | 0.00001 | 0.962102 | 0.037888 |
| ID-19 | WO_Idaho | grey wolf | Idaho | Fitak et al. 2018 | 0.00001 | 0.96115 | 0.03884 |
| ID-2 | WO_Idaho | grey wolf | Idaho | Fitak et al. 2018 | 0.00001 | 0.954465 | 0.045525 |
| ID-20 | WO_Idaho | grey wolf | Idaho | Fitak et al. 2018 | 0.00001 | 0.962174 | 0.037816 |
| ID-21 | WO_Idaho | grey wolf | Idaho | Fitak et al. 2018 | 0.00001 | 0.954415 | 0.045575 |
| ID-23 | WO_Idaho | grey wolf | Idaho | Fitak et al. 2018 | 0.00001 | 0.960548 | 0.039442 |
| ID-4 | WO_Idaho | grey wolf | Idaho | Fitak et al. 2018 | 0.00001 | 0.963351 | 0.036639 |
| ID-5 | WO_Idaho | grey wolf | Idaho | Fitak et al. 2018 | 0.00001 | 0.983821 | 0.016169 |
| ID-7 | WO_Idaho | grey wolf | Idaho | Fitak et al. 2018 | 0.00001 | 0.96628 | 0.03371 |
| ID-9 | WO_Idaho | grey wolf | Idaho | Fitak et al. 2018 | 0.00001 | 0.97128 | 0.02871 |
| INTAK-01 | WO_InteriorAlaska | grey wolf | Idaho | Fitak et al. 2018 | 0.00001 | 0.952923 | 0.047067 |
| INTAK-05 | WO_InteriorAlaska | grey wolf | Interior Alaska | Fitak et al. 2018 | 0.00001 | 0.966153 | 0.033837 |
| INTAK-09 | WO_InteriorAlaska | grey wolf | Interior Alaska | Fitak et al. 2018 | 0.00001 | 0.97703 | 0.02296 |
| INTAK-11 | WO_InteriorAlaska | grey | Interior | Fitak et | 0.00001 | 0.96693 | 0.03306 |

| | | | | | | | |
|---|---|---|---|---|---|---|---|
| | | wolf | Alaska | al. 2018 | | | |
| INTAK-15 | WO_InteriorAlaska | grey wolf | Interior Alaska | Fitak et al. 2018 | 0.00001 | 0.976743 | 0.023247 |
| INTAK-20 | WO_InteriorAlaska | grey wolf | Interior Alaska | Fitak et al. 2018 | 0.00001 | 0.983113 | 0.016877 |
| INTAK-24 | WO_InteriorAlaska | grey wolf | Interior Alaska | Fitak et al. 2018 | 0.00001 | 0.979959 | 0.020031 |
| 186 | WO_InteriorAlaska | grey wolf | Interior Alaska | Fitak et al. 2018 | 0.00001 | 0.946631 | 0.053359 |
| 207 | WO_InteriorAlaska | grey wolf | Interior Alaska | Fitak et al. 2018 | 0.00001 | 0.983611 | 0.016379 |
| 209 | WO_InteriorAlaska | grey wolf | Interior Alaska | Fitak et al. 2018 | 0.00001 | 0.98685 | 0.01314 |
| 212 | WO_InteriorAlaska | grey wolf | Interior Alaska | Fitak et al. 2018 | 0.00001 | 0.986255 | 0.013735 |
| 214 | WO_InteriorAlaska | grey wolf | Interior Alaska | Fitak et al. 2018 | 0.00001 | 0.977428 | 0.022562 |
| 217 | WO_InteriorAlaska | grey wolf | Interior Alaska | Fitak et al. 2018 | 0.00001 | 0.971161 | 0.028829 |
| 221 | WO_InteriorAlaska | grey wolf | Interior Alaska | Fitak et al. 2018 | 0.00001 | 0.97682 | 0.02317 |
| 225 | WO_InteriorAlaska | grey wolf | Interior Alaska | Fitak et al. 2018 | 0.00001 | 0.968357 | 0.031633 |
| 20 | WO_Minnesota | grey wolf | Minnesota | Fitak et al. 2018 | 0.00001 | 0.730318 | 0.269672 |
| 22 | WO_Minnesota | grey wolf | Minnesota | Fitak et al. 2018 | 0.00001 | 0.728766 | 0.271224 |
| 24 | WO_Minnesota | grey wolf | Minnesota | Fitak et al. 2018 | 0.00001 | 0.73908 | 0.26091 |
| 26 | WO_Minnesota | grey wolf | Minnesota | Fitak et al. 2018 | 0.00001 | 0.718336 | 0.281654 |
| 28 | WO_Minnesota | grey wolf | Minnesota | Fitak et al. 2018 | 0.00001 | 0.738717 | 0.261273 |
| 29 | WO_Minnesota | grey wolf | Minnesota | Fitak et al. 2018 | 0.00001 | 0.740019 | 0.259971 |
| 30 | WO_Minnesota | grey wolf | Minnesota | Fitak et al. 2018 | 0.00001 | 0.724732 | 0.275258 |
| 31 | WO_Minnesota | grey wolf | Minnesota | Fitak et al. 2018 | 0.00001 | 0.755243 | 0.244747 |
| 32 | WO_Minnesota | grey wolf | Minnesota | Fitak et al. 2018 | 0.00001 | 0.732675 | 0.267315 |
| 33 | WO_Minnesota | grey wolf | Minnesota | Fitak et al. 2018 | 0.00001 | 0.745599 | 0.254391 |
| 34 | WO_Minnesota | grey wolf | Minnesota | Fitak et al. 2018 | 0.00001 | 0.736 | 0.26399 |
| 35 | WO_Minnesota | grey wolf | Minnesota | Fitak et al. 2018 | 0.00001 | 0.740072 | 0.259918 |
| 37 | WO_Minnesota | grey wolf | Minnesota | Fitak et al. 2018 | 0.00001 | 0.79495 | 0.20504 |
| 38 | WO_Minnesota | grey wolf | Minnesota | Fitak et al. 2018 | 0.00001 | 0.770336 | 0.229654 |
| 39 | WO_Minnesota | grey wolf | Minnesota | Fitak et al. 2018 | 0.00001 | 0.748463 | 0.251527 |
| 109 | 8152 | grey wolf | Mongolia | New Dataset | 0.061628 | 0.880934 | 0.057438 |
| 110 | 8154 | grey wolf | Mongolia | New Dataset | 0.071434 | 0.864802 | 0.063764 |
| 111 | 8155 | grey | Mongolia | New | 0.053461 | 0.887158 | 0.059381 |

| | | | | | | | |
|---|---|---|---|---|---|---|---|
| | | wolf | | Dataset | | | |
| 113 | 8159 | grey wolf | Mongolia | New Dataset | 0.058864 | 0.889892 | 0.051245 |
| 115 | 8161 | grey wolf | Mongolia | New Dataset | 0.078294 | 0.866817 | 0.054889 |
| 118 | 8164 | grey wolf | Mongolia | New Dataset | 0.057504 | 0.884465 | 0.058031 |
| 119 | 8166 | grey wolf | Mongolia | New Dataset | 0.04612 | 0.897967 | 0.055913 |
| 120 | 8167 | grey wolf | Mongolia | New Dataset | 0.045783 | 0.898157 | 0.05606 |
| 121 | 8168 | grey wolf | Mongolia | New Dataset | 0.048863 | 0.887348 | 0.063788 |
| 123 | 8170 | grey wolf | Mongolia | New Dataset | 0.064899 | 0.880887 | 0.054214 |
| 124 | 8172 | grey wolf | Mongolia | New Dataset | 0.059981 | 0.883823 | 0.056196 |
| 127 | 8176 | grey wolf | Mongolia | New Dataset | 0.060187 | 0.892355 | 0.047458 |
| 128 | 8177 | grey wolf | Mongolia | New Dataset | 0.070076 | 0.885792 | 0.044132 |
| 130 | 8181 | grey wolf | Mongolia | New Dataset | 0.052534 | 0.898563 | 0.048903 |
| MT-10 | WO_Montana | grey wolf | Montana | Fitak et al. 2018 | 0.00001 | 0.976152 | 0.023838 |
| MT-11 | WO_Montana | grey wolf | Montana | Fitak et al. 2018 | 0.00001 | 0.969208 | 0.030782 |
| MT-12 | WO_Montana | grey wolf | Montana | Fitak et al. 2018 | 0.00001 | 0.97532 | 0.02467 |
| MT-13 | WO_Montana | grey wolf | Montana | Fitak et al. 2018 | 0.00001 | 0.962521 | 0.037469 |
| MT-14 | WO_Montana | grey wolf | Montana | Fitak et al. 2018 | 0.00001 | 0.961001 | 0.038989 |
| MT-15 | WO_Montana | grey wolf | Montana | Fitak et al. 2018 | 0.00001 | 0.970544 | 0.029446 |
| MT-17 | WO_Montana | grey wolf | Montana | Fitak et al. 2018 | 0.00001 | 0.983988 | 0.016002 |
| MT-5 | WO_Montana | grey wolf | Montana | Fitak et al. 2018 | 0.00001 | 0.971184 | 0.028806 |
| MT-6 | WO_Montana | grey wolf | Montana | Fitak et al. 2018 | 0.00001 | 0.963571 | 0.036419 |
| MT-7 | WO_Montana | grey wolf | Montana | Fitak et al. 2018 | 0.00001 | 0.958795 | 0.041195 |
| MT-8 | WO_Montana | grey wolf | Montana | Fitak et al. 2018 | 0.00001 | 0.977336 | 0.022654 |
| MT-9 | WO_Montana | grey wolf | Montana | Fitak et al. 2018 | 0.00001 | 0.963174 | 0.036816 |
| NK108296 | WO_NewMexico | grey wolf | New Mexico | Fitak et al. 2018 | 0.033016 | 0.822324 | 0.14466 |
| NK226618 | WO_NewMexico | grey wolf | New Mexico | Fitak et al. 2018 | 0.042135 | 0.818764 | 0.139101 |
| WILDMB | 1177 | grey wolf | New Mexico | Fitak et al. 2018 | 0.025527 | 0.831814 | 0.142659 |
| WILD | 1105 | grey wolf | New Mexico | Fitak et al. 2018 | 0.037467 | 0.822962 | 0.139571 |
| X | 1130 | grey wolf | New Mexico | Fitak et al. 2018 | 0.03763 | 0.831244 | 0.131126 |
| MB | 547 | grey | New | Fitak et | 0.05663 | 0.811119 | 0.132251 |

| | | | | | | | |
|---|---|---|---|---|---|---|---|
| | | wolf | Mexico | al. 2018 | | | |
| GR | 431 | grey wolf | New Mexico | Fitak et al. 2018 | 0.049655 | 0.819018 | 0.131328 |
| MB | 60 | grey wolf | New Mexico | Fitak et al. 2018 | 0.030519 | 0.822117 | 0.147364 |
| X | 593 | grey wolf | New Mexico | Fitak et al. 2018 | 0.043787 | 0.819053 | 0.13716 |
| GR | 412 | grey wolf | New Mexico | Fitak et al. 2018 | 0.054262 | 0.813621 | 0.132117 |
| AR | 284 | grey wolf | New Mexico | Fitak et al. 2018 | 0.051542 | 0.822985 | 0.125473 |
| WILD | 1038_2 | grey wolf | New Mexico | Fitak et al. 2018 | 0.03289 | 0.832827 | 0.134283 |
| X | 1064 | grey wolf | New Mexico | Fitak et al. 2018 | 0.020597 | 0.83713 | 0.142273 |
| DOG | Hershey | grey wolf | New Mexico | Fitak et al. 2018 | 0.963742 | 0.023209 | 0.013049 |
| MB | 169 | grey wolf | New Mexico | Fitak et al. 2018 | 0.033908 | 0.823377 | 0.142715 |
| AR | 258 | grey wolf | New Mexico | Fitak et al. 2018 | 0.044234 | 0.829189 | 0.126577 |
| Wolf | 22818 | grey wolf | North America | Vaysse et al. 2011 | 0.010401 | 0.972881 | 0.016718 |
| Wolf | LUb3 | grey wolf | North America | Vaysse et al. 2011 | 0.00001 | 0.904893 | 0.095097 |
| 131 | 8271 | grey wolf | Saudi Arabia | New Dataset | 0.125362 | 0.744139 | 0.130499 |
| 132 | 8274 | grey wolf | Saudi Arabia | New Dataset | 0.126081 | 0.760314 | 0.113606 |
| SEAK-WOLF-11 | WO_SoutheastAK_01A | grey wolf | Southeast Alaska | Fitak et al. 2018 | 0.00001 | 0.980226 | 0.019764 |
| 99 | WO_SoutheastAK_01A | grey wolf | Southeast Alaska | Fitak et al. 2018 | 0.00001 | 0.973684 | 0.026306 |
| 115 | WO_SoutheastAK_01B | grey wolf | Southeast Alaska | Fitak et al. 2018 | 0.00001 | 0.986137 | 0.013853 |
| 126 | WO_SoutheastAK_01C | grey wolf | Southeast Alaska | Fitak et al. 2018 | 0.00001 | 0.968432 | 0.031558 |
| 74 | WO_SoutheastAK_01C | grey wolf | Southeast Alaska | Fitak et al. 2018 | 0.00001 | 0.982623 | 0.017367 |
| SEAK-WOLF-10 | WO_SoutheastAK_02zPOWIS | grey wolf | Southeast Alaska | Fitak et al. 2018 | 0.00001 | 0.981667 | 0.018323 |
| 121 | WO_SoutheastAK_02zPOWIS | grey wolf | Southeast Alaska | Fitak et al. 2018 | 0.00001 | 0.98157 | 0.01842 |
| 45 | WO_SoutheastAK_02zPOWIS | grey wolf | Southeast Alaska | Fitak et al. 2018 | 0.00001 | 0.97354 | 0.02645 |
| 154 | WO_SoutheastAK_02zPOWIS | grey wolf | Southeast Alaska | Fitak et al. 2018 | 0.00001 | 0.973975 | 0.026015 |
| 160 | WO_SoutheastAK_02zPOWIS | grey wolf | Southeast Alaska | Fitak et al. 2018 | 0.00001 | 0.984987 | 0.015003 |
| 65 | WO_SoutheastAK_03Z | grey wolf | Southeast Alaska | Fitak et al. 2018 | 0.00001 | 0.969316 | 0.030674 |
| 101 | WO_SoutheastAK_03Z | grey wolf | Southeast Alaska | Fitak et al. 2018 | 0.00001 | 0.976157 | 0.023833 |
| 146 | WO_SoutheastAK_03Z | grey wolf | Southeast Alaska | Fitak et al. 2018 | 0.00001 | 0.975118 | 0.024872 |
| 167 | WO_SoutheastAK_03Z | grey | Southeast | Fitak et | 0.00001 | 0.973051 | 0.026939 |

| | | | | | | | |
|---|---|---|---|---|---|---|---|
| | | wolf | Alaska | al. 2018 | | | |
| 173 | WO_SoutheastAK_03Z | grey wolf | Southeast Alaska | Fitak et al. 2018 | 0.00001 | 0.963268 | 0.036722 |
| Wolf4(Female) | WO_Wyoming | grey wolf | Wyoming | Fitak et al. 2018 | 0.00001 | 0.967576 | 0.032414 |
| Wolf6(Female) | WO_Wyoming | grey wolf | Wyoming | Fitak et al. 2018 | 0.00001 | 0.968146 | 0.031844 |
| WY-1 | WO_Wyoming | grey wolf | Wyoming | Fitak et al. 2018 | 0.00001 | 0.962943 | 0.037047 |
| WY-10 | WO_Wyoming | grey wolf | Wyoming | Fitak et al. 2018 | 0.00001 | 0.968922 | 0.031068 |
| WY-12 | WO_Wyoming | grey wolf | Wyoming | Fitak et al. 2018 | 0.00001 | 0.974251 | 0.025739 |
| WY-13 | WO_Wyoming | grey wolf | Wyoming | Fitak et al. 2018 | 0.00001 | 0.951734 | 0.048256 |
| WY-14 | WO_Wyoming | grey wolf | Wyoming | Fitak et al. 2018 | 0.00001 | 0.975107 | 0.024883 |
| WY-15 | WO_Wyoming | grey wolf | Wyoming | Fitak et al. 2018 | 0.00001 | 0.966837 | 0.033153 |
| WY-17 | WO_Wyoming | grey wolf | Wyoming | Fitak et al. 2018 | 0.00001 | 0.976553 | 0.023437 |
| WY-2 | WO_Wyoming | grey wolf | Wyoming | Fitak et al. 2018 | 0.00001 | 0.967862 | 0.032128 |
| WY-3 | WO_Wyoming | grey wolf | Wyoming | Fitak et al. 2018 | 0.00001 | 0.978019 | 0.021971 |
| WY-4 | WO_Wyoming | grey wolf | Wyoming | Fitak et al. 2018 | 0.00001 | 0.96238 | 0.03761 |
| WY-5 | WO_Wyoming | grey wolf | Wyoming | Fitak et al. 2018 | 0.00001 | 0.966045 | 0.033945 |
| WY-7 | WO_Wyoming | grey wolf | Wyoming | Fitak et al. 2018 | 0.00001 | 0.967251 | 0.032739 |
| WY-9 | WO_Wyoming | grey wolf | Wyoming | Fitak et al. 2018 | 0.00001 | 0.964663 | 0.035327 |
| 149 | 6874 | grey wolf | Yakutia | New Dataset | 0.032536 | 0.948547 | 0.018918 |
| 150 | 6875 | grey wolf | Yakutia | New Dataset | 0.028381 | 0.950577 | 0.021042 |
| 151 | 6876 | grey wolf | Yakutia | New Dataset | 0.030096 | 0.940561 | 0.029343 |
| 152 | 6878 | grey wolf | Yakutia | New Dataset | 0.030179 | 0.943253 | 0.026568 |
| 153 | 6881 | grey wolf | Yakutia | New Dataset | 0.030785 | 0.941106 | 0.028108 |
| 154 | 6882 | grey wolf | Yakutia | New Dataset | 0.043706 | 0.924106 | 0.032188 |
| 155 | 6884 | grey wolf | Yakutia | New Dataset | 0.023442 | 0.955063 | 0.021495 |
| 156 | 6885 | grey wolf | Yakutia | New Dataset | 0.024964 | 0.944752 | 0.030285 |
| 157 | 6889 | grey wolf | Yakutia | New Dataset | 0.033302 | 0.935581 | 0.031117 |
| 158 | 6892 | grey wolf | Yakutia | New Dataset | 0.025455 | 0.947207 | 0.027337 |
| 159 | 6893 | grey wolf | Yakutia | New Dataset | 0.029175 | 0.945407 | 0.025419 |
| 160 | 6895 | grey wolf | Yakutia | New Dataset | 0.026811 | 0.95134 | 0.02185 |
| 161 | 6896 | grey | Yakutia | New | 0.029904 | 0.947376 | 0.022721 |

| | | | | | | | |
|---|---|---|---|---|---|---|---|
| | | wolf | | Dataset | | | |
| 162 | 6897 | grey wolf | Yakutia | New Dataset | 0.022206 | 0.951847 | 0.025947 |
| 163 | 6910 | grey wolf | Yakutia | New Dataset | 0.031315 | 0.945408 | 0.023277 |
| 164 | 6926 | grey wolf | Yakutia | New Dataset | 0.027659 | 0.944996 | 0.027345 |
| 165 | 6927 | grey wolf | Yakutia | New Dataset | 0.027373 | 0.949113 | 0.023514 |
| 166 | 6928 | grey wolf | Yakutia | New Dataset | 0.027463 | 0.950881 | 0.021656 |
| 167 | 6932 | grey wolf | Yakutia | New Dataset | 0.027084 | 0.952626 | 0.02029 |
| 168 | 6933 | grey wolf | Yakutia | New Dataset | 0.026203 | 0.949171 | 0.024627 |
| 169 | 6935 | grey wolf | Yakutia | New Dataset | 0.035318 | 0.93411 | 0.030571 |
| 170 | 6937 | grey wolf | Yakutia | New Dataset | 0.02779 | 0.953265 | 0.018945 |
| 171 | 6940 | grey wolf | Yakutia | New Dataset | 0.035728 | 0.923797 | 0.040475 |
| 172 | 6941 | grey wolf | Yakutia | New Dataset | 0.029982 | 0.956298 | 0.013721 |
| 173 | 6944 | grey wolf | Yakutia | New Dataset | 0.034902 | 0.946078 | 0.01902 |
| 174 | 6945 | grey wolf | Yakutia | New Dataset | 0.033589 | 0.934595 | 0.031816 |
| 175 | 6951 | grey wolf | Yakutia | New Dataset | 0.025329 | 0.945169 | 0.029503 |
| 176 | 8893 | grey wolf | Yakutia | New Dataset | 0.028779 | 0.940565 | 0.030657 |
| 177 | 8894 | grey wolf | Yakutia | New Dataset | 0.032203 | 0.931421 | 0.036376 |
| 178 | 8896 | grey wolf | Yakutia | New Dataset | 0.025545 | 0.958817 | 0.015639 |
| 179 | 8906 | grey wolf | Yakutia | New Dataset | 0.032565 | 0.940272 | 0.027163 |
| 180 | 8907 | grey wolf | Yakutia | New Dataset | 0.028775 | 0.939928 | 0.031297 |
| 181 | 8909 | grey wolf | Yakutia | New Dataset | 0.02832 | 0.951449 | 0.020232 |
| 182 | 8910 | grey wolf | Yakutia | New Dataset | 0.033073 | 0.939628 | 0.027299 |
| 183 | 8912 | grey wolf | Yakutia | New Dataset | 0.03069 | 0.956213 | 0.013097 |
| 184 | 8913 | grey wolf | Yakutia | New Dataset | 0.027832 | 0.954936 | 0.017232 |
| 185 | 8914 | grey wolf | Yakutia | New Dataset | 0.02857 | 0.950855 | 0.020575 |
| Taimyr | Taimyr1 | grey wolf, ancient | Taimyr | Skoglund et al. 2015 | 0.0902 | 0.741481 | 0.168318 |
